# Living Hydrogels: Harnessing Microorganism–Material Synergy for Next‐Generation Therapeutics

**DOI:** 10.1002/advs.202521766

**Published:** 2026-04-02

**Authors:** Shuifang Mao, Bingyao Bai, Xingqian Ye, Yiliang Lin

**Affiliations:** ^1^ Department of Chemical and Biomolecular Engineering National University of Singapore Singapore Singapore; ^2^ College of Biosystems Engineering and Food Science Zhejiang Key Laboratory for Agro‐Food Processing Zhejiang Engineering Laboratory of Food Technology and Equipment Zhejiang University Hangzhou China; ^3^ College of Food Science and Engineering Tarim University Alar China; ^4^ Zhejiang University Zhongyuan Institute Zhengzhou China

**Keywords:** biological application, dynamic interaction, living hydrogels, synergistic mechanism

## Abstract

Microorganism‐based therapies, particularly those utilizing probiotics, have emerged as a powerful biomedical strategy owing to their inherent living functionalities. These living systems can dynamically interact with host environments and self‐regulate their activity, offering superior adaptability, prolonged functionality, and microenvironmental responsiveness compared to conventional non‐living therapeutic platforms. Despite these advantages, the direct administration of probiotics faces several challenges, such as poor viability, limited retention at target sites, and the inability to control therapeutic effects in a spatiotemporally precise manner. To address these challenges, embedding probiotics within hydrogel matrices has proven effective in enhancing microbial stability, prolonging in vivo retention, and enabling precise and sustained therapeutic delivery through synergistic interactions between the hydrogels and living microorganisms. This review provides a comprehensive overview of the materials and design strategies employed in the construction of living microorganism‐encapsulated hydrogels (living hydrogels), with particular emphasis on the dynamic interactions and synergistic mechanisms of hydrogel‐microorganism systems. We further illustrate how these mechanisms can achieve various biomedical applications, such as modulating gut microbiota to treat gastrointestinal disease and accelerate wound healing, or leveraging microbial‐induced immune regulation for effective cancer therapy. Finally, the current challenges and future directions associated with the clinical translation of living hydrogels are highlighted. Therefore, the unique multifunctionality and therapeutic promise of living hydrogels position them as compelling candidates for the development of next‐generation biomaterials with unprecedented therapeutic potential.

## Graphical abstract

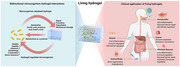

## 1 Introduction

The human microbiome is a fundamental regulator of physiological homeostasis, influencing immune modulation, metabolic equilibrium, and the structural integrity of mucosal barriers [1]. Accumulating evidence has revealed that disturbances in this intricate microbial ecosystem, referred to as dysbiosis, are closely linked to the onset and progression of numerous complex diseases, including inflammatory bowel disease, metabolic disorders, and even cancer [2, 3]. These findings have sparked growing interest in therapeutic strategies aimed at restoring microbial balance to maintain or reestablish health. Among these, the administration of beneficial microorganisms, particularly well‐characterized probiotic strains, has emerged as a promising approach to modulate host physiology in a dynamic and adaptive manner [4]. These living microorganisms can colonize the gastrointestinal tract, sense and respond to biochemical cues within the local microenvironment, interact with host cells, and regulate immune and metabolic pathways through self‐sustaining biological mechanisms [5]. For instance, *Bifidobacterium breve* NCC2950 could effectively alleviate colitis by suppressing pro‐inflammatory cytokine production and reducing inflammatory macrophage infiltration [6] while oral administration of *Bifidobacterium* has shown tumor‐suppressive effects by remodeling the tumor microenvironment and enhancing antitumor immune responses [7]. Beyond their local effects, these microorganisms exert systemic physiological influences by engaging immune, endocrine, neural, and metabolic networks. These influences are mediated through both direct and indirect mechanisms. Direct mechanisms include the restructuring of gut microbial communities and the local secretion of antimicrobial peptides, which can reinforce barrier function and modulate local immune responses. Indirect mechanisms involve the regulation of inflammatory cytokine profiles and the production of microbial metabolites that influence host neuroendocrine signaling pathways [8, 9]. Many of these metabolites can enter systemic circulation and reach distal organs, thereby enabling long‐range communication between the gut and other physiological systems. Interorgan communication axes, such as the oral‐gut, gut‐liver, gut‐brain, and gut‐skin axes, provide mechanistic frameworks for understanding how microbial signals originating in the intestine regulate immunity, neurological function, metabolic homeostasis, and epithelial integrity in peripheral tissues. These axes collectively illustrate how localized microbial activity can translate into coordinated, system‐level physiological outcomes across multiple organ systems [10, 11, 12]. Despite these promising outcomes, direct oral delivery of microorganisms still faces major challenges—such as poor survival in harsh gastrointestinal conditions, transient retention at disease sites, and limited control over their spatial and temporal activity—hindering their full therapeutic potential [13].

To effectively overcome the limitations associated with the direct administration of microorganisms, delivery strategies must account for the inherent biological heterogeneity among distinct microorganism types. The challenges encountered during storage, administration, and in vivo function are not uniform, but instead originate from differences in cellular structure, metabolic activity, and environmental adaptability [14]. Microbial therapeutics encompass diverse biological categories, including probiotic bacteria, yeasts, and filamentous fungi, each characterized by unique structural features, physiological resilience, and modes of host interaction. Probiotic bacteria are generally sensitive to environmental stress during storage and oral delivery, where exposure to oxygen, temperature fluctuations, gastric acidity, bile salts, and digestive enzymes can substantially compromise viability before reaching target sites. Consequently, delivery systems designed for probiotics must prioritize protection against harsh conditions while preserving metabolic activity and colonization potential [15]. In contrast, certain yeasts and filamentous fungi exhibit comparatively greater intrinsic resistance due to their robust cell wall architecture and adaptive stress response mechanisms. For these microorganisms, material design may emphasize spatial localization, controlled growth regulation, or modulation of host interactions rather than primarily focusing on survival protection [16]. Therefore, a rational delivery strategy requires explicit differentiation of microorganism types and careful alignment of material properties with their specific biological and functional demands. Within this context, integrating microorganisms within hydrogel matrices to form living hydrogels has emerged as a versatile and modular solution capable of accommodating these diverse requirements. Rather than serving as a uniform protective shell, the hydrogel matrix can be engineered to provide microorganism specific microenvironments tailored to distinct functional objectives. This hybrid configuration protects microbial viability while enabling sustained release, site‐specific localization, and stimulus‐responsive therapeutic activity. Hydrogels—hydrated polymer networks with high biocompatibility, permeability, and mechanical flexibility—provide an ideal microenvironment for microbial encapsulation [17, 18]. Their high‐water content (ranging from 70% to 99% by volume) ensures sufficient nutrient and gas diffusion, essential for maintaining microbial viability and biological activity [19]. The polymer framework, formed through physical entanglement or chemical crosslinking, imparts mechanical stability yet allows selective transport of solutes and signaling molecules. Depending on design needs, hydrogel matrices can be fabricated from natural, synthetic, or microbially derived polymers, with tunable composition and crosslinking density to regulate microbial behavior [20, 21].

Beyond molecular composition, structural engineering in hydrogels across multiple length scales—achieved through photolithography, micromolding, or 3D printing—offers precise control over pore geometry and matrix architecture [22]. Such spatial regulation allows for precise control over pore size, geometry, and matrix dimensionality, which in turn influence microbial behavior through mechanical constraints, spatial confinement, and mass transport properties. Importantly, these engineered microenvironments not only support microbial viability but also enable the programming of functional microbial outputs such as metabolite production, quorum sensing, and coordinated collective behavior. Moreover, the interaction between microbes and hydrogels is reciprocal: microorganisms can remodel the surrounding matrix and, through genetic modification, endow the material with adaptive or bioactive functions [23]. Collectively, these material design strategies provide the structural and functional foundation of living hydrogels as versatile platforms for microorganism.

The integration of living microorganisms with hydrogel scaffolds has unlocked new possibilities for biomedical applications. Living hydrogels have shown considerable promise in therapeutic contexts, including inflammatory bowel disease management, wound healing, cancer therapy, and other complex pathologies [24, 25, 26]. Their efficacy stems from the synergistic interplay between encapsulated microbes and the hydrogel matrix, which markedly enhances the biological performance of probiotics. For example, in a dextran sulfate sodium (DSS)‐induced colitis mouse model, Zhang et al. reported that a living hydrogel containing probiotics enhanced microbial colonization and alleviated disease symptoms more effectively than free probiotic administration [27]. Similarly, in chronic wound healing, *Lactobacillus* species are widely employed for their lactic acid production, which lowers local pH and suppresses pathogenic bacteria such as *Pseudomonas aeruginosa*. However, direct application of free *Lactobacillus* in immunocompromised burn patients might pose a risk of infection [28]. To mitigate this, Ming et al. developed a living hydrogel composed of *Lactobacillus reuteri* and hyaluronic acid that preserved probiotic efficacy while ensuring safe and localized wound treatment [29].

A key distinction of living hydrogels from conventional biomaterials lies in the synergistic bioactivity between the hydrogel matrix and encapsulated microorganisms. Beyond serving as physical carriers and hydration matrices, hydrogels act as prebiotic scaffolds, supplying nutrients and molecular cues that sustain microbial viability and metabolic function. This mutualistic relationship enhances therapeutic efficacy through dynamic feedback between material and microbe. Moreover, many hydrogel components inherently exhibit biological functions, such as antioxidant, anti‐inflammatory, and mucosal barrier‐restoring effects, which further amplify the system's overall therapeutic performance [30, 31]. This multidimensional synergy endows living hydrogels with robustness, precision, and adaptability, establishing them as a transformative platform for next‐generation, clinically translatable living materials [32, 33, 34]. Understanding and optimizing these cooperative mechanisms is essential to unlocking the biomedical potential of living hydrogels. Achieving this requires an integrated perspective that links material design with microbial physiology.

Over the past several years, a growing number of reviews has explored living hydrogels as multifunctional platforms that integrate microorganisms within hydrogel networks. These reviews have primarily focused on several foundational aspects, including the design of hydrogel matrices with tunable physicochemical properties to enable effective microbial encapsulation; the impact of matrix architecture, porosity, and mechanical properties on microbial viability and function; and the potential biomedical applications of these systems in wound healing, tissue regeneration, and therapeutic delivery [35, 36, 37, 38, 39, 40]. More recently, emerging studies have begun to explore reciprocal interactions between encapsulated microbes and their surrounding hydrogel environments, particularly with respect to metabolic regulation and environmental responsiveness. Despite these advances, existing reviews often present a fragmented perspective, with material design and biological activity treated largely in isolation. A comprehensive framework that captures the dynamic, bidirectional, and synergistic interplay between hydrogel scaffolds and living microorganisms remains lacking. Such an integrative perspective is nevertheless essential for advancing the rational design, functional optimization, and translational development of these hybrid living systems.

There, this review provides a unified overview of living hydrogels as dynamic material–microbe hybrids. We first summarize recent advances in hydrogel materials and fabrication strategies for microbial encapsulation, emphasizing how network architecture, composition, and mechanical properties influence microbial viability and activity. We then discuss the reciprocal interactions between materials and microorganisms that give rise to emergent biofunctions, including self‐regulated metabolism, signal transduction, and adaptive therapeutic responses. Subsequently, key biomedical applications—spanning gastrointestinal disorders, wound healing, and cancer therapy—are highlighted to illustrate the translational potential of these hybrid systems. Finally, we identify current challenges and outline future directions focused on immunogenicity, biosafety, design standardization, and clinical translation. Together, this review aims to provide a conceptual and design‐oriented foundation for the emerging field of living hydrogels, offering guiding principles to support their rational engineering, functional programming, and biomedical translation. It advances an integrated perspective that bridges material dynamics with microbial responsiveness, emphasizing the importance of co‐optimizing structural, chemical, and biological parameters across multiple length and time scales. In particular, the review outlines strategic directions for future development, with the aim of accelerating the maturation of living hydrogels into intelligent, robust, and clinically applicable platforms.

## 2 Materials For Living Hydrogels Design

Within living materials, hydrogels serve as versatile carriers for microbial encapsulation, providing a hydrated 3D microenvironment that supports microbial viability, metabolic activity, and resistance to environmental stress. These hydrogels can be broadly classified by their polymeric origin into natural biopolymers, synthetic biopolymers, and interpenetrating polymer networks (IPNs).

Hydrogels derived from natural biopolymers, including polysaccharides (e.g., alginate, hyaluronic acid, chitosan) and proteins (e.g., gelatin, collagen), inherently provide cell‐adhesive motifs and bioactive signals that promote microbial colonization, quorum sensing, and matrix remodeling (Table 1) [32, 41, 42, 43]. Their biocompatibility and degradability make them particularly attractive for biomedical applications. However, their susceptibility to hydrolytic and enzymatic degradation often limits mechanical integrity and long‐term stability. In addition, batch‐to‐batch variability and limited mechanical strength can compromise reproducibility and scalability for clinical or industrial deployment. In contrast, synthetic hydrogels, such as poly (vinyl alcohol)‐based and polyacrylamide‐based systems, exhibit highly tunable physicochemical properties and mechanical robustness, allowing systematic and precise modulation of microenvironment that governs microbial activity and signaling. Yet, the lack of intrinsic biological functionality often restricts their ability to maintain long‐term microbial viability and bioactivity. To overcome these limitations, recent advances have focused on the development of IPN hydrogels that combine the advantages of both natural and synthetic components. By integrating distinct polymeric networks into a single cohesive matrix, IPNs achieve mechanical robustness while preserving biocompatibility and introducing responsiveness to environmental cues [44, 45]. These hybrid structures not only enhance structural stability but also support dynamic interactions with encapsulated microorganisms. Consequently, IPN‐based living hydrogels hold great promise as next‐generation microbial delivery platforms capable of interacting with host tissues, modulating the gut microenvironment, and enabling precisely controllable therapeutic interventions.

**TABLE 1:** Representative living hydrogel materials.

Matrix components	Advantages	Disadvantages	Supporting force	Applications
Sodium alginate	High hydrophilicity, biocompatible, adjustable viscosity, adjustable degradation rate, antibacterial	Lack of mammalian cell adhesion, insufficient cell adhesion sites	Electrostatic complexion, salt bridging, coupled gel network	Encapsulate *Bacillus licheniformis* [54], *Lactobacillus casei* [55]
Hyaluronic acid	High water absorbability, adjustable elasticity, antioxidant, anti‐inflammatory, anticancer, promote angiogenesis, cell targeting	Poor mechanical characteristics, rapid degradation	Hydrogen bond, dynamic covalent chemistry, michael‐addition reaction of sulfhydryl group with acrylate, dynamic covalent chemistry	Encapsulate *Lactobacillus reuteri* [31, 63]
Chitosan	Biodegradable, biocompatible, adjustable degradation rate, promotes blood coagulation and wound healing, antibacterial, antitumor, adhesive properties	Poor solubility at physiological pH, low mechanical strength	Cysteine‐modified, metal coordination interaction, dynamic covalent chemistry	Encapsulate *Bifidobacterium bifidum FL‐276.1* [58], *Lactobacillus reuteri* [59]
Collagen	High water absorbability, good biocompatibility, low immunogenicity, adhesive properties	Low mechanical strength, rapid enzymatic degradation	Self‐assembly	Encapsulate *Lactobacillus fermentum*, and *Lactobacillus acidophilus* [70]
Gelatin	Amphoteric compound, thermal reversibility, adjustable viscosity, anti‐inflammatory, antibacterial, promote chronic wound healing, low antigenicity, high number of integrin binding sites	Low mechanical strength, rapid enzymatic degradation	Dynamic covalent chemistry, enzymatically crosslinked by an mTGase enzyme, glutaraldehyde crosslinking, chain entanglement	Encapsulate *Lactobacillus plantarum* [74], *Lactiplantibacillus plantarum spp*. [75] *Kluyveromyces lactis* [76]
Silk fibroin	Hydrophilicity, good biocompatibility, biodegradable, adjustable mechanical properties, adjustable degradation rate, genetically modifiable polymer, promote wound healing	Slow and poorly controlled gelation kinetics	Interpenetrating networks	Encapsulate *Pseudomonas aeruginosa* PAO1 [81]
Curli	Good biocompatibility, biodegradable, adjustable degradation rate, extensively genetically modifiable, antibacterial, low immunogenicity	Weak mechanical properties	Covalent crosslinking chemistry	Encapsulate non‐pathogenic *E. coli* strain (PQN4) [84]
PEG	Biocompatibility, hydrophilic nature, flexible chain structure, adjustable swelling capacity, low immunogenicity	Intrinsic bio‐inertness, limited degradability	Chemical crosslinking by PEG	Encapsulate *Lactobacillus* strains [95]
PAAm	High‐water affinity, tunable mechanical performance, swelling capability, broad chemical modifiability	Intrinsic bio‐inertness, non‐degradability, and potential cytotoxicity from residual monomers or crosslinkers	Chemical crosslinking by MBAA	Encapsulate genetically modified *Escherichia coli* (98] )
Pluronic F127	Biocompatibility, injectability, shear thinning, gelation by heating to room temperature, chemically modifiable	Low mechanical strength	Chain entanglement	Encapsulate genetically modified *Escherichia coli* (103] )
Interpenetrating polymer network	High‐water affinity, biocompatibility, injectability, adjustable mechanical properties, adjustable viscosity, structural uniformity, promote wound healing	Complex fabrication procedures	Dynamic covalent chemistry, chain entanglement, chain entanglement	Encapsulate genetically engineered *Escherichia coli* Nissle 1917, [23] *Lactobacillus paracasei LS14*[109]

### 2.1 Polysaccharide‐Based Living Hydrogels

In designing polysaccharide‐based living hydrogels, it is essential to account for the inherent ionic nature of each polysaccharide, as this characteristic fundamentally shapes their gelation behavior and compatibility with microbial encapsulation. The net charge—whether anionic, cationic, or neutral—not only influences the choice of crosslinking strategy but also governs the resulting network architecture. For example, alginate, an anionic polysaccharide, undergoes ionic gelation through interactions with divalent cations like calcium, leading to the formation of mechanically stable yet highly porous matrices [46]. Hyaluronic acid, another anionic polymer, exhibits weaker ionic interactions and thus often requires chemical modification to support covalent or dynamic covalent crosslinking [47]. On the other hand, cationic polysaccharides such as chitosan typically form hydrogels via complexation with multivalent anionic crosslinkers like tripolyphosphate [48]. In contrast, neutral polysaccharides—such as guar gum, or dextran—ack strong electrostatic interactions and are generally crosslinked through physical entanglements, hydrogen bonding, or mild chemical modifications (e.g., methacrylation) to enable hydrogel formation [49, 50]. These distinct gelation pathways and charge‐dependent interactions impact not only the mechanical integrity and porosity of the resulting hydrogels but also the biochemical and biophysical microenvironment surrounding encapsulated microorganisms. Consequently, they play a critical role in regulating microbial viability, metabolic activity, stress tolerance, and overall functional performance within living hydrogel systems.

*Alginate‐Based Living Hydrogels*. Alginate‐based living hydrogels have attracted considerable attention as microbial carriers due to their biocompatibility, high oxygen permeability, and mild gelation under physiological conditions [51]. Structurally, alginate consists of β‐D‐mannuronic acid (M) and α‐L‐guluronic acid (G) residues arranged in homopolymeric (MM or GG) and heteropolymeric (MG) blocks, which assemble into 3D hydrogel networks through ionic crosslinking with divalent cations such as calcium [52]. The M/G ratio and block distribution critically determine the hydrogel's mechanical strength, porosity, and network stability, which in turn influence microbial viability by modulating nutrient diffusion and metabolite transport [53]. Recent studies illustrate how these molecular designs can shape microbial behavior. For example, Chen et al. developed a sodium alginate hydrogel microsphere system containing metal‐phenolic nanoparticle‐modified halloysite clay and probiotics, creating a dynamic living interface that provided mechanical protection during gastrointestinal transit while maintaining metabolic compatibility. The system achieved a probiotic survival rate of 81.7% after 2 h under simulated gastrointestinal conditions [54]. Similarly, Xin et al. implemented a hierarchical encapsulation approach, immobilizing *Lactobacillus casei* within alginate microspheres subsequently embedded in a bulk alginate matrix. This multiscale structure facilitated biofilm‐like self‐assembly, localized metabolite enrichment, and long‐term microbial viability, leading to improved microbiota modulation and therapeutic outcomes [55]. These findings highlight alginate's versatility as a tunable matrix for creating stable, metabolically compatible, and spatially organized living systems.

*Chitosan‐Based Living Hydrogel*. Chitosan‐based hydrogels have emerged as multifunctional platforms for microbial encapsulation owing to their intrinsic microbiota‐regulating capacity, pH‐responsiveness, and versatile crosslinking capabilities [56]. Derived from the partial deacetylation of chitin, chitosan is a linear cationic polysaccharide with abundant amino and hydroxyl groups, which enables diverse crosslinking chemistries such as ionic (e.g., with tripolyphosphate), covalent (e.g., via aldehyde‐functionalized polymers), metal coordination, and supramolecular interactions [57]. Such tunability supports both preformed and injectable hydrogels with adjustable porosity, mechanical strength, and degradation kinetics—key for protecting microorganisms while enabling controlled release. Functional modifications further expand chitosan's capability. For example, Luo et al. fabricated cysteine‐modified chitosan microspheres via microfluidic encapsulation of *Bifidobacterium bifidum* FL‐276.1, which resisted gastric acid and achieved intestinal release. Notably, they could form disulfide bonds with cysteine residues in mucus, thereby achieving effective adhesion between microorganisms and intestinal mucosa [58]. Likewise, hybrid injectable hydrogels formed by carboxylated chitosan and oxidized hyaluronic acid were used to encapsulate *Lactobacillus reuteri* shielded with metal–phenolic nanofilms, maintaining microbial viability under gentamicin exposure while preserving lactic acid secretion [59]. Collectively, these examples demonstrate how the chemical versatility and biological responsiveness of chitosan can be exploited to construct adaptive living materials capable of precise microbial delivery under complex physiological conditions.

*Hyaluronic Acid‐Based Living Hydrogel*. Hyaluronic acid (HA)‐based hydrogels represent structurally tunable and biologically interactive matrix for encapsulating and delivering living microorganisms [60]. As a natural glycosaminoglycan of the extracellular matrix, HA consists of alternating N‐acetyl‐D‐glucosamine and glucuronic acid units and can be sourced from animal tissue or microbial fermentation. Its intrinsic viscoelasticity, high water retention, and bioadhesive properties support mucosal adhesion and microenvironmental regulation [61]. Abundant hydroxyl and carboxyl groups allow diverse chemical modifications—including oxidation, thiolation, and dynamic covalent crosslinking—enabling flexible network design [62]. For instance, boric acid‐modified HA‐based hydrogels exhibited rapid gelation under acidic gastric conditions, forming a protective matrix that significantly improved microbial survival [30]. In another study, methacrylated HA crosslinked with thiolated thioketal enabled reactive oxygen species (ROS)‐responsive release of *Lactobacillus reuteri* in the colon, improving disease‐targeted activation and minimizing off‐target effects [63]. Beyond serving as a passive scaffold, HA actively contributed to immune modulation and inflammation attenuation by promoting macrophage polarization from the pro‐inflammatory M1 phenotype to the reparative M2 phenotype, suppressing pro‐inflammatory cytokine secretion, and supporting mucosal barrier restoration. These attributes collectively confer distinct advantages for gut‐targeted therapeutic applications [63].

### 2.2 Protein‐Based Living Hydrogels

Proteins constitute a versatile class of biomacromolecules for the construction of living hydrogels, offering intrinsic bioactivity, structural tunability, and high biocompatibility with encapsulated microorganisms. Their physicochemical attributes—such as net charge, hydrophobicity, molecular weight, and self‐assembly propensity—critically govern gelation mechanisms and network formation. In particular, neutral proteins can undergo physical gelation through non‐covalent interactions, including hydrogen bonding, hydrophobic association, and β‐sheet aggregation, without the need for chemical crosslinkers [64, 65, 66] Cationic proteins such as poly‐L‐lysine enable electrostatic complexation with anionic biopolymers to form hydrogels under mild aqueous conditions [67]. This gentle gelation process is advantageous for preserving microbial viability and maintaining functional activity. Nonetheless, variations in amino acid sequence, molecular weight, and secondary structural features across different protein types necessitate customized material strategies to ensure effective microbial encapsulation, stable biological integration, and controlled release under physiological conditions.

*Collagen‐Based Living Hydrogels*. Collagen‐based hydrogels, derived from the major structural protein of the extracellular matrix, have attracted considerable attention as carriers for living microorganisms owing to their excellent biocompatibility, low immunogenicity, and ability to mimic native biochemical signaling [68]. Collagen spontaneously forms hydrogels through pH‐ and temperature‐dependent self‐fibrillogenesis under physiological conditions, facilitating easy preparation and clinical handling. Unlike polysaccharide‐based scaffolds, collagen inherently contains triple‐helical ligands and arginine‐glycine‐aspartic acid (RGD) sequences, which promote microbial adhesion and mediate interactions between the host and embedded microorganisms. These bioactive interactions enhance microbial viability and preserve metabolic activity under physiological stress [69]. For instance, collagen hydrogels encapsulating *Lactobacillus fermentum* and *Lactobacillus acidophilus* exhibited improved acid resistance and strong adherence to mucosal surfaces in intravaginal delivery. These protective effects were mainly attributed to the hydrogel microarchitecture, which conferred mechanical stability while preserving microbial metabolic activity, thereby supporting a sustained colonization profile conducive to restoring microbiota balance. Importantly, this configuration offered a novel localized and antibiotic‐free therapeutic strategy for managing bacterial vaginosis [70].

*Gelatin‐Based Living Hydrogels*. Gelatin, a natural polymer obtained through the partial hydrolysis of collagen, is increasingly recognized as a promising material for the construction living hydrogels due to its favorable biocompatibility, biodegradability, and tunable functional properties [71]. Specifically, gelatin can be biodegraded by cell‐derived enzymes such as matrix metalloproteinases (MMPs), promoting cell‐mediated matrix remodeling through extracellular matrix protein deposition and network rearrangement induced by cell contractility. Although gelatin lacks the triple‐helical structure of native collagen, its abundant functional groups (e.g., carboxyl and amino groups) facilitate chemical crosslinking and physical gelation under mild conditions suitable for microbial encapsulation [72]. Through rational design, functional derivative such as amide base‐crosslinked gelatin has been developed to generate mechanically tunable and viscoelastic 3D networks that are well‐suited to preserving the viability and bioactivity of encapsulated microorganisms [73]. For example, a dihydrazide‐modified gelatin hydrogel encapsulating *Lactobacillus plantarum* preserved over 80% of probiotic viability while minimizing bacterial leakage. The hydrogel also created a localized microbiota regulatory microenvironment, effectively suppressing the growth of pathogens commonly associated with wound infections, including *Pseudomonas aeruginosa*, *Staphylococcus aureus*, and *Candida albicans* [74]. To enhance mechanical strength and environmental resilience, gelatin has also been combined with materials such as alginate, poly(vinyl alcohol), or graphene oxide, yielding composite hydrogels with improved thermal stability, pH responsiveness, and controlled microbial release under gastrointestinal conditions [75, 76]. Consequently, they achieved high microbial survival rates under simulated gastric and intestinal conditions, while enabling controlled release modulated by the degree of network cross‐linking and swelling ratio.

*Silk Fibroin‐Based Living Hydrogels*. Silk fibroin, a natural fibrous protein derived from *Bombyx mori* cocoons, has emerged as a promising material for microbial encapsulation due to its intrinsic biocompatibility, mechanical resilience, and controllable biodegradability [77]. Its densely packed β‐sheet crystalline domains impart high tensile strength and tunable degradation, allowing prolonged microbial retention and controlled release [78]. Compared to alginate or other rapidly eroding matrices, silk fibroin hydrogels maintain structural integrity under physiological conditions, supporting sustained microbial viability [79]. To overcome the limited bioactivity of pure silk fibroin, composite hydrogels have been created by integrating adhesive domains or combining fibroin with collagen or gelatin. For instance, silk fibroin‐collagen hydrogels integrated the mechanical stability of silk fibroin with the cell‐adhesive properties of collagen, thereby establishing a supportive microenvironment that sustained the proliferation and metabolic functionality of encapsulated microorganisms over extended periods [80]. Similarly, silk fibroin methacryloyl combined with alginate formed composite hydrogels mimicking the architecture and physicochemical properties of natural extracellular polymeric substances, supporting microbial growth and functional stability in dynamic environments [81]. Collectively, these advances underscore the versatility of silk fibroin hydrogels as mechanically robust and biologically adaptive carriers for living microbial delivery.

*Curli‐Based Living Hydrogels*. Curli‐based hydrogels, formed by the self‐assembly of nanofibrous proteins secreted by *Escherichia coli*, offering a structurally robust and genetically programmable platform for living microbial encapsulation and delivery [82]. These hydrogels exhibit modular architecture, and adjustable biological interfaces, making them highly attractive for engineering living therapeutic systems. Unlike traditional polysaccharide hydrogels, curli fibers are proteinaceous amyloids that can be fused with functional peptides or enzymatic domains, thereby enabling precise customization of biochemical surface properties to match diverse therapeutic or environmental requirements [83]. For example, Duraj‐Thatte et al. developed a scalable fabrication strategy that concentrated curli‐producing cultures through vacuum filtration, followed by sodium dodecyl sulfate‐induced crosslinking to generate a stable hydrogel network. The resultant hydrogels sustained long‐term microbial retention in the gastrointestinal tract and supported continuous in situ microbial synthesis of therapeutic molecules, emphasizing their potential as dynamic and durable living microbial delivery systems [84]. Beyond passive encapsulation, the genetic programmability of curli nanofibers allows fusion with functional peptides or targeting ligands, enabling spatially selective adhesion to intestinal epithelia and enhanced host–microbe compatibility [85]. This molecular flexibility positions curli‐based hydrogels as a new generation of living materials that integrate autonomous metabolic activity, immune modulation, and tissue interfacing within a single, programmable framework.

### 2.3 Synthetic Polymer‐Based Living Hydrogels

*Polyethylene Plycol (PEG)‐Based Living Hydrogels*. Polyethylene glycol (PEG) is a cost‐effective, water‐soluble polymer consisting of repeating ethylene oxide units, typically synthesized through the ring‐opening polymerization of ethylene oxide using hydroxyl initiators to yield polymers of 0.4–100 kDa [86]. Owing to its high hydration capacity, conformational flexibility, biocompatibility, and amphiphilic nature, PEG exhibits minimal steric hindrance at the molecular level [87]. These features make PEG an ideal candidate for hydrogel fabrication using various strategies, including radiation‐induced crosslinking [88], free‐radical polymerization of acrylated derivatives [89], or chemical approaches such as Michael addition, click reactions, and condensation chemistry [90]. PEG‐based hydrogels are valued by their non‐toxicity, biocompatibility, and minimal immunogenicity, which collectively make them highly suitable for biomedical application [91]. Importantly, their structural properties, including porosity, swelling behavior, and degradation kinetics, can be precisely regulated by modulating the degree of polymer branching and crosslinking density [92]. However, native PEG lacks biological recognition sites, limiting cellular or microbial interactions. Functionalization with adhesive peptides (for example, RGD), glycosaminoglycans, or bioactive polymers, enables precise regulation of cell adhesion, differentiation, and immune responses [93]. Such modifications are particularly crucial for maintaining microbial viability and activity under physiological stress [94]. For instance, the composite PEG hydrogels functionalized with γ‐polyglutamic acid exhibited enhanced mechanical resilience while generating localized buffering microenvironments that shielded encapsulated microorganisms from acidic conditions. These hybrid hydrogels not only preserved microbial viability but also facilitated efficient mucosal delivery and sustained probiotic function [95]

*Polyacrylamide (PAAm)‐Based Living Hydrogels*. Poly(acrylamide) (PAAm) is a hydrophilic polymer containing amide groups in each repeating unit, synthesized via free‐radical polymerization of acrylamide monomers in aqueous environments. The resulting 3D crosslinked networks display high water retention, tunable mechanics, and versatile chemical modifiability, making PAAm‐based hydrogels useful synthetic matrices for constructing living materials [96]. These properties are particularly important for sustaining the viability and metabolic activity of encapsulated microorganisms under physiologically relevant conditions [97]. In addition, the structural flexibility of PAAm facilitates the design of composite hydrogels with enhanced mechanical robustness and biological functionality. For instance, integrating ionically crosslinked alginate within a covalently crosslinked PAAm network produced a robust double‐network hydrogel capable of withstanding large deformations and supporting the stable proliferation of engineered *Escherichia coli* [98]. Furthermore, under alkaline conditions, PAAm underwent hydrolysis to form polyelectrolyte networks, resulting in volume expansion—a property that has been exploited in designing stimuli‐responsive hydrogels capable of adapting dynamically to environmental changes [99]. Despite these advantages, residual acrylamide monomers may exhibit cytotoxicity; thus, rigorous purification and synthesis protocols are essential for biomedical use [100].

*Pluronic F127‐Based Living Hydrogels*. Pluronic F‐127 is a thermoresponsive triblock copolymer composed of polyethylene oxide‐polypropylene oxide‐polyethylene oxide segments, exhibiting a reversible sol‐gel transition near physiological temperatures [101]. This feature allows uniform microbial encapsulation at low temperatures, followed by rapid in situ gelation, achieving spatially localized and stable microbial retention [102]. With its injectability, biocompatibility, and ease of handling, Pluronic F‐127 has emerged as a promising matrix for living hydrogel systems. For instance, a bilayer thin‐film hydrogel composed of Pluronic F‐127 and its acrylate derivative enabled spatial compartmentalization of bacterial growth and light‐induced protein expression, both governed by the crosslinking degree of each layer [103]. Beyond microbial integration, Pluronic F‐127 has been utilized as a carrier for adipose‐derived stem cells [104], mesenchymal stem cell‐derived exosomes [105], and antibacterial molecules such as phillyrin [106], demonstrating its multifunctionality in biomedical applications.

### 2.4 Interpenetrating Polymer Network

Interpenetrating polymer network (IPN) hydrogels integrate two or more independently crosslinked polymer networks within a single matrix, stabilized by physical entanglements or secondary interactions rather than direct covalent bonds between the constituent networks [107]. This architectural strategy enables the independent but synergistic modulation of key parameters such as stiffness, viscoelasticity, and biological function, which are often difficult to achieve in conventional single‐network systems [108]. As a result, IPN hydrogels provide robust yet responsive scaffolds ideally suited for microbial encapsulation and sustained bioactivity. For instance, an IPN hydrogel composed of gelatin methacryloyl and hyaluronic acid methacryloyl was shown to dynamically respond to the specific microenvironment of bone defects. Within this system, microbial viability was maintained, and the encapsulated engineered *Escherichia coli* Nissle 1917 successfully released metabolites in a controlled manner [23]. Notably, extending this concept to plant‐derived biopolymers, protein‐pectin IPN hydrogels formed via enzyme‐mediated crosslinking of soy protein isolate and sugar beet pectin exhibited enhanced probiotic *Lactobacillus paracasei LS14* protection and mechanical adaptability under gastrointestinal stress [109]. Despite these advances, key challenges remain in achieving precise inter‐network compatibility and uniform crosslinking. Unintended phase separation or premature gelation may compromise matrix integrity and hinder functional performance. Future progress will rely on rational molecular design, predictive computational modeling, and standardized fabrication protocols to ensure network homogeneity and reproducibility, thereby fully realizing the potential of IPN hydrogels as next‐generation living material platforms.

## 3 Strategies for Encapsulating Microorganisms

**FIGURE 1:**
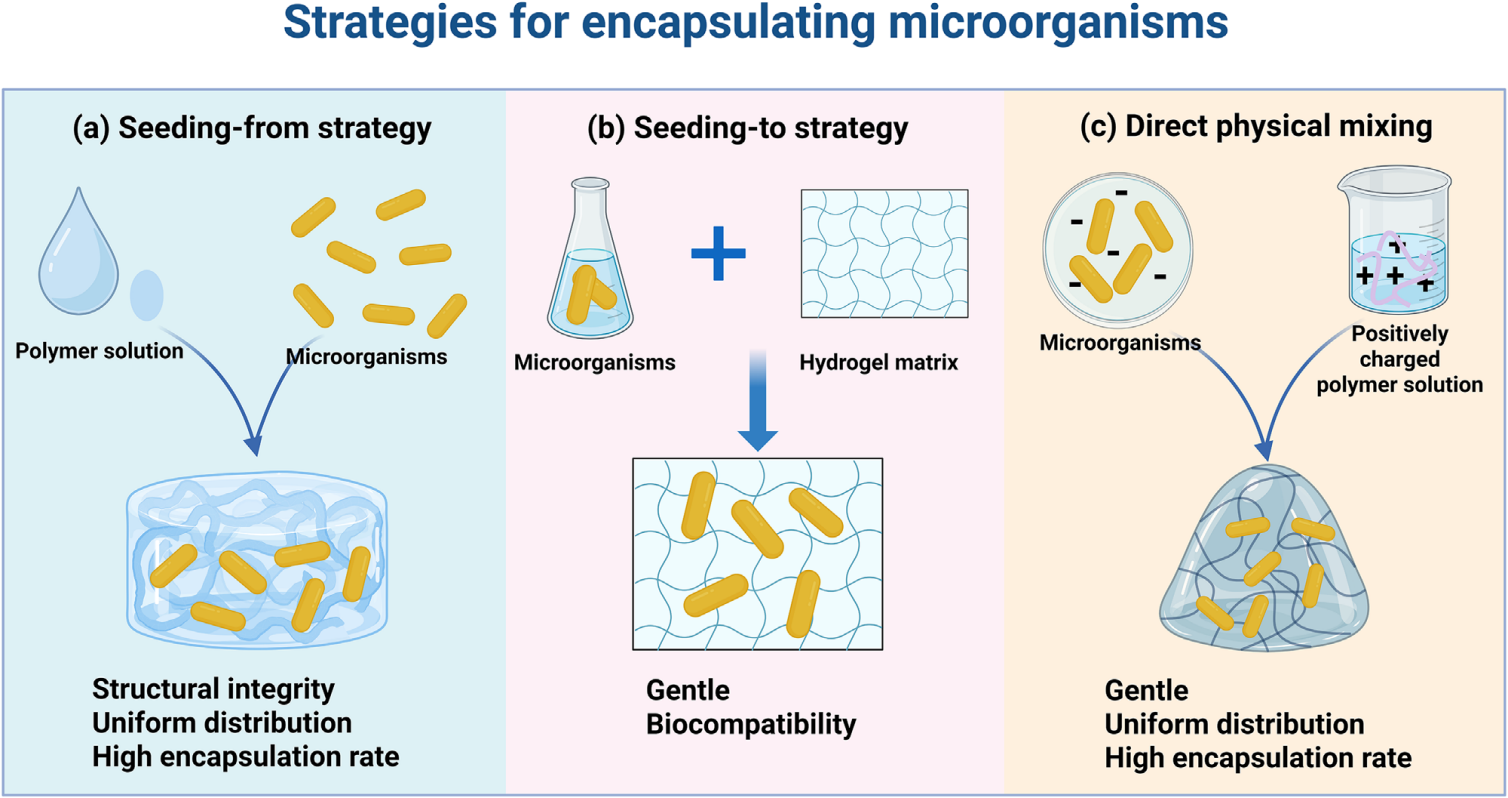
Three strategies for encapsulating microorganisms in hydrogel networks. (a) Seeding‐from strategy: microorganisms are mixed with polymer solution before crosslinking into hydrogel. (b) Seeding‐to strategy: microorganisms are introduced to preformed hydrogel. (c) Electrostatic self‐assembly: negative charged microorganisms mixed with positive charged polymer solution to form hydrogel.

Once hydrogel matrices with appropriate mechanical, physicochemical, and biological properties have been designed, the next crucial consideration is how living microorganisms are incorporated into these systems. Encapsulation strategies generally fall into three main categories: (i) incorporation during hydrogel formation (seeding‐from), (ii) introduction into preformed networks (seeding‐to), and (iii) electrostatic self‐assembly (Figure 1). Each approach offers distinct advantages and trade‐offs in terms of encapsulation efficiency, microbial viability, spatial distribution, and matrix integrity (Table 2). In the seeding‐from strategy, microorganisms are dispersed within the precursor solution prior to gelation, allowing homogeneous distribution throughout the hydrogel. However, exposure to physicochemical stresses during polymerization or crosslinking may compromise microbial viability. Conversely, the seeding‐to approach—introducing microbes into preformed hydrogels—minimizes exposure to harsh conditions but often results in uneven colonization and limited long‐term retention. Electrostatic self‐assembly offers a milder and more controllable alternative, enabling microorganisms to interact with charged polymeric networks via surface electrostatics, though maintaining uniformity and mechanical stability remains challenging. Regardless of the encapsulation route, residual crosslinking agents or reactive intermediates might disrupt microbial metabolism or elicit undesired biological responses. Therefore, the development of cytocompatible, structurally stable, and reproducible encapsulation platforms is essential for advancing living hydrogel technologies toward reliable and clinically relevant biomedical applications.

**TABLE 2:** Survival improvement of microorganisms by different encapsulation strategy.

Encapsulate strategy	Encapsulation efficiency	Survival rate enhancement vs. unencapsulated			Applicable scenarios
		In simulate gastric juice	In simulate intestinal fluid	Under long‐term refrigeration conditions	
Seeding‐From	90%–100% [113, 114, 115]	3–6X	3–6X	1–4X	Exposes microorganism to shear/thermal/pH stress, enables uniform loading and rapid prototyping
Seeding‐To	80%–85% [98, 121, 122]	2–3X	2–3X	2–3X	Prone to cell leakage, suitable for strains vulnerable to gelation‐induced stress
Electrostatic Self‐Assembly	90%–100% [124, 125]	5000–10000X	2–3X	3–4X	Enables site‐specific delivery, suitable for targeted release and surface functionalization

### 3.1 Seeding‐From Strategy

Seeding‐from strategies encapsulates probiotics during hydrogel formation and represent one of the most widely applied approaches for constructing living hydrogels. This method ensures high encapsulation efficiency and relatively uniform spatial distribution of microorganisms throughout the polymer network, facilitating reproducible performance and structural integrity. Moreover, because microorganisms are embedded directly within the forming network, this strategy is compatible with large‐scale manufacturing processes and enables the integration of probiotics into a wide range of hydrogel matrices, including both synthetic and naturally derived polymers [110, 111]. Such characteristics make seeding‐from particularly advantageous when robust immobilization and initial microbial retention are required. However, the same process that promotes efficient encapsulation can also compromise cell viability, as microorganisms are directly exposed to the physicochemical stresses associated with gelation. The cytocompatibility of crosslinking chemistry thus becomes a critical determinant of long‐term microbial function. Conventional covalent approaches, such as radical‐initiated polymerization of acrylated precursors under ultraviolet or redox initiation, provide controllable gelation kinetics and tunable mechanical strength. Yet, reactive intermediates and residual initiators can cause oxidative stress, membrane disruption, and reduced metabolic activity, thereby limiting their utility for sensitive probiotic strains [112]. Consequently, increasing attention has been directed toward cytocompatible gelation strategies that rely on reversible or noncovalent interactions to minimize cellular stress during encapsulation.

Electrostatic interactions between oppositely charged polymer chains provide a mild, reversible, and cytocompatible means of constructing living hydrogels. Such polyelectrolyte complexation enables network formation without the need for chemical initiators or harsh reaction conditions, thereby reducing cellular stress during encapsulation. For instance, Ni et al. reported that hydrogels formed via the electrostatic complexation of sodium alginate and gelatin effectively encapsulated *Lactobacillus plantarum* [113]. Nevertheless, electrostatically assembled matrices often lack sufficient mechanical robustness under acidic or bile environments, necessitating reinforcement through multivalent ions or interpenetrating polymer networks [114]. Hydrogen bonding represents another gentle crosslinking mechanism for probiotic encapsulation. Dense hydrogen‐bonded networks have been engineered by combining polysaccharides such as alginate, konjac glucomannan, or xanthan gum with polyphenolic crosslinkers. Notably, tannic acid, a naturally derived polyphenol rich in hydroxyl groups, has been utilized to construct alginate/pectin‐based hydrogels with enhanced microbial encapsulation efficiency and superior resistance to gastrointestinal stress, due to its capacity to form dense hydrogen bond networks [115]. Moreover, hybrid systems that integrate hydrogen bonding with coordination interactions have yielded mechanically robust hydrogels capable of protecting encapsulated *Bacillus subtilis* from antibiotics and promoting wound repair [116].

Beyond noncovalent systems, dynamic covalent chemistry (DCC) offers yet another versatile approach. Hydrogels formed through reversible imine, hydrazone, or oxime linkages exhibit properties such as injectability, stress relaxation, and environmental responsiveness, which are particularly desirable for adaptive microbial encapsulation [117, 118]. For instance, Schiff base hydrogels constructed from oxidized polysaccharides and chitosan have been shown to maintain the viability of *Lactobacillus plantarum* while simultaneously modulating wound microenvironments through microbial regulation and pH‐regulating effects [119]. Other DCC motifs, such as boronic ester–catechol interactions, confer dual responsiveness to pH and sugar concentrations, expanding the functional landscape of dynamic microbial encapsulation systems [120].

Despite these advances, the seeding‐from approach still faces an inherent trade‐off between structural integrity and microbial viability. Excessive crosslinking may hinder nutrient diffusion and waste removal, whereas insufficient network density can lead to premature microbial leakage. Moreover, variations in gelation kinetics and local microenvironmental conditions challenge reproducibility and scalability. A deeper mechanistic understanding of how polymer architecture, crosslinking chemistry, and matrix microenvironments collectively regulate microbial physiology will be critical for guiding the rational design of next‐generation living hydrogels that unify structural fidelity, cytocompatibility, and functional adaptability for biomedical applications.

### 3.2 Seeding‐To Strategy

In “seeding‐to” strategies, probiotics are introduced into hydrogels after the matrix has been formed, thereby avoiding direct exposure to potentially cytotoxic radical polymerization or chemical crosslinking reactions. Unlike in situ encapsulation, where microorganisms may be compromised by harsh gelation conditions, seeding‐to strategy allows microorganisms loading under mild environments. This feature make it particularly suitable for incorporating fragile or stress‐sensitive probiotic strains [98, 121]. For instance, Zhu et al. fabricated silk fibroin/chitosan microgels and subsequently introduced probiotics via electrostatic interactions between the positively charged chitosan and negatively charged microbial surfaces [122]. This approach not only provides a gentle and flexible means of introducing single or multiple strains into the same scaffold but also permits post‐fabrication modulation of microbial composition, a feature that is highly relevant for constructing adaptive probiotic consortia. The long‐term performance of seeding‐to‐encapsulation, however, is governed by the physicochemical properties of the host hydrogel. Surface hydrophilicity and chemical functionality dictate microbial adhesion and colonization, while pore size and interconnectivity control nutrient diffusion, metabolite exchange, and waste removal. However, because microbial loading occurs after gelation, cell distribution within the matrix is often heterogeneous, resulting in uneven colonization and variable functional outputs. Moreover, passive diffusion limits microbial infiltration in dense or poorly connected networks, while the absence of strong bioadhesive interactions can lead to gradual cell detachment and loss of viability over time [35]. Overall, while the seeding‐to strategy offers exceptional cytocompatibility and flexibility, optimizing hydrogel architecture to enhance microbial adhesion, uniformity, and nutrient accessibility remains a critical challenge. Addressing these limitations through bioadhesive functionalization, hierarchical porosity design, and dynamic remodeling mechanisms could enable more stable and spatially controlled microbial colonization in future living hydrogel systems.

### 3.3 Electrostatic Self‐Assembly

In contrast to the seeding‐from strategy—where microorganisms are passively embedded by mixing them with pre‐gel polymer solutions and subsequently immobilized during hydrogel formation via physical or chemical crosslinking—electrostatic self‐assembly offers a fundamentally different paradigm. Rather than serving as inert fillers, microorganisms in this approach actively contribute to hydrogel architecture through direct electrostatic interactions with oppositely charged polymers [123]. This process does not necessarily require the formation of a fully crosslinked network; instead, structural cohesion is achieved through spontaneous complexation driven by electrostatic attraction. Electrostatic self‐assembly provides a distinctive and cytocompatible approach for constructing living hydrogels by leveraging the intrinsic negative surface charge of microbial cells to form spontaneous complexes with oppositely charged polymers. This process eliminates the need for chemical initiators or harsh reaction conditions, offering a mild alternative to conventional covalent crosslinking. The anionic character of microbial membranes, primarily derived from ionized phosphate and carboxylate groups, enables stable interactions with cationic polymers such as chitosan, glycol chitosan, and polyacrylamide derivatives [124]. These interactions facilitate gentle microbial encapsulation and permit tunable control over hydrogel architecture through modulation of polymer charge density and distribution. However, the success of electrostatic assembly depends critically on the balance between interaction strength and cellular compatibility. Weak electrostatic interactions can lead to unstable encapsulation and premature microbial release, whereas overly strong binding may disrupt microbial membranes and impair viability. For instance, hydrogels enriched with cationic groups may bind too strongly to negatively charged microbial membranes, leading to membrane disruption and eventual cell death [125]. Antimicrobial peptides and quaternary ammonium compounds exemplify this effect, as their high charge density has been shown to trigger microbial lysis [126, 127]. Therefore, precise control over polymer charge density, molecular weight, and ionic strength of the surrounding environment is essential to achieve stable yet biocompatible assembly. Rationally designed electrostatic systems that integrate spatial charge patterning, multivalent coordination, or dynamic charge switching could further expand the design space of living hydrogels, enabling robust microbial encapsulation and controlled release in probiotic and therapeutic applications.

## 4 Emerging Technologies in Living Hydrogel Fabrication

A variety of fabrication techniques, including emulsion polymerization, template molding, and electrostatic assembly, have been employed to construct microorganism‐loaded hydrogels. While these conventional approaches offer structural diversity and relative simplicity, they often suffer from limited control over spatial resolution, scalability, and microenvironmental precision. Such limitations become increasingly restrictive when living hydrogels are required to support long‐term microbial viability, spatial organization, and programmable biological function.

In recent years, microfluidics, 3D bioprinting, and electrospraying have emerged as powerful enabling platforms for engineering living hydrogels with precisely defined architectures, tunable functionalities, and high microbial compatibility. Unlike traditional bulk‐processing methods, these technologies provide enhanced spatiotemporal control over material assembly and biological encapsulation, thereby enabling the rational design of microenvironments that better align with microbial physiology and application‐specific requirements. In the following sections, we provide a focused discussion of the fundamental principles, engineering advantages, and representative applications of these emerging fabrication strategies in the context of living hydrogel systems.

### 4.1 Microfluidics

Microfluidic‐based fabrication of microorganism‐loaded living hydrogels has emerged as a powerful and versatile platform for constructing precisely structured and biologically functional materials [128]. By leveraging the unique characteristics of microscale fluid dynamics—such as laminar flow, interfacial tension, and dominance of viscous forces at low Reynolds numbers—microfluidics allows for highly controlled manipulation of fluid streams within micron‐sized channels. This facilitates the reproducible formation of microgels, and compartmentalized architectures that encapsulate microorganisms under mild, cytocompatible conditions [129]. Compared with conventional techniques such as template molding, emulsion polymerization, or dispersion polymerization, which often suffer from limited control over particle monodispersity and structural uniformity, microfluidic platforms offer substantially enhanced precision and flexibility [130, 131]. Through rational design of chip geometry and fine tuning of flow rates, microfluidic systems enable high‐throughput fabrication of living hydrogels with well‐defined sizes, shapes, internal architectures, and compositions. This tunability is critical for optimizing microbial encapsulation efficiency, nutrient diffusion, metabolic exchange, and overall functional performance [132, 133]. In addition, microfluidics excels at emulsifying and encapsulating multiple immiscible fluids within confined geometries, enabling the generation of isotropic microgels, Janus particles, and anisotropic or compartmentalized structures via mechanisms such as Rayleigh‐Plateau instability [134, 135].

Several microfluidic formats have been developed for living hydrogel fabrication, including microchannel‐based microfluidics, microchamber‐based microfluidics, and droplet microfluidics. Among these, droplet microfluidics is the most widely adopted. In this approach, probiotic suspensions are mixed with hydrogel precursors as the dispersed phase and emulsified into uniform droplets within a continuous phase, followed by ionic, thermal, or photo‐crosslinking to form stable living microgels [130]. This strategy offers exceptional control over droplet size, morphology, and microbial payload distribution. Importantly, the mild processing conditions and spatial precision of microfluidic encapsulation help preserve microbial viability while enabling targeted and functional delivery. For example, Zheng et al. designed a microbial cocktail comprising three strains with complementary metabolic functions, which were encapsulated via droplet microfluidics into calcium alginate microcapsules and coated with a polydopamine layer. The spatial confinement and semi‐permeable structure not only retained microbial viability but also ensured functional activity in degrading uremic toxins under physiological stress [136]. Similarly, Wang et al. fabricated a dual‐probiotic hydrogel with spatially segregated microdomains through droplet microfluidics, achieving controlled localization and sustained metabolite release at infected wound sites, while maintaining functional activity in a complex biological microenvironment [137].

Beyond single‐phase encapsulation, advanced microfluidic architectures—such as flow‐focusing and T‐junction devices—enable the fabrication of multifunctional microgels that co‐encapsulate probiotics together with prebiotics or postbiotics to achieve synergistic therapeutic effects [138, 139, 140]. The resulting constructs offer tunable porosity, high structural uniformity, and efficient nutrient diffusion, thereby supporting metabolic exchange and cooperative microbial activity. For example, Yang et al. developed a novel synergistic hydrogel system composed of *E. coli* Nissle 1917 and postbiotics through microfluidics‐based approach to achieve synergistic therapeutic effect [138]. Yin et al. fabricated a composite hydrogel system combining *E. coli* Nissle 1917, sodium alginate, inulin, and other prebiotics, which showed significant efficacy in treating inflammatory bowel disease and colitis‐associated colorectal cancer [140]. Collectively, these studies illustrate how microfluidics enables precise control over living hydrogel microarchitecture and composition, thereby providing a versatile platform for engineering functionally programmable microbial materials.

### 4.2 3D Bioprinting

Recent advances in 3D bioprinting have substantially expanded the capabilities for fabricating spatially organized and biologically active hydrogels that encapsulate living microorganisms. In contrast to microfluidic techniques, which are particularly effective for producing monodisperse microscale droplets, 3D bioprinting offers greater macroscopic flexibility in controlling construct geometry, spatial patterning, and the distribution of biological components. This capability makes it especially well suited for fabricating patterned probiotic constructs, microbial biosensors, antimicrobial dressings, and engineered systems for environmental bioremediation [141].

3D bioprinting strategies are commonly classified into two major categories: material deposition‐based printing and light‐assisted printing. The former includes extrusion‐based and inkjet‐based techniques, which rely on shear‐thinning, non‐Newtonian bioinks that decrease in viscosity under shear stress to enable smooth deposition through fine nozzles. Following deposition, printed structures undergo rapid solidification either autonomously or in response to external stimuli such as ultraviolet irradiation. In contrast, light‐assisted techniques—including stereolithography and digital light processing—use spatially controlled ultraviolet exposure to induce localized photopolymerization in photosensitive bioinks. These approaches offer fine spatial resolution and enable construction of hierarchical architectures that emulate tissue‐like complexity [142, 143].

Among these, extrusion‐based bioprinting is the most widely utilized due to its ease of operation and broad compatibility with various hydrogel‐based bioinks. By tuning parameters such as nozzle diameter, extrusion flow rate, and the viscoelastic properties of the ink, this technique enables high‐resolution and structurally stable printing [144]. Frequently utilized bioinks include soft and biocompatible hydrogels such as hyaluronic acid, silk fibroin, gelatin, Pluronic F127, alginate, and polyethylene glycol diacrylate. These materials enable mild gelation conditions that preserve microbial viability while maintaining excellent structural fidelity.

The choice of hydrogel matrix plays a pivotal role not only in determining the mechanical integrity and diffusion properties of the printed construct but also in sustaining microbial activity and functional expression. Through rational selection and formulation, these hydrogels can be tailored to create microenvironments that support the encapsulation and proliferation of various microorganisms—including bacteria, algae, and spores—thereby enabling the fabrication of engineered living materials with programmable functions such as pollutant degradation, biosensing, antimicrobial activity, metabolite production, and bioenergy generation. For instance, Schaffner et al. encapsulated bacteria in a hydrogel composed of biocompatible hyaluronic acid, κ‐carrageenan, and fumed silica through extrusion‐based bioprinting, enabling degrading pollutants and producing medically relevant bacterial cellulose [145]. Jiang et al. reported the encapsulation of microalgae within a 3D double‐network hydrogel formed from polyethylene glycol dienate and sodium alginate, achieving efficient antibiotic degradation [146]. Liu et al. designed a biogenic hydrogel combining microalgae and probiotics for the treatment of diabetic chronic wounds, demonstrating improved healing and antimicrobial properties [147]. Additionally, González et al. fabricated *Bacillus subtilis* spore‐laden agarose hydrogels, which exhibited robust antimicrobial efficacy against methicillin‐resistant *Staphylococcus aureus* [148]. Together, these studies demonstrate how 3D bioprinting enables precise spatial organization of microbes and matrices across multiple length scales, thereby offering a powerful route for constructing architecturally complex and functionally programmable living hydrogel systems.

### 4.3 Electrospraying

Electrospraying provides an alternative and highly scalable approach for fabricating microorganism‐loaded microspheres ranging from micrometer to submicrometer dimensions under gaseous conditions. In this technique, a liquid precursor is delivered through a capillary nozzle and subjected to a high‐voltage electric field. When electrostatic forces overcome the liquid's cohesive surface tension, a fine jet forms and subsequently breaks into microdroplets. These droplets are stabilized and solidified through crosslinking in a reactive collection medium. In addition to electric‐field control, vibrational or pulsation technologies are often integrated to facilitate jet breakup and suppress droplet coalescence, thereby improving size uniformity [149]. Compared with other encapsulation techniques, electrospraying offers high payload efficiency, narrow size distributions, and good scalability. These features have enabled its maturation into a reliable platform for microbial encapsulation. A representative example is provided by Haffner et al., who developed a dual‐layered core‐shell system by combining electrospraying with sol‐gel silica coating to encapsulate *Lactobacillus rhamnosus* GG within alginate‐silica microspheres. The resulting constructs exhibited significantly improved resistance to gastric conditions and enabled colon‐specific release, allowing metabolically dormant cells to regain activity and dominate the colonic microbiota under simulated gastrointestinal conditions [150]. Overall, electrospraying complements microfluidics and 3D bioprinting by offering a high‐throughput and scalable route for producing microencapsulated living hydrogels with robust barrier properties and controlled release profiles. Its integration into the fabrication toolbox further expands the design space for engineering living hydrogel systems tailored for oral delivery and other translational applications.

## 5 Bidirectional Microorganisms‐Hydrogel Interactions

The integration of living microorganisms within hydrogel matrices introduces a new level of functionality that fundamentally transforms conventional encapsulation concepts. Rather than acting as inert carriers, hydrogels and microorganisms form bidirectional, dynamically coupled systems in which each component continuously modulates the structure, composition, and functionality of the other. This reciprocal relationship represents the defining characteristic, and the conceptual cornerstone of emerging living hydrogel technologies.

On one hand, the hydrogel provides a protective and nurturing microenvironment that supports microbial proliferation, colonization, and metabolic activity. The hydrated, 3D matrix stabilizes cell membranes, buffers external stressors, and facilitates the exchange of nutrients and metabolites. Tunable network parameters such as porosity, stiffness, and surface chemistry further regulate microbial behavior, from adhesion and spatial organization to quorum sensing and biofilm formation.

On the other hand, embedded microorganisms actively reshape their material hosts. Through metabolic activity, enzymatic secretion, and microenvironmental modulation (e.g., local pH, ionic, or redox changes), microbes alter the hydrogel's physicochemical and mechanical properties. In natural polysaccharide‐based systems, the matrix itself can serve as a metabolically compatible prebiotic substrate, selectively degraded by embedded probiotics to provide bioavailable carbon sources that sustain microbial activity [35]. These processes create dynamic feedback loops that couple material degradation, nutrient cycling, and microbial growth.

Such mutualistic interactions form the foundation for self‐regulating biohybrid materials that exhibit adaptive responses and autonomous functional evolution. These dynamic couplings between microbial metabolism and material structure underpin the bidirectional effects that define living hydrogels. In the following sections, we will discuss how the hydrogel matrix influences microbial behavior—through its mechanical, chemical, and diffusional properties—and conversely, how microbial activities reshape the hydrogel network via enzymatic remodeling, metabolic products, and local physicochemical changes. Understanding these intertwined processes is key to engineering living materials that bridge synthetic design with biological metabolism, transforming hydrogels from passive scaffolds into active participants in therapeutic and environmental functions

### 5.1 Effect of Hydrogels on Microorganism

#### 5.1.1 Influence of Hydrogel Confinement on Microbial Phenotypes

**FIGURE 2:**
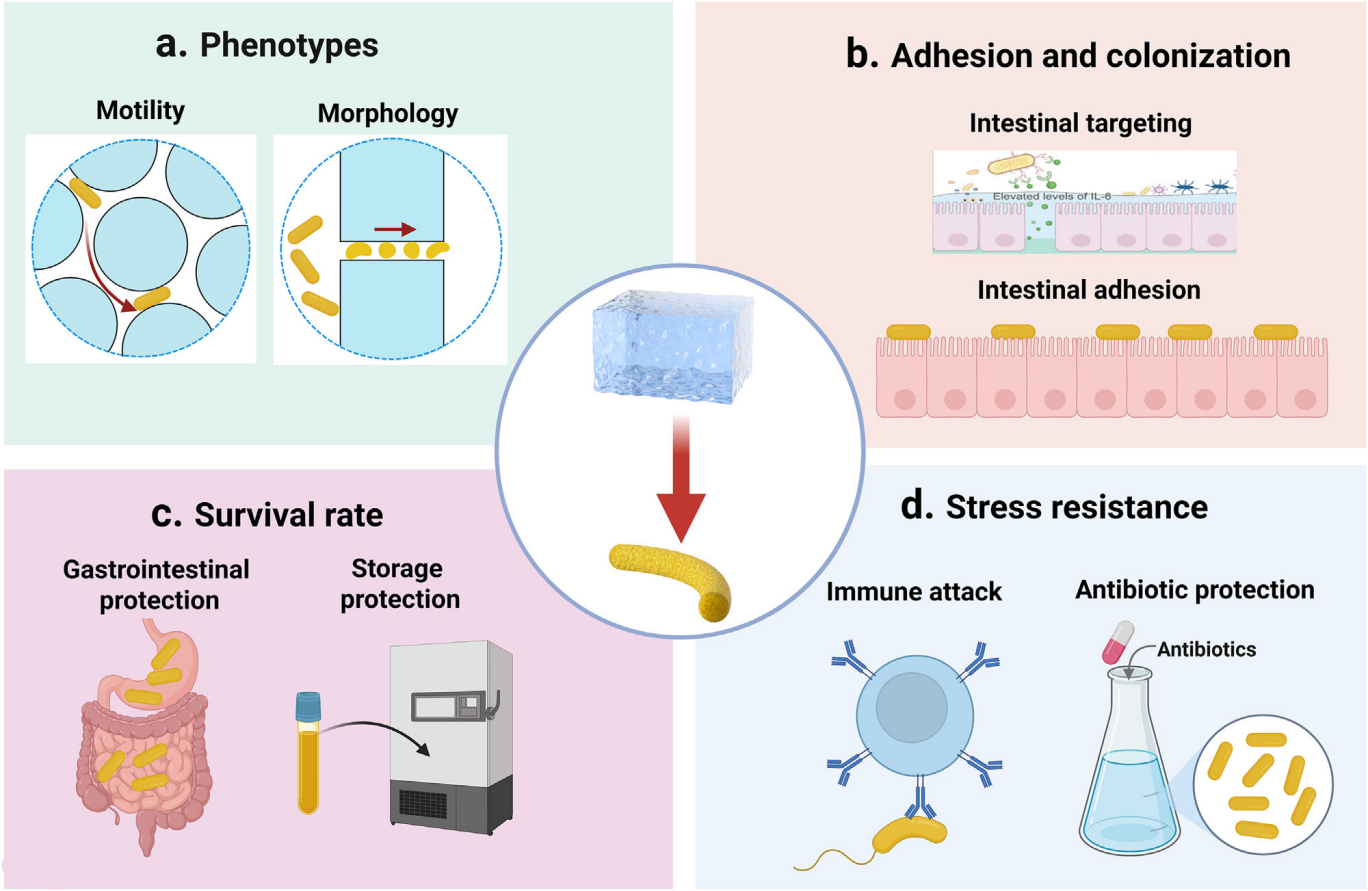
Effect of hydrogels on microorganism. (a) Effect of hydrogels on phenotypes of microorganism. (b) Effect of hydrogels on the survival rate of microorganism. (c) Effect of hydrogel on the stress resistance of microorganism. (d) Effect of hydrogels on the adhesion and colonization of microorganism.

At the single‐cell level, the geometric confinement imposed by the hydrogel matrix exerts a significant influence on microbial motility and spatial organization (Figure 2a). Within porous hydrogel networks, microbial motility typically resembles the classical run‐and‐tumble dynamics observed in aqueous media [151]. However, as matrix density increases, steric constraints alter microbial locomotion. Bhattacharjee et al. reported a transition to a “hop‐and‐trap” motility mode when the pore size ranged between 1.5 and 3.6 µm: during the hopping phase, cells actively navigated through continuous pore channels, whereas in the trapping phase, they remained immobilized for prolonged periods due to steric hindrance and spatial jamming [152]. As the pore size narrows, the effective hop distance correspondingly decreases, further limiting overall cellular displacement. Similarly, in microfluidic confinement studies, Mannik et al. found that flagellar microbial cells still maintained their movement if the channel width was at 1.3 times the cell diameter (e.g., 1.2 µm) [153]. However, when the characteristic dimension of the hydrogel matrix approaches or falls below the microbial cell size (≤1 µm), locomotion is significantly limited. Under these constraints, microbial cells often elongate and proceed to divide in situ rather than actively migrate.

Beyond motility, hydrogel confinement can also modulates microbial morphology. As cells traverse narrow architectures, they undergo mechanical stress that results in sustained elongation and curvature, often leaving persistent morphological imprints even after release from confinement [154]. Takeuchi et al. developed a micro‐embossing strategy to fabricate custom‐shaped chambers (feature size ∼2.0 µm) within agarose hydrogels, which enabled filamentous microorganisms to elongate along predefined geometries, including crescent, zigzag, sine‐wave, and spiral configurations [155]. Similarly, in bioprinted hydrogels, the viscoelastic properties of the matrix were found to regulate yeast colony morphology, expansion dynamics, and proliferation [156].

At the population level, microscale confinement with hydrogel structures acts as a mechanical boundary, limiting cell division and population growth. Under sufficient nutrient supply and low confinement, microbial populations typically undergo exponential expansion, doubling every 20–60 min [157, 158]. However, within confined microchambers (10–20 µm in diameter), microbial proliferation slows dramatically once available space is saturated [159]. Studies using protein‐ and gelatin‐based hydrogel chambers demonstrated that microbial populations reached densely packed states within 10–12 h, after which the growth rate sharply declined due to volumetric limitations. Together, these observations highlight how hydrogel‐induced confinement—through control of pore geometry, stiffness, and viscoelasticity—plays a pivotal role in shaping microbial motility, morphology, and population dynamics. Understanding these spatially governed behaviors is essential for engineering living hydrogels that balance microbial proliferation with structural stability and controlled functionality [160, 161].

#### 5.1.2 Hydrogel‐Mediated Enhancement of Microbial Adhesion and Colonization

In living microorganism‐based therapeutics, suboptimal targeting and limited colonization at lesion sites remain critical impediments to clinical efficacy. Functional hydrogel systems offer innovative strategies to overcome these challenges through precise targeting design and microenvironmental regulation (Figure 2b). The core advantage of these systems lies in their ability to synergistically enhance the adhesion and colonization efficiency of microorganisms at target sites through multiple coordinated mechanisms, thereby achieving synergistic therapeutic outcomes between the hydrogel carrier and the microbial payload [162].

To achieve targeted adhesion and long‐term colonization, hydrogel systems have evolved from simple physical protection toward designs that exploit specific molecular recognition, structural optimization, and adaptive responsiveness. Molecular recognition underpins precise delivery. For example, inflammation targeting hydrogel delivery system utilized the specific binding of IL‐6 aptamers to highly expressed IL‐6 at lesion sites, enhancing probiotic retention by approximately tenfold relative to free cells and prolonging their persistence for over 48 h [33]. Building upon this, composite mechanisms further improve localization stability. A liposome‐microgel hybrid system that combined active targeting through KPV tripeptide‐PEPT1 interactions with passive adhesion mediated by electrostatic attraction between negatively charged microgels and positively charged mucosal immune cells. This multimodal synergy extended probiotic residence time from 24 h to nearly 72 h, highlighting the importance of integrating multiple adhesion mechanisms for durable localization [163]. Environmental responsiveness offers another design dimension enabling adaptive retention under physiological flow and mechanical stress. Thermosensitive hydrogels, for example, exhibit fluidity at room temperature—facilitating uniform spreading over irregular mucosal surfaces—but rapidly form robust 3D networks at physiological temperature, resisting shear and fluid clearance. This behavior enabled sustained retention for over 21 days in the uterine cavity while maintaining probiotic viability and activity in dynamic in vivo environments [164].

Complementary to physical and responsive mechanisms, chemical anchoring strategies provide covalent stabilization at the bio‐interfaces. Sulfhydryl hyaluronid‐based hydrogels, for instance, formed disulfide bonds with mucin proteins in the colon through thiol groups, extending microbial retention up to 24 h under pathological conditions. This covalent anchorage established a robust basis for probiotic colonization under chronic pathological conditions [26]. Beyond adhesion, microenvironmental and ecological modulation further supports long‐term colonization. Targeted delivery ensures precise probiotic transport to diseased sites, while active modulation of local conditions establishes niches favorable to probiotic survival. This design philosophy reflects a shift from “passive adaptation” to “active remodeling.” Ecological niche optimization is a core approach to modulating microbial community structure in favor of probiotic colonization. For example, calcium tungstate microgels released probiotic *Bacillus coagulans* in response to site‐specific stimuli, selectively disrupting the ecological niche occupied by abnormally expanded *Enterobacteriaceae* during colitis to facilitate probiotic colonization. This ecological restructuring created spatial and functional niches conducive to probiotic enrichment [162].

Collectively, these strategies demonstrate that hydrogel systems are not merely protective carriers but dynamic colonization platforms. By integrating targeting, adhesion, responsiveness, and ecological modulation, they enable precise, stable, and functional microbial colonization—laying the groundwork for next‐generation living therapeutics capable of sustained efficacy in complex physiological environments.

#### 5.1.3 Hydrogel‐Mediated Enhancement Survival and Functionality of Microorganisms

Hydrogel‐based encapsulation offers a comprehensive strategy for enhancing the viability and functionality of living microorganisms by providing multidimensional protection against a variety of physiological and environmental stresses (Figure 2c,d) [165]. The key advantages of hydrogels lie in their structural tunability, and functional adaptability, which together create dynamic and protective microenvironments conducive to microbial survival.

A primary mechanism by which hydrogels improve microbial survival is physical barrier protection. Through rational structural design, hydrogels create isolated microenvironments that shield microorganisms from external stressors such as acidity, bile salts, and enzymatic degradation [166]. Porous network architectures exemplify such physical protection strategies. For instance, thiolated hyaluronic acid hydrogels [167] and cellulose microgels [168], not only provide adequate spatial niches for living microbial habitation but also attenuate the permeation of gastrointestinal aggressors such as gastric acid and bile salts. These 3D matrices substantially reduce direct exposure of living microorganism to gastrointestinal fluids. Under simulated digestive conditions, encapsulation within such porous hydrogels increased probiotic survival by 6–7 orders of magnitude compared to free cells [167]. Core–shell architectures further enhance protective efficacy. For instance, a cellulose–alginate core–shell hydrogel integrated a porous inner compartment for microbial loading with an outer pH‐responsive barrier, effectively mitigating gastric erosion and enzymatic degradation. This dual‐layer configuration achieved a 10‐fold increase in intestinal microbial survival compared with conventional alginate‐only carriers [168]. Beyond gastrointestinal protection, hydrogel‐based physical barriers also contribute to the long‐term stability of probiotics during storage. For instance, Ca‐alginate/cryoprotectants/cellulose composite hybrid microgels preserved ≥10^6^ CFU/mL of viable probiotics for 160 days at 4°C, with only a two‐log reduction in survival rate, significantly outperforming conventional alginate carriers [169].

In addition to physical shielding, microenvironmental modulation within hydrogels plays a crucial complementary role in sustaining microbial viability. For example, selecting high internal phase emulsions with oil‐phase content exceeding 80%–90% reduced water activity in the environment, thereby limiting heat induced protein denaturation during high‐temperature treatment (such as pasteurization). This approach improved the survival rate of probiotics encapsulated within microgel by 3‐fold after pasteurization [170]. Additionally, hydroxyl groups within hydrogels formed a neutral microenvironment locally through buffering, which further alleviated the acid damage from gastric acid [171]. Collectively, these findings demonstrate that hydrogel‐based protection of living microorganisms extends far beyond simple encapsulation. It emerges from the synergistic integration of structural barriers and localized environmental regulation, together creating a dynamic, protective niche that sustains microbial viability under physiological and processing stresses. Such multidimensional protection is essential for realizing the full therapeutic potential of living microorganisms in biomedical and gastrointestinal applications.

Hydrogels also serve as immunoprotective barriers in vivo. By limiting direct exposure of encapsulated microbes to host immune cells, hydrogels reduce immune recognition and clearance. For example, in a bone defect model, a bilayer hydrogel composed of porous gelatin methacryloyl microspheres and hyaluronic acid methacryloyl was used to encapsulate engineered *Escherichia coli* Nissle 1917. This structural arrangement reduced direct contact between encapsulated microorganism and host immune cells, thereby mitigating premature clearance and effectively maintaining functional activity [23]. Similarly, in the treatment of infected cutaneous wounds, a dual‐structure system composed of gelatin microspheres and methacrylate‐modified hyaluronic acid hydrogel was employed to encapsulate *Lactobacillus reuteri*. This hydrogel scaffold not only shielded the microbes from immune surveillance but also prevented their uncontrolled release into surrounding tissues, mitigating biosafety risks [163].

In chemically challenging environments, hydrogels provide additional protection by limiting the diffusion of harmful molecules. In the management of multidrug‐resistant wound infections, double‐layered alginate microgels encapsulating *Lactobacillus fermentum* and the angiogenic factor deferoxamine provided spatially separated compartments. The dense alginate shell effectively restricted antibiotic penetration, thereby protecting the probiotic compartment from the invasion of tobramycin. Following antibiotic treatment, the bacterial count of encapsulated *Lactobacillus fermentum* decreased by only one order of magnitude, compared with a three‐order reduction observed for non‐encapsulated probiotic. This partitioned encapsulation preserved both probiotic and growth factor functional activity, underscoring the importance of structural compartmentalization in complex therapeutic environments [172].

Hydrogel encapsulation also mitigates solvent‐induced stress in microbial biocatalysis. For instance, alginate hydrogels significantly reduced the penetration rate of organic solvents such as toluene when encapsulating genetically modified *Escherichia coli*. After 1 h of pure toluene exposure, encapsulated cells retained over 80% of their biocatalytic activity, whereas non‐encapsulated cells completely lost function. The high water content and dense polymeric network of alginate limited solvent diffusion and preserved membrane integrity, effectively overcoming a long‐standing limitation in maintaining microbial activity in biphasic or organic systems [173]. Collectively, these studies illustrate that hydrogel‐based encapsulation provides multifaceted protection against biological, chemical, and solvent stress. By integrating physical isolation with selective permeability and microenvironmental control, hydrogels create a resilient niche that safeguards microbial function, ensuring therapeutic reliability and operational stability across diverse biomedical and biotechnological applications.

Collectively, these studies highlight the multifaceted role of hydrogels as protective carriers for living microbes. Through synergistic integration of physical shielding, immune evasion, microenvironmental regulation, and molecular selectivity, hydrogels establish resilient niches that preserve microbial function across biomedical and biotechnological applications.

### 5.2 Microbial Regulation of Hydrogel Properties

#### 5.2.1 Microbial Control Over Hydrogel Formation

Bacterial cellulose, a type of pure nanocellulose characterized by high mechanical strength, exceptional hydrophilicity and excellent biocompatibility. Certain cellulose‐producing microorganisms, can directly contribute to the fabrication of living hydrogels through their intrinsic metabolic pathways, offering a natural and efficient strategy for biologically driven hydrogel synthesis [174, 175]. For instance, engineered *Escherichia coli* has been reported to produce helically structured nanofibers that establish adhesive hydrogel networks, enabling sustained therapeutic delivery within the gastrointestinal tract [84, 176]. The unique material properties of bacterial cellulose are closely linked to its molecular architecture. Composed of linear β‐1,4‐D‐glucan chains, it shares chemical similarity with plant‐derived cellulose but differs markedly in supramolecular organization. Specifically, bacterial cellulose assembles into an interconnected nanofiber network with water‐retention capabilities that exceed several times its dry weight, enabling hydrogel formation with excellent moisture management [177]. The nanofiber hydrogels exhibit high crystallinity, which translates into superior mechanical performance compared to conventional polysaccharide‐based hydrogels. For instance, when cultured in liquid media, *Gluconacetobacter xylinus* actively synthesizes cellulose nanofibrils at the air‐liquid interface, resulting in the formation of robust bacterial cellulose films. In the hydrated state, these hydrogels exhibit excellent mechanical performance, with Young's moduli ranging from 7 to 12 GPa and tensile strengths between 50 and 200 MPa [178]. Furthermore, the incorporation of additives such as chitosan, gelatin, clay, or silica into the culture medium has been shown to modulate both the composition and mechanical behavior of the resulting bacterial cellulose, offering additional tunability for specific biomedical applications [179].

**FIGURE 3:**
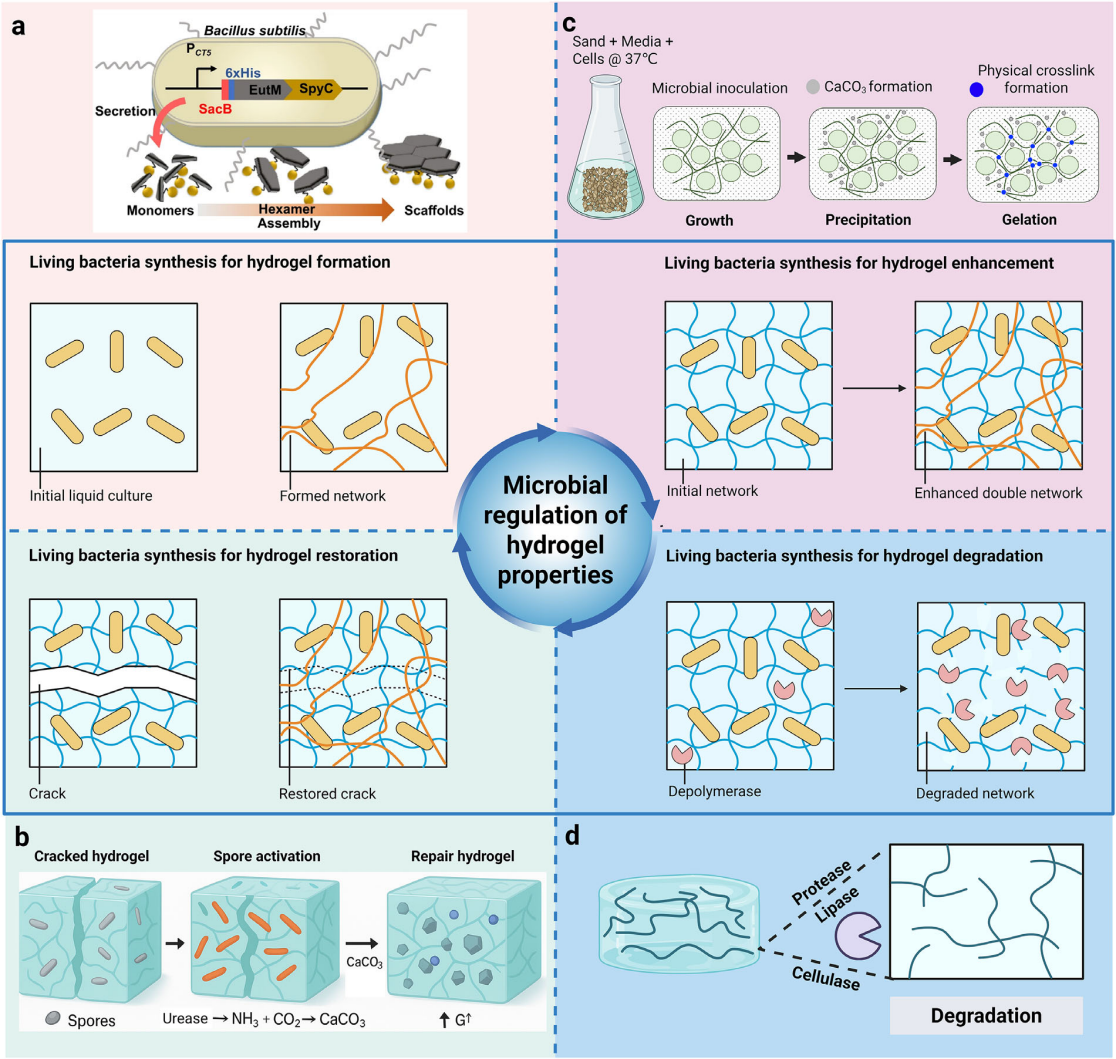
Living microorganism produce biomaterials for hydrogel formation, restoration, mechanical enhancement, and degradation. (a) Engineered *Bacillus subtilis* secretes protein‐based hydrogel matrix building blocks. Reproduced with permission [184]. Copyright 2020, Springer Nature. (b) Microbial spores catalyze the formation of calcium carbonate precipitates to fill cracks and repair hydrogels. (c) The physically crosslinked hydrogel together with bacterial calcite precipitation supports the living hydrogel. Reproduced with permission [186]. Copyright 2020, Elsevier (d) Hydrolases secreted by microorganisms enable degrade hydrogels. Reproduced with permission [196]. Copyright 2013, Elsevier.

Microbial biofilm is a ubiquitous form of aggregated biopolymer generated by microbial cells, mainly composed of extracellular polysaccharides, extracellular DNA, lipids, and proteins, and have a hydrogel‐like structure [180]. These matrix structures can effectively protect microorganisms within the community from the invasion of harsh external environments such as antibiotics and extreme pH values. For instance, compared to bacteria in a planktonic state, cells in biofilms can increase their tolerance to antibiotics by 10 to 1000 times, greatly enhancing the survival ability of microorganisms in harsh environments [181]. In addition to providing protection, microbial biofilms facilitate enhanced microbial adhesion and colonization on host tissues by modulating microbe‐surface interactions. For example, Gui et al. constructed *Lactobacillus* biofilms supported by gelated cell sheet, which significantly improved bacterial colonization in the vaginal mucosa of mice and demonstrated significant therapeutic efficacy in an aerobic vaginitis model [182]. Furthermore, *Lactobacillus rhamnosus* administered in its biofilm state has increased persistence in the intestines of premature rat pups, thereby improving the therapeutic effect of *Lactobacillus rhamnosus* on necrotizing enterocolitis [183]. Advances in synthetic biology have further expanded the functional scope of microbes in hydrogel engineering. Genetically modified *Bacillus subtilis* strains have been successfully programmed to secrete proteinaceous matrices that serve as hydrogel scaffolds (Figure 3a). These matrices not only enhanced microbial adhesion but also initiated biomineralization processes [184].

#### 5.2.2 Microbial Regulation of Hydrogel Restoration and Reinforcement

In addition to their well‐established role in modulating hydrogel formation, living microorganisms can actively contribute to the structural restoration of hydrogel systems through continuous metabolic activity [185]. Within these living materials, embedded microbial cells remain viable and responsive, producing bioactive metabolites or structural components that restore the hydrogel network over time. This dual functionality—enabling both in situ synthesis and self‐directed restore—positions microbial systems as a powerful tool for constructing dynamic and sustainable hydrogel platforms. Such regenerative capacity not only prolongs the functional lifespan of hydrogel systems but also enables their sustained performance in complex biomedical environments where conventional materials often fail [186, 187]. A defining advantage of microorganism‐derived hydrogels lies in their ability to autonomously restore internal structure without external intervention, driven by continuous microbial activity. For example, the extracellular polysaccharide secreted by *Enterococcus sp. F2* could interact with gelatin matrix to form physical cross‐linking points, significantly enhancing the restoration ability of hydrogels [188]. This property has also been illustrated across diverse material contexts [189]. For instance, as demonstrated by Ehrlich et al. in 1996, microbial spores incorporated into concrete germinated and became metabolically active in response to environmental cues. Once reactivated, they catalyzed the precipitation of calcium carbonate, which gradually filled cracks and restored the structural integrity of the material (Figure 3b) [190]. This biomineralization‐driven restoration forms cohesive seals within the damaged region, substantially enhancing structural longevity. In dynamic physiological environments, where materials are subject to shear erosion, such as peristaltic motion in the gastrointestinal tract, which makes the capacity for continual self‐replenishment becomes essential. Living hydrogels, supported by sustained microbial metabolism, are capable of reconstructing their structural matrix to maintain integrity and therapeutic functionality [84, 191]. Beyond self‐regeneration and restoration, living microorganisms also contribute to the in situ mechanical strength enhancement of existing hydrogel networks [82]. The monomer precursor secreted by microorganisms can participate in secondary polymerization within the pre‐formed matrix, thereby enhancing its mechanical strength [189]. For example, microorganism‐induced calcium carbonate mineralization has been shown to increase the fracture toughness of sand‐gelatin hydrogels by approximately 1.5‐fold (Figure 3c) [186]. Furthermore, photosynthetically produced glucose, generated within embedded chloroplasts, could react with isocyanate‐functionalized polymers to establish additional crosslinking points. Yu et al. utilized spatially controlled light exposure to modulate glucose production, enabling localized and dynamic mechanical enhancement of synthetic hydrogel constructs [192]. Moreover, McCuskey et al. demonstrated that integrating the electrogenic microorganism *Shewanella oneidensis* MR‐1 with a conjugated polyelectrolyte enabled the formation of a living hydrogel with significantly enhanced mechanical properties. This enhancement was attributed to the interaction between extracellular polysaccharides secreted by the microorganism and the polymer matrix [193].

#### 5.2.3 Microbial Regulation of Hydrogel Degradation

Microorganisms can drive the degradation of hydrogel matrices through both mechanical disruption and enzymatic hydrolysis, fundamentally influencing their stability, lifetime, and biofunctionality. Mechanically, the proliferation and spatial expansion of microbial populations within confined hydrogel networks generate internal stresses that compromise structural integrity. This process can lead to microchamber rupture, matrix delamination, or localized buckling, particularly in soft or weakly crosslinked systems [161]. In parallel, microbial cells actively contribute to chemical degradation through the secretion of extracellular depolymerases and hydrolases that cleave long‐chain polymers into oligomeric or monomeric fragments, which can subsequently serve as carbon and energy sources [194]. For instance, hydrolytic enzymes secreted by *Enterobacteria* were particularly efficient in degrading lipid‐ and protein‐based polymers [195], whereas soil‐derived microorganisms like *Pseudomonas fluorescens* and *Bacillus subtilis* produced cellulases that readily hydrolyzed cellulose‐rich hydrogels (Figure 3d) [196].

While natural polymers (e.g., polysaccharides, proteins, polyesters) are readily susceptible to microbial degradation, synthetic polymers with stable carbon–carbon backbones (–CH_2–_CHR–) such as polyacrylamide (PAAm), polyvinyl alcohol (PVA), and polyacrylic acid (PAA) exhibit strong resistance to enzymatic and hydrolytic attack, rendering them largely non‐biodegradable under standard conditions [197]. Nevertheless, the extraordinary metabolic diversity of microorganisms provides a vast reservoir of potential polymer‐degrading enzymes and pathways. As early as 1975, *Pseudomonas aeruginosa* was shown to degrade polyethylene glycol (PEG) through a secreted extracellular enzyme capable of metabolizing both low‐ and high‐molecular‐weight PEG fractions [198, 199]. More recently, *Ideonella sakaiensis* has attracted considerable attention due to its secretion of polyester hydrolases that enabled efficient decomposition of polyethylene terephthalate (PET) plastics [200]. Compared to dry, non‐porous plastics, hydrogels offer a hydrated and high‐surface‐area environment that facilitates microbial adhesion, infiltration, and enzymatic accessibility. This structural advantage renders hydrogels inherently more prone to microbial colonization and controlled biodegradation. Consequently, microbial regulation of hydrogel degradation presents not only a challenge for long‐term stability but also a unique opportunity to engineer tunable, bioresponsive biodegradability for applications spanning wound healing, tissue regeneration, and sustainable materials design.

### 5.3 Integrative Synergy of Hydrogel Matrix and Microorganism

#### 5.3.1 Hydrogels Serving as Prebiotic Scaffolds

**FIGURE 4:**
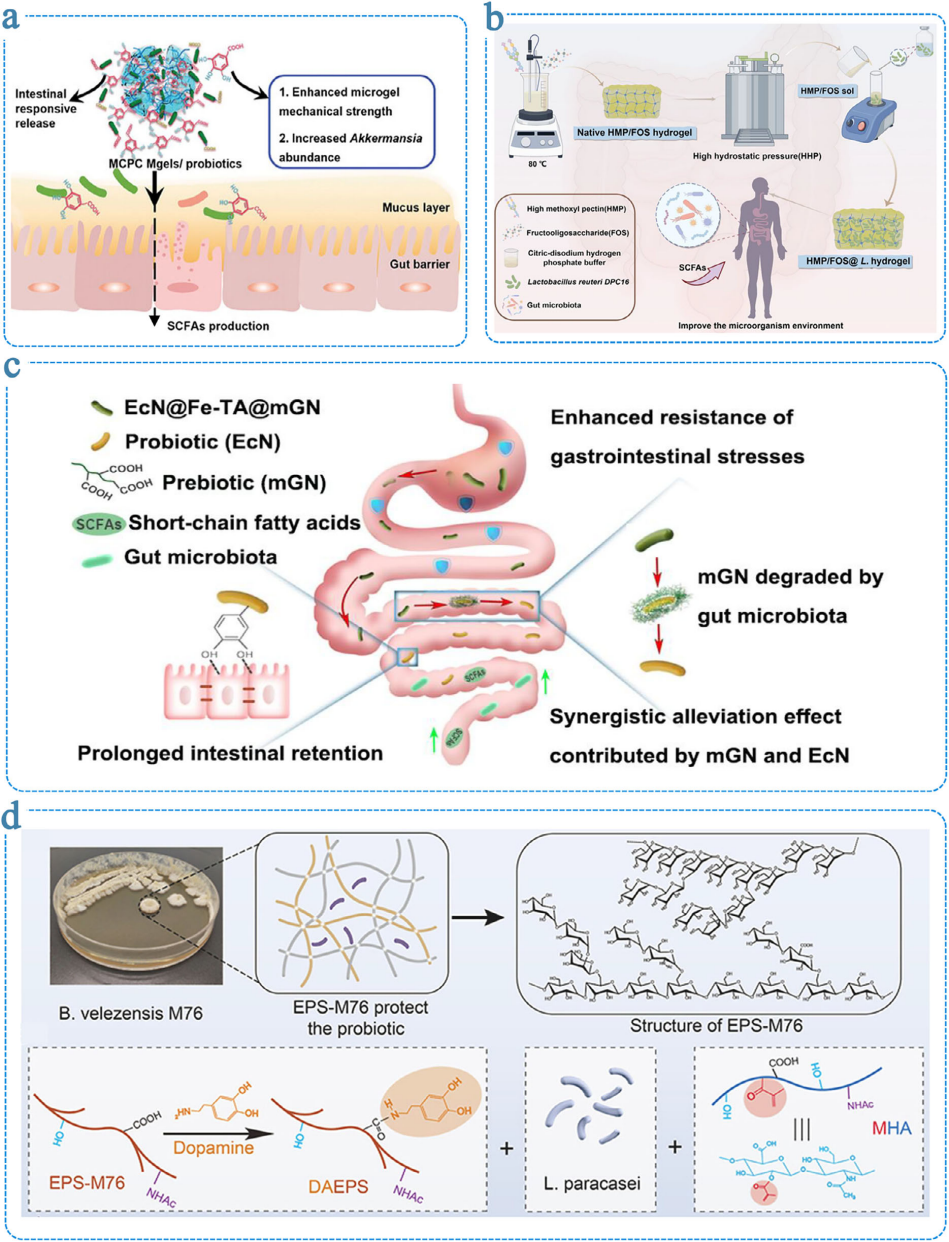
Hydrogels serving as prebiotic scaffolds. (a) Brown bioactive polyphenols in microgel enable increase the abundance of *Akkermansia* as precise prebiotics. Reproduced with permission [201]. Copyright 2024, John Wiley and Sons. (b) High‐methoxy pectin/fructooligosaccharides in living hydrogels act as prebiotics to promote the production of short‐chain fatty acids. Reproduced with permission [204]. Copyright 2025, Elsevier. (c) Modified prebiotic‐based “shield” encapsulated probiotics. Reproduced with permission [205]. Copyright 2023, American Chemical Society. (d) Highly active probiotic hydrogels based on EPS‐M76 with prebiotic activity. Reproduced with permission [24]. Copyright 2024, Elsevier.

As discussed in the previous section, microbial cells can facilitate the degradation of hydrogel matrices through both mechanical disruption and enzymatic hydrolysis. When the matrix itself possesses prebiotic functionality, this degradation transforms from a destructive process into a mutually beneficial metabolic cycle. In such systems, probiotics selectively metabolize the hydrogel polymers to generate bioactive metabolites—particularly short‐chain fatty acids (SCFAs)—that in turn promote microbial viability, metabolism, adhesion, and colonization (Figure 4). This establishes a self‐reinforcing, closed‐loop system, in which the hydrogel functions not only as a physical carrier and protective barrier but also as a nutritional substrate sustaining probiotic activity through controlled degradability and biochemical feedback. Meanwhile, microbial metabolism dynamically remodels the hydrogel's structure and functionality, leading to an adaptive and synergistic enhancement of the microbe–material interface.

Achieving such synergy depends critically on hydrogel composition, particularly the compatibility between microbial metabolic pathways and the molecular architecture of the polymer matrix. Natural polysaccharides, with their diverse structural motifs and selective biodegradability, are especially well‐suited for designing prebiotic hydrogels. For instance, Zhang et al. developed a ferric ion co‐crosslinked microgel of *Mesona chinensis* polysaccharides and mechanically reinforced by phenolic‐metal frameworks derived from naturally bound brown bioactives These polyphenolic components acted as specific prebiotics, selectively enriching *Akkermansia* species in vivo, thereby demonstrating targeted modulation of gut microbiota (Figure 4a) [201]. Similarly, a fucose‐rich mucilaginous polysaccharide hydrogel extracted from *Abroma augusta* serves as prebiotics to selectively supported the proliferation of beneficial *Lactobacillus* while inhibiting pathogenic growth—ensuring both safety and microbial specificity [202]. Building on this principle, inulin‐based prebiotic hydrogels have been designed to co‐encapsulate multiple probiotic strains, establishing synbiotic systems that combine structural encapsulation with nutritional support. Inulin served as a selectively metabolized substrate, sustaining probiotic viability during oral delivery and enhancing intestinal retention and colonization efficiency [203]. Likewise, a composite prebiotic hydrogel composed of high‐methoxyl pectin and oligofructose fabricated under high hydrostatic pressure encapsulated *Lactobacillus reuteri* DPC16, leading to enhanced SCFA production and improved intestinal health outcomes (Figure 4b) [204].

Beyond matrix metabolism, prebiotic components can also act as surface‐modifying agents to enhance probiotic protection and adhesion. For instance, Xie et al. constructed a “shielded” probiotic system in which modified prebiotics self‐assembled on probiotic surfaces via electrostatic attraction, chelation, and hydrogen bonding, forming a hydrogel‐based protective shell that improved survivability and mucosal adhesion (Figure 4c) [205]. In another example, Xu et al. encapsulated *Lactobacillus paracasei* TYM202 within a hydrogel matrix derived from the extracellular polysaccharide (EPS‐M76) of *Bacillus velezensis* M76T11B. The EPS‐M76 matrix not only acted as a biocompatible scaffold but also exhibited intrinsic prebiotic activity, sustaining the metabolic activity and proliferation of the encapsulated *Lactobacillus paracasei* TYM202 (Figure 4d) [24]. Overall, the biochemical specificity of hydrogel composition is pivotal for ensuring both efficacy and biosafety in prebiotic–probiotic hybrid systems. By matching polymer structure with microbial metabolism, these materials transcend the role of passive carriers to become metabolically interactive scaffolds, enabling adaptive, nutrient‐responsive living hydrogels with superior therapeutic potential.

#### 5.3.2 Hydrogels Working in Synergy With Microorganisms

Beyond serving as prebiotic substrates, many hydrogel matrices derived from natural polysaccharides or bioactive polymers possess intrinsic biological activities—such as anti‐inflammatory, antioxidant, and pro‐angiogenic effects—that complement microbial functions [206, 207]. These inherent bioactivities allow hydrogels to interact synergistically with encapsulated probiotics, promoting microbial stabilization and activation while simultaneously contributing to host tissue regeneration and immune modulation [208, 209]. Under pathological conditions, this dual‐functionality facilitates coordinated therapeutic responses, wherein both the hydrogel and the encapsulated microorganisms play active roles in promoting superior therapeutic outcomes.

**FIGURE 5:**
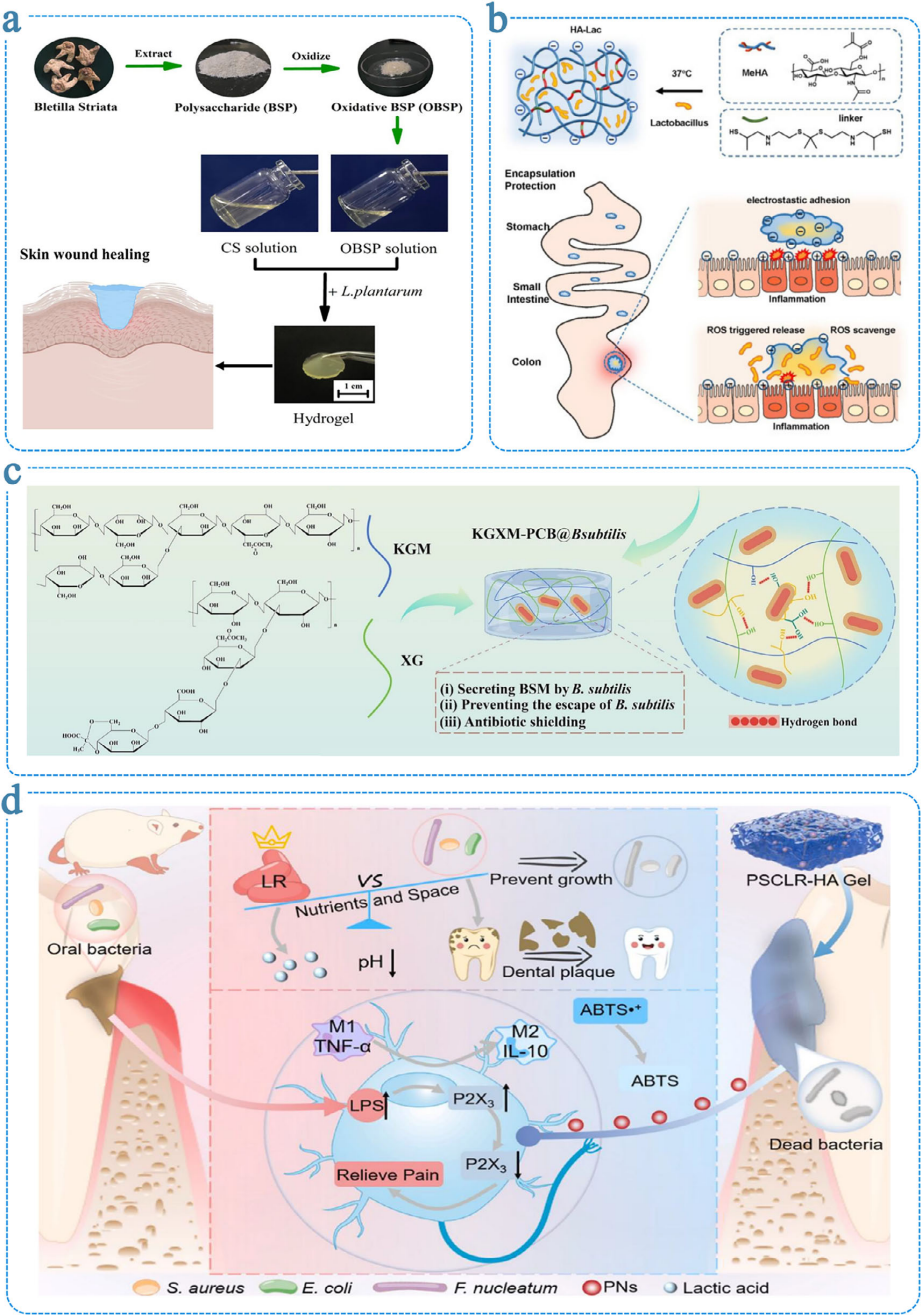
Hydrogels working in synergy with microorganisms. (a) Oxidized *Bletilla striata* polysaccharide and *Lactobacillus plantarum* in living hydrogels synergistically promote skin wound healing. Reproduced with permission [119]. Copyright 2020, Elsevier. (b) Thioketal in living hydrogel enable alleviate colitis by scavenging ROS in synergy with *Lactobacillus reuteri*. Reproduced with permission [31]. Copyright 2022, Elsevier. (c) Konjac glucomannan/xanthan gum and *Bacillus subtilis* in living hydrogel synergistically promote wound healing. Reproduced with permission [116]. Copyright 2025, Elsevier. (d) Puerarin and *Lactobacillus rhamnosus* in living hydrogel synergistically treat periodontitis. Reproduced with permission [32]. Copyright 2025, Elsevier.

This synergistic mechanism has been substantiated in several experimental studies. For instance, Yang et al. fabricated a living hydrogel by combining oxidized *Bletilla striata* polysaccharides—which possess strong antioxidant capacity—with chitosan, and encapsulated *Lactobacillus plantarum* within the composite network. The resulting living hydrogel exhibited enhanced probiotic viability and metabolic activity, along with a strong ability to regulate wound microenvironmental pH. Notably, although probiotic‐free hydrogels exhibited some therapeutic benefit, the presence of living probiotics led to markedly accelerated wound closure, enhanced collagen deposition, and improved tissue remodeling in full‐thickness skin‐defect models (Figure 5a) [119]. Similar synergistic outcomes were observed in systems based on methacrylated hyaluronic acid/thiolated thioketal hydrogels and konjac glucomannan/xanthan gum composites, where probiotic integration significantly accelerated re‐epithelialization and collagen remodeling compared with non‐living controls (Figure 5b,c) [31, 116]. In another example, Wang et al. constructed a chitosan/hyaluronic acid/puerarin hydrogel encapsulating *Lactobacillus rhamnosus*. The bioactive compound puerarin acted synergistically with the encapsulated probiotic to treat periodontitis in rat models, effectively disrupting pathogenic biofilms while promoting gingival tissue repair and demonstrating excellent biosafety (Figure 5d) [32].

## 6 Applications of Living Hydrogels in Disease Treatment

To elucidate the translational promise of living hydrogels, this section delves into their therapeutic deployment across diverse disease contexts (Table 3). In gastrointestinal disorders and wound healing, living hydrogels serve as protective scaffolds with controlled release capability, while the probiotics they encapsulate actively modulate microbial communities, suppress pathogenic colonization, and promote tissue repair. Beyond microbial regulation, these hybrid systems are increasingly explored in oncology, where engineered probiotic strains encapsulated within hydrogels secrete antibodies, cytokines, or other bioactive metabolites that remodel the tumor microenvironment, modulate immune checkpoints, and enhance antitumor efficacy. In addition, probiotic‐loaded hydrogels are being investigated in other pathological contexts, including hepatic injury, bone defects, periodontal inflammation, and mucosal infections, where their therapeutic efficacy largely arises from the synergistic regulation of local immune responses and metabolic pathways.

**TABLE 3:** Applications of living hydrogels in disease treatment.

Disease type	Hydrogel base	Therapeutic(s)	Treatment strategy	Model	Refs
Intestinal diseases treatment	Sodium alginate‐based pH‐responsive microspheres	TNF‐α‐modified *Escherichia coli Nissle* 1917	Probiotic colon targeted release	Murine colitis model	[213]
	Inulin/hyaluronic acid (HA)‐modified carbon dot nanoenzymes‐based pH‐responsive hydrogel	*Lactobacillus rhamnosus* GG	Probiotic colon targeted release	DSS‐induced colitis in mice	[27]
	1,4‐phenylenediboronic acid functionalized alginate/polyvinyl alcohol‐based ROS‐responsive hydrogel	*Lactobacillus plantarum*	Probiotic colon targeted release	Simulated gastrointestinal conditions	[25]
	Gelatin‐based ROS‐responsive microgel	*Bacillus subtilis* spores	Probiotic colon targeted release	DSS‐induced colitis in mice	[163]
	pH and ROS dual‐responsive alginate/ thioketal‐based hydrogel	*Lactobacillus rhamnose* GG	Probiotic colon targeted release	DSS‐induced colitis in mice	[215]
Wound healing	Fructooligosaccharide hydrogel	*Lactobacillus plantarum*	Diabetic wound healing	Diabetic rat wounds	[218]
	Gelatin‐based hydrogel crosslinked via Schiff‐base chemistry	*Lactobacillus plantarum*	Infected wound healing	Ex vivo human skin	[74]
	Hydrogel network by covalent cross‐linking of methacrylate‐modified hyaluronic acid	*Lactobacillus reuteri*	Infected wound healing	Infected mouse wounds	[29]
	Calcium alginate / fucoidan composite hydrogel	*Lactobacillus rhamnosus*	Ulcer healing in vivo	Oral ulcers in vivo	[219]
	Heparin‐poloxamer thermosensitive hydrogel	Genetically modified *Lactococcus lactis*	Diabetic wound healing	Diabetic mouse wounds	[222]
	PEGDA/chitosan hydrogel	CXCL12‐secreting engineered *Lactococcus lactis*	Skin defect healing	Rat skin defect	[223]
	Extracellular matrix hydrogel derived from pigskin	Photosynthetic bacteria	Infected wound healing	Infected mouse wounds	[224]
Cancer treatment	Gelatin‐inulin hydrogel	*Lactobacillus reuteri*	Immunotherapy	CT26 tumor‐bearing mice	[226]
	Chitosan / sodium alginate microgel	*Escherichia coli* Nissle 1917	Immunotherapy	CT26 tumor‐bearing mice	[229]
	Sodium alginate hydrogel	Firefly luciferase‐expressing *Salmonella typhimurium* and chlorin e6	Cancer photodynamic therapy and immunotherapy	B16 and CT26 tumor‐bearing mice	[230]
	Thiolated hyaluronic acid hydrogel	*Thiobacillus denitrificans*	Chemotherapy drug CPT therapy and eliminate H_2_S	CT26 tumor‐bearing mice	[26]
Liver injury treatment	Phenolic‐metal framework strengthened *Mesona Chinensis* polysaccharides microgels	*Akkermansia muciniphila*	Antioxidant, anti‐inflammatory, and gut microbiota regulation	APAP‐induced mouse liver injury	[201]
Skeletal repair	Hyaluronic acid methacryloyl hydrogel	*Escherichia coli* Nissle 1917	Enhanced neovascularization and full‐thickness bone healing	In vivo multiple bone defect	[23]
Osteomyelitis	Alginate hydrogel	Engineered *Bacillus subtilis*	Antibacterial, promote angiogenesis and osteogenic differentiation	Chronic osteomyelitis	[231]
Periodontal disease treatment	Light‐responsive hydrogel	*Lactobacillus rhamnosus* GG	Antibacterial, anti‐inflammatory and immunomodulatory	*Porphyromonas gingivalis* infected rat periodontitis	[232]
Gastric mucosal repair	Hyaluronic acid / tannic acid / polyvinyl alcohol hydrogel	*Lactobacillus reuteri*	Anti‐inflammatory, reduce *Helicobacter pylori* colonization and gut microbiota regulation	*Helicobacter pylori* infection of gastric mucosa	[30]
Iron deficiency anemia	Alginate/starch hydrogel	*Lactobacillus fermentum* and iron dextran	Iron deficiency anemia	Iron‐depleted mouse model	[233]

### 6.1 Living Hydrogels for Intestinal Diseases Treatment

The human gastrointestinal (GI) tract hosts a complex and dynamic microbial ecosystem essential for maintaining physiological homeostasis, regulating immune responses, and providing colonization resistance against pathogens [210]. Disruptions to this intricate host‐microbiota balance, commonly termed dysbiosis, are strongly associated with both the onset and progression of inflammatory bowel diseases (IBD), including ulcerative colitis and Crohn's disease. Among the therapeutic strategies explored to counteract dysbiosis, the administration of live probiotics has attracted substantial attention owing to its potential to restore microbial equilibrium, reinforce mucosal barrier function, and attenuate chronic inflammation [211]. However, the harsh gastrointestinal environment, characterized by low gastric pH, digestive enzymes, and bile salts, severely compromises probiotic survival and functional performance following oral administration, thereby limiting therapeutic efficacy [16]. To overcome these barriers, bio‐responsive living hydrogels have emerged as a next‐generation therapeutic platform. These materials not only provide a protective microenvironment for encapsulated microorganisms but also exhibit stimulus‐adaptive degradation and release behavior, allowing synchronization with pathological cues in the inflamed intestine for precise and effective probiotic delivery. Upon targeted release, the delivered probiotics dynamically reconfigure the intestinal microbiota by favoring the colonization of beneficial commensals and suppressing opportunistic pathogens. This ecological rebalancing exerts downstream effects on host physiology by mitigating intestinal immune dysregulation and promoting local tissue repair, ultimately contributing to disease alleviation in microbiota‐associated intestinal disorders.

**FIGURE 6:**
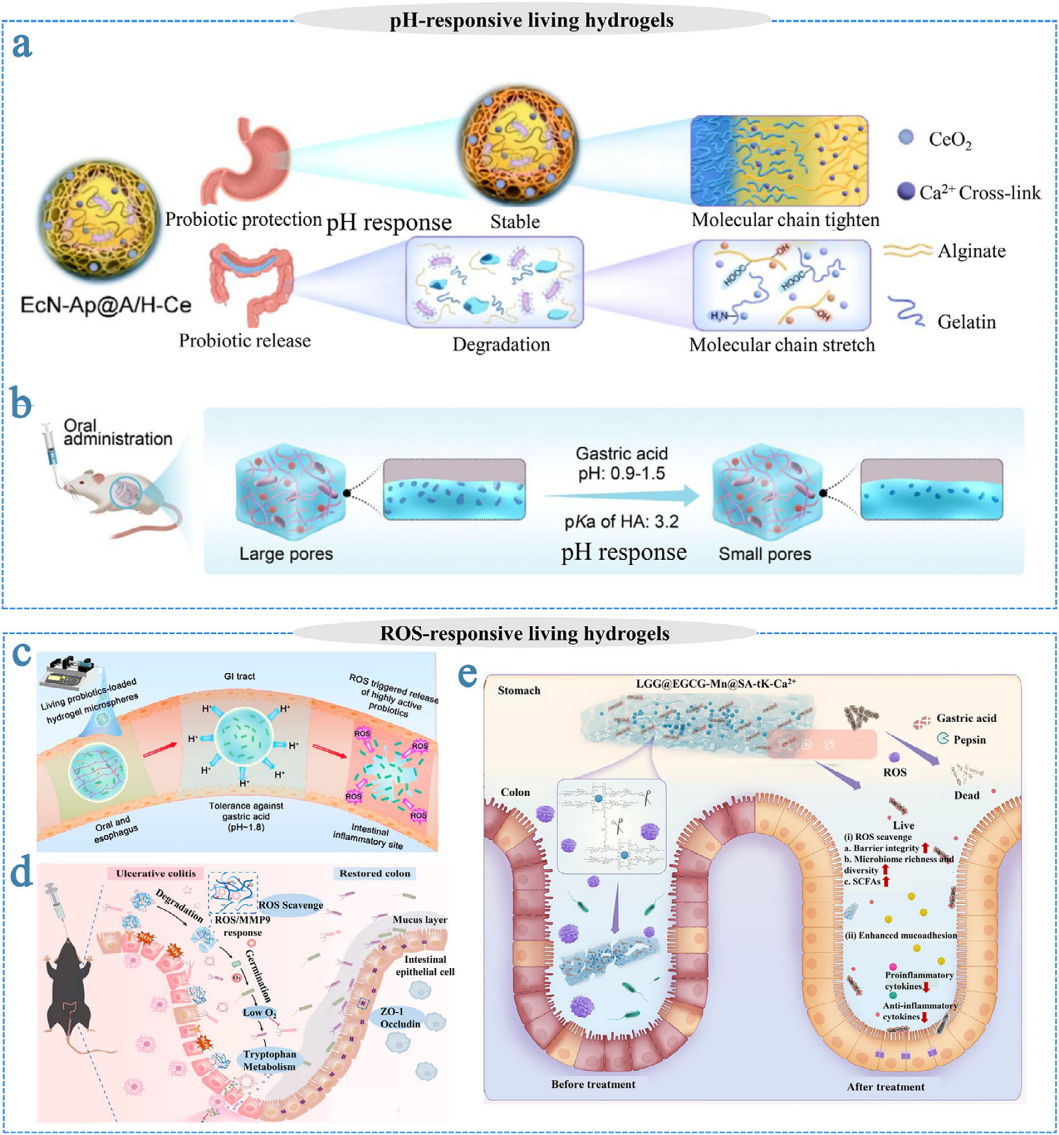
Living hydrogels for intestinal diseases treatment. (a) pH‐responsive characteristics of living hydrogel and its effective treatment for colitis. Reproduced with permission [213]. Copyright 2025, Elsevier. (b) Living hydrogel gastric acid‐responsive pore‐shrinking mechanism. Reproduced with permission [27]. Copyright 2025, ACS Nano. (c) ROS‐responsive characteristics of living hydrogel microsphere. Reproduced with permission [25]. Copyright 2022, ACS Applied Polymer Materials. (d) ROS and MMP‐9 responsive supramolecular liposome‐microgel complex for treating colitis. Reproduced with permission [163]. Copyright 2025, Elsevier. (e) ROS‐responsive characteristics of living hydrogel and its effective treatment for colitis. Reproduced with permission [215]. Copyright 2025, ACS Applied Polymer Materials.

pH*‐Responsive Living Hydrogels*. The gastrointestinal pH gradient provides a natural physiological cue for the design of pH‐responsive delivery systems capable of achieving spatiotemporally controlled release of living microorganisms. pH‐responsive hydrogels exploit this variation by incorporating ionizable functional groups such as carboxyl, amino, or phenolic hydroxyl moieties within their polymer backbone. These structures remain contracted under acidic gastric conditions, thereby protecting encapsulated probiotics during transit through the stomach, and subsequently swell or degrade in the neutral to alkaline environment of the intestine, triggering site‐specific release [212]. This design principle has been successfully applied in several advanced hydrogel platforms. For instance, Tang et al. developed a microfluidic sodium alginate microsphere system encapsulating *Escherichia coli* Nissle 1917 conjugated with TNF‐α aptamers. This living hydrogel effectively alleviated ulcerative colitis by reducing colonic inflammation, restoring epithelial integrity, and rebalancing gut flora (Figure 6a) [213]. In another study, Zhang et al. developed a multifunctional inulin‐based hydrogel incorporating living probiotics and a hyaluronic acid‐functionalized carbon‐dot nanoenzyme. The hydrogel exhibited pH‐dependent pore contraction that protected probiotics in the stomach while providing ROS‐scavenging and anti‐inflammatory activity in the intestine, yielding significant therapeutic efficacy against colitis (Figure 6b) [27].

*ROS‐Responsive Living Hydrogels*. Besides pH fluctuations, oxidative stress defining hallmark of the inflamed intestinal microenvironment in IBD. Elevated levels of ROS, such as hydrogen peroxide and superoxide, are strongly associated with epithelial damage, immune cell infiltration, and microbial dysbiosis. To counter this, ROS‐responsive living hydrogels are designed to selectively degrade or undergo conformational changes in response to such elevated oxidative signals, enabling on‐demand probiotic release specifically at inflamed sites. These systems typically incorporate ROS‐labile linkages, including disulfide, diselenide, or thioether bonds, into the hydrogel network to achieve environmentally triggered degradation [214]. For example, alginate‐polyvinyl alcohol hydrogels functionalized with 1,4‐phenylenediboronic acid underwent selective degradation in ROS‐rich environments, leading to the controlled release of *Lactobacillus plantarum* directly within inflamed intestinal tissues. The released bacteria retained high metabolic activity and were capable of modulating the gut microbiota, suppressing pathogen proliferation, and alleviating inflammation (Figure 6c) [25]. Moreover, a supramolecular liposome‐microgel complex incorporating *Bacillus subtilis* spores, prepared through diselenide‐functionalized gelatin and microfluidic engineering, exhibited selective degradation in the presence of ROS and matrix metalloproteinase 9, enabling precise microbial colonization at inflamed mucosal sites. It further reprogrammed intestinal immunity by inhibiting TLR4 signaling and modulating tryptophan metabolism, significantly enhancing probiotic therapy for ulcerative colitis (Figure 6d) [163]. Expanding this approach, Tang et al. further developed a dual pH/ROS‐responsive hydrogel, in which *Lactobacillus rhamnosus* (LGG) modified with EGCG–Mn nanoparticles (LGG@EGCG‐Mn) was encapsulated in an alginate/ thioketal matrix. This system dynamically responded to both acidic and oxidative stimuli, reducing colonic inflammation, restoring microbial homeostasis, and promoting short‐chain fatty acid production, thereby achieving potent prevention and treatment of ulcerative colitis (Figure 6e) [215]. Despite these advances, achieving precise control of degradation kinetics and release behavior remains challenging. The heterogeneous spatial distribution of ROS in inflamed tissues complicates response calibration, raising the risk of premature disassembly or incomplete release. Moreover, the absence of clearly defined activation thresholds limits the predictability and reproducibility of hydrogel responsiveness, while long‐term stability under fluctuating oxidative conditions remains insufficiently characterized. Future progress will rely on the rational design of responsive motifs with tunable sensitivity to physiological cues, optimization of crosslinking architectures for modular degradation, and integration of feedback‐regulated release circuits that couple microbial metabolic activity with environmental stimuli. Such strategies will be essential to translate ROS‐responsive living hydrogels into clinically viable, precision‐engineered therapeutic systems for IBD and other intestinal disorders.

### 6.2 Living Hydrogels for Wound Healing

**FIGURE 7:**
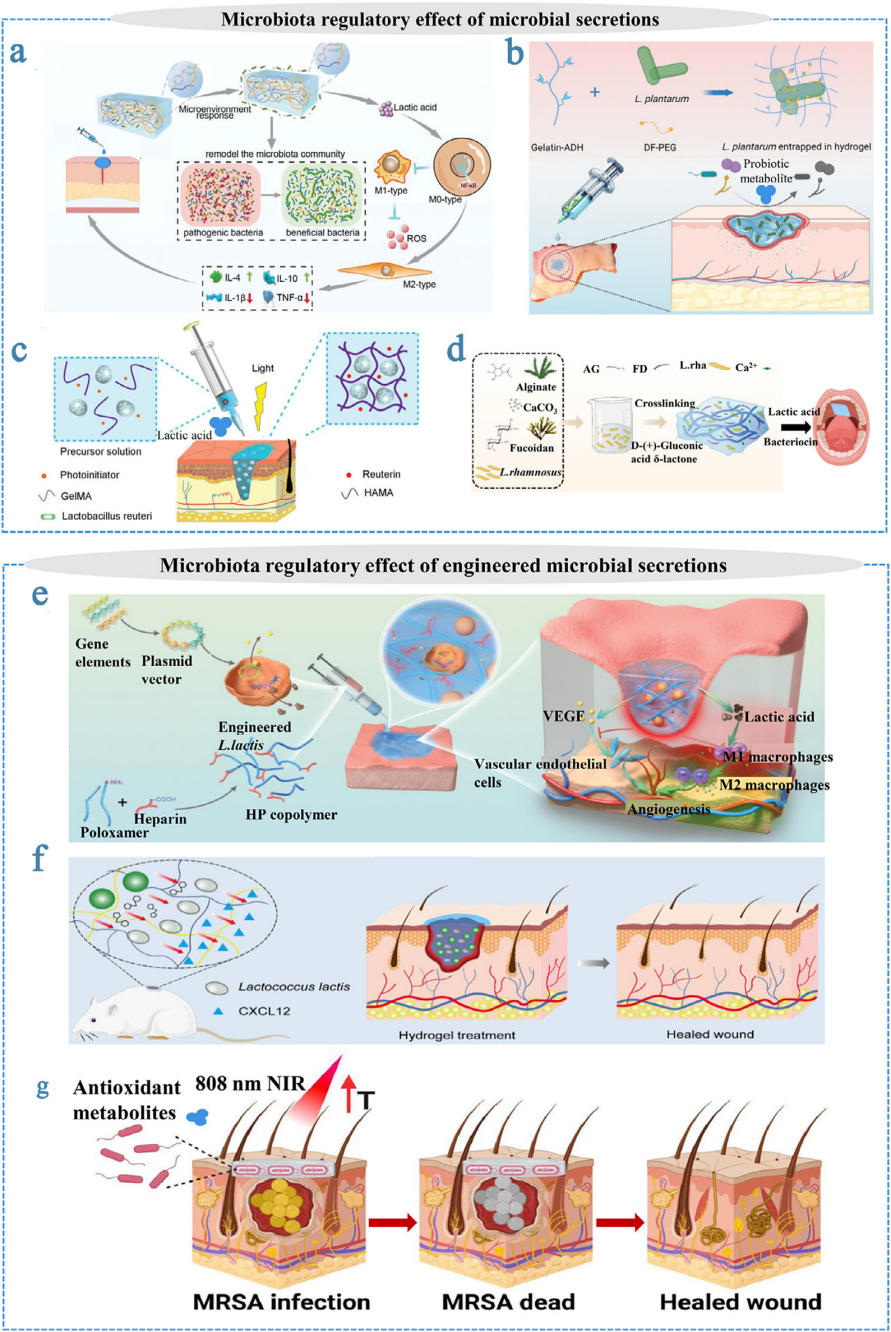
Living hydrogels for wound healing. (a) Schematic diagram of living microecological hydrogel encapsulating probiotics secreting lactic acid to promote wound healing in diabetes. Reproduced with permission [218]. Copyright 2024, John Wiley and Sons. (b) The probiotics in the living hydrogel secrete antibacterial agents to the wound site to inhibit the growth of prevalent wound pathogens. Reproduced with permission [74]. Copyright 2024, John Wiley and Sons. (c) Schematic diagram of the process of lactic acid secreted by probiotics in living hydrogel to accelerate wound healing. Reproduced with permission [29]. Copyright 2021, John Wiley and Sons. (d) *Lactobacillus rhamnosus* secretes lactic acid and bacteriocin in living hydrogels to inhibit the proliferation of oral pathogenic bacteria. Reproduced with permission [219]. Copyright 2023, Elsevier. (e) Engineering bacteria activated a multifunctional hydrogel for wound repair and regeneration in diabetes. Reproduced with permission [222]. Copyright 2021, John Wiley and Sons. (f) Hydrogel‐encapsulated engineered microbial consortium as a “living material” for promoting skin wound healing. Reproduced with permission [223]. Copyright 2023, American Chemical Society. (g) Photosynthetic bacteria in living hydrogels secrete antioxidant metabolites to promote wound healing. Reproduced with permission [224]. Copyright 2022, Elsevier.

*Microbiota Regulatory Effect of Microbial Secretions*. Pathogenic bacteria are now recognized as key disruptors in the wound healing process, particularly in chronic or immunocompromised conditions. Upon injury, opportunistic bacteria such as *Staphylococcus aureus* and *Pseudomonas aeruginosa* often invade the wound microenvironment, where they form biofilms, secrete virulence factors, and provoke prolonged inflammation. These actions not only delay epithelial regeneration but also degrade extracellular matrix components, ultimately impairing tissue remodeling and immune resolution [216]. Conventional antimicrobial wound dressings, while effective in eradicating pathogens, often disrupt commensal microbiota, delaying healing and impairing tissue homeostasis [217]. In contrast, living hydrogels restore microbial balance by leveraging the metabolic activity of encapsulated probiotics. Their therapeutic advantage stems from the metabolic activity of encapsulated probiotics, whose bioactive metabolites, particularly lactic acid and bacteriocins, establish an acidic microenvironment that disrupts harmful bacterial membrane integrity without inducing resistance. For instance, a microecological hydrogel containing *Lactobacillus plantarum* and fructooligosaccharides could promote rapid healing of diabetic wounds by modulating the skin microbiota and restoring immune homeostasis. This effect was driven by probiotic‐derived lactic acid, which induced macrophage polarization from the pro‐inflammatory M1 to the reparative M2 phenotype (Figure 7a) [218]. Similarly, Tao et al. prepared a living hydrogel by incorporating the probiotic *Lactobacillus plantarum*, which enabled localized release of probiotic metabolites, effectively eliminating bacterial and fungal pathogens while preventing biofilm formation—thereby accelerating wound closure (Figure 7b) [74]. Furthermore, Ming et al. further advanced this concept through a multilayer encapsulation strategy, embedding *Lactobacillus reuteri* within methacrylic acid–gelatin microspheres, which were then incorporated into a methacrylic acid–hyaluronic acid hydrogel. This hierarchical system achieved broad‐spectrum antibacterial effects by restricting microbial diffusion while promoting sustained secretion of lactic acid and bacteriocins, significantly enhancing the healing of infected wounds (Figure 7c) [29]. In the oral environment, Dou et al. designed a calcium alginate/fucoidan composite hydrogel patch loaded with *Lactobacillus rhamnosus*. The probiotics secreted lactic acid and bacteriocins that effectively suppressed oral pathogens, promoting mucosal healing in ulcer models (Figure 7d) [219].

*Microbiota Regulatory Effect of Engineered Microbial Secretions*. Advances in synthetic biology have enabled the engineering of probiotics with programmable secretion capabilities, expanding the therapeutic scope of living hydrogels [220]. While native probiotics secrete metabolites like lactic acid and bacteriocins, their activity is often limited by environmental variability. Through genetic circuit engineering—including synthetic promoters, inducible systems, and feedback control—engineered microbes can achieve precise spatial and temporal control of therapeutic protein expression, yielding more stable and predictable outcomes [221]. For example, Lu et al. engineered *Lactococcus lactis* express vascular endothelial growth factor (VEGF) under inducible control (strain LL_VEGF) and encapsulated it within a heparin–poloxamer thermosensitive hydrogel. Upon activation, LL_VEGF enhanced angiogenesis at the wound site while lactic acid secretion alleviated inflammation, collectively promoting tissue regeneration (Figure 7e) [222]. Similarly, developed a symbiotic living hydrogel co‐encapsulating engineered *Synechococcus elongatus* PCC7942 (strain cscB) and *Lactococcus lactis*. The engineered photosynthetic cyanobacterium fixed CO_2_ and secreted sucrose as a carbon source, sustaining *Lactococcus lactis*, which in turn secreted the chemokine CXCL12 to recruit macrophages and promote M2 polarization. This coordinated metabolic interplay modulated the wound immune microenvironment and accelerated healing (Figure 7f) [223]. Furthermore, Zhao et al. developed a living hydrogel dressing by encapsulating engineered photosynthetic bacteria within an extracellular matrix hydrogel derived from porcine skin. Under near‐infrared light irradiation, the photosynthetic bacteria efficiently converted light energy into heat, leading to a rapid increase in local temperature that effectively eradicated methicillin‐resistant *Staphylococcus aureus*. In addition to photothermal antibacterial activity, the engineered bacteria continuously secreted antioxidant metabolites that mitigated local inflammation and promoted tissue regeneration, thereby supporting the wound healing process (Figure 7g) [224]. These examples highlight a paradigm shift from passive probiotic delivery toward programmable bioactive platforms capable of dynamically orchestrating immune modulation, angiogenesis, and tissue repair.

Nevertheless, while living hydrogels offer promising therapeutic outcomes for infected or chronic wounds, their clinical translation necessitates careful biosafety consideration. One important concern involves the potential translocation of encapsulated microorganisms into surrounding tissues or the bloodstream during hydrogel degradation, especially in immunocompromised patients or highly vascularized wound beds. If not tightly regulated, microbial leakage may result in local infection or systemic dissemination, undermining both therapeutic efficacy and patient safety. Strategies such as multilayer hydrogel structures, microbial auxotrophy, or stimuli‐responsive degradation mechanisms have been proposed to mitigate this risk and improve containment reliability. Future studies must rigorously evaluate microbial biodistribution, immune clearance, and long‐term host–microbe interactions in clinically relevant wound models to ensure safety before human application.

### 6.3 Living Hydrogels for Cancer Treatment

**FIGURE 8:**
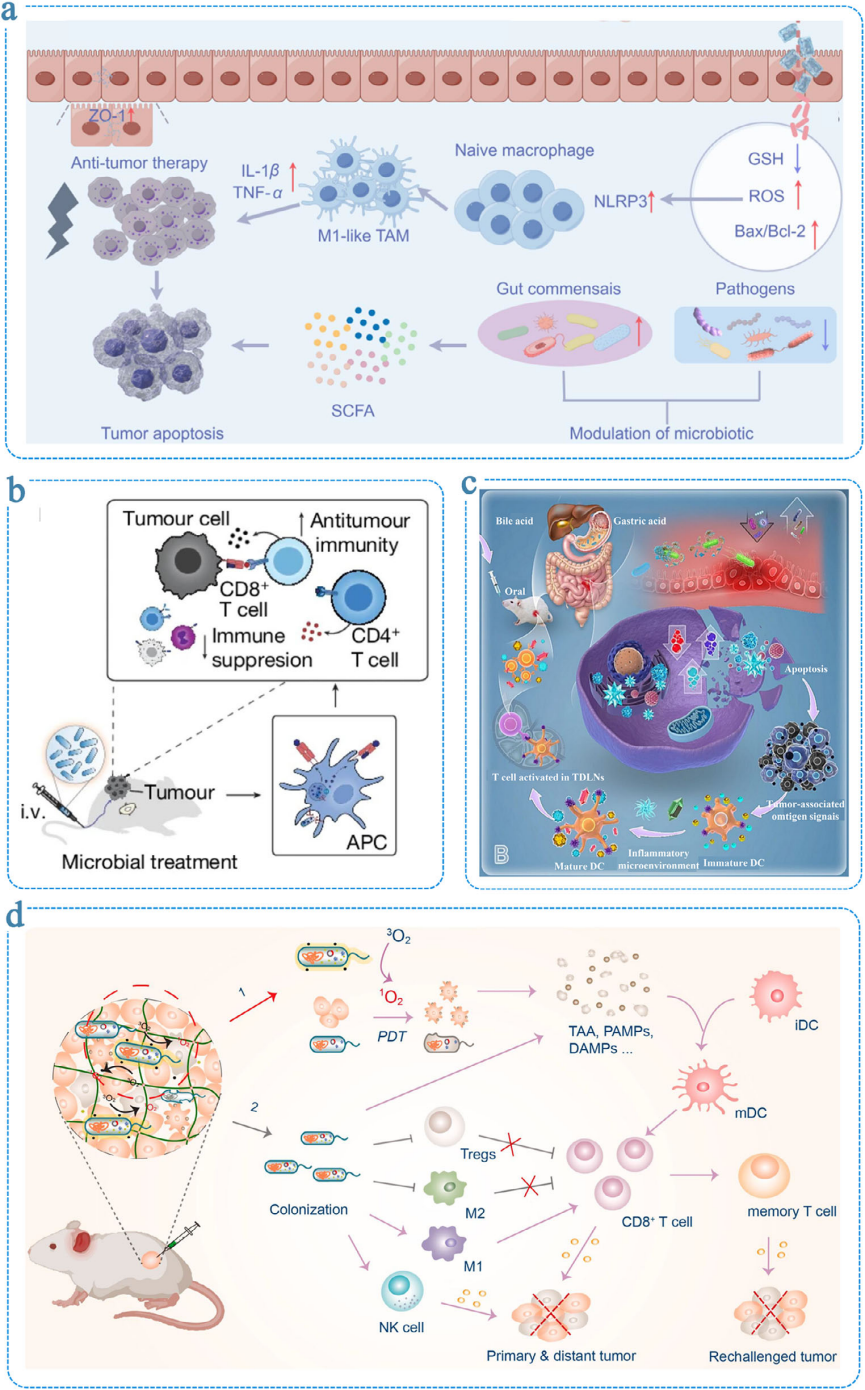
Engineered living hydrogels platforms for cancer therapy. (a) The mechanism of live hydrogels against colon cancer. Reproduced with permission [226]. Copyright 2025, Elsevier. (b) The mechanism of microbial tumor neoantigen vaccines in treating cancer. Reproduced with permission [228]. Copyright 2024, Springer nature. (c) EcN@(CS‐SA)_2_ microgel improves the immunotherapy effect of galunisertib. Reproduced with permission [229]. Copyright 2024, Elsevier. d) Scheme illustrating the engineering of bioluminescent bacteria for synergistic cancer treatment. Reproduced with permission [230]. Copyright 2022, Elsevier.

Bacteriotherapy offers distinct advantages over conventional cancer treatments, including tumor‐targeted motility under hypoxic conditions, deep intratumoral penetration, and activation of host antitumor immunity [225]. Recently, combining probiotic bacteria with hydrogel matrices has enabled localized, sustained, and immune‐responsive tumor therapy. For instance, Wang et al. formulated synbiotic hydrogel capsules by encapsulating *Lactobacillus reuteri* within an inulin–gelatin hydrogel crosslinked through hydrogen bonding. This system demonstrated anti‐tumor effects by depleting glutathione to release reactive oxygen species, accompanied by the activation of NLRP3, and the induction M1 macrophage polarization (Figure 8a) [226]. However, a persistent challenge lies in engineering microbial agents with high therapeutic potency and minimal off‐target toxicity. Targeting this, genetically modifications commensal species have shown promise in both tumor suppression and prolongation of viability in preclinical models [227]. For instance, Redenti et al. engineered probiotic *Escherichia coli* Nissle 1917 as an antitumor vaccination platform optimized for enhanced production and cytosolic delivery of neoepitope‐containing peptide arrays, with increased susceptibility to blood clearance and phagocytosis. This platform implemented a system that drove effective and specific T cell‐mediated anti‐cancer immunity, which could effectively control or eliminate tumor growth and prolong the survival of advanced mouse primary and metastatic solid tumors (Figure 8b) [228]. Nevertheless, growing evidence suggests that microorganism alone, even when genetically engineered, are unlikely to achieve full tumor eradication. Hence, engineered multimodal therapies, especially those integrating immune modulation, are being increasingly investigated for their curative potential. For example, a dual‐layer chitosan‐sodium alginate hydrogel (EcN@(CS‐SA)_2_) encapsulating *Escherichia coli* Nissle 1917 was orally administered with the TGF‐β inhibitor galunisertib. The hydrogel protected probiotics from gastric degradation and reshaped gut microbiota, while the combined therapy induced immunogenic cell death, enhanced dendritic‐cell maturation, and promoted CD8^+^ T‐cell infiltration. This synergistic treatment significantly reduced colorectal tumor volume and mitigated drug resistance, all while maintaining systemic biosafety (Figure 8c) [229]. Similarly, bioluminescent *Salmonella typhimurium* encapsulated within alginate hydrogels was combined with chlorin e6‐mediated photodynamic therapy, achieving enhanced antitumor efficacy and providing a generalizable strategy for treating light‐accessible tumors (Figure 8d) [230]. In another design, a thiolated hyaluronic acid hydrogel was designed to co‐encapsulate *Thiobacillus denitrificans* and camptothecin (CPT) for targeted oral delivery. By efficiently scavenging excessive hydrogen sulfide in the tumor microenvironment, a metabolite associated with angiogenesis and drug resistance, this formulation normalized tumor vasculature, improved CPT accumulation, and significantly suppressed tumor growth without notable toxicity [26]. In summary, hydrogel‐based living microbial systems represent a next‐generation paradigm in cancer therapy. Acting simultaneously as protective carriers and dynamic bioreactors, they enable localized microbial proliferation, stimuli‐responsive release of therapeutic metabolites, tumor‐microenvironment remodeling, and synergistic immune activation. By integrating synthetic biology, biomaterials engineering, and immunotherapy, living hydrogels hold substantial promise as safe, tunable, and multimodal platforms for precision cancer treatment.

### 6.4 Living Hydrogels for Other Diseases Treatment

Beyond gastrointestinal and oncological applications, living hydrogels have emerged as a versatile and translational therapeutic platform with efficacy demonstrated in hepatic injury, bone regeneration, periodontal inflammation, and mucosal infections.

**FIGURE 9:**
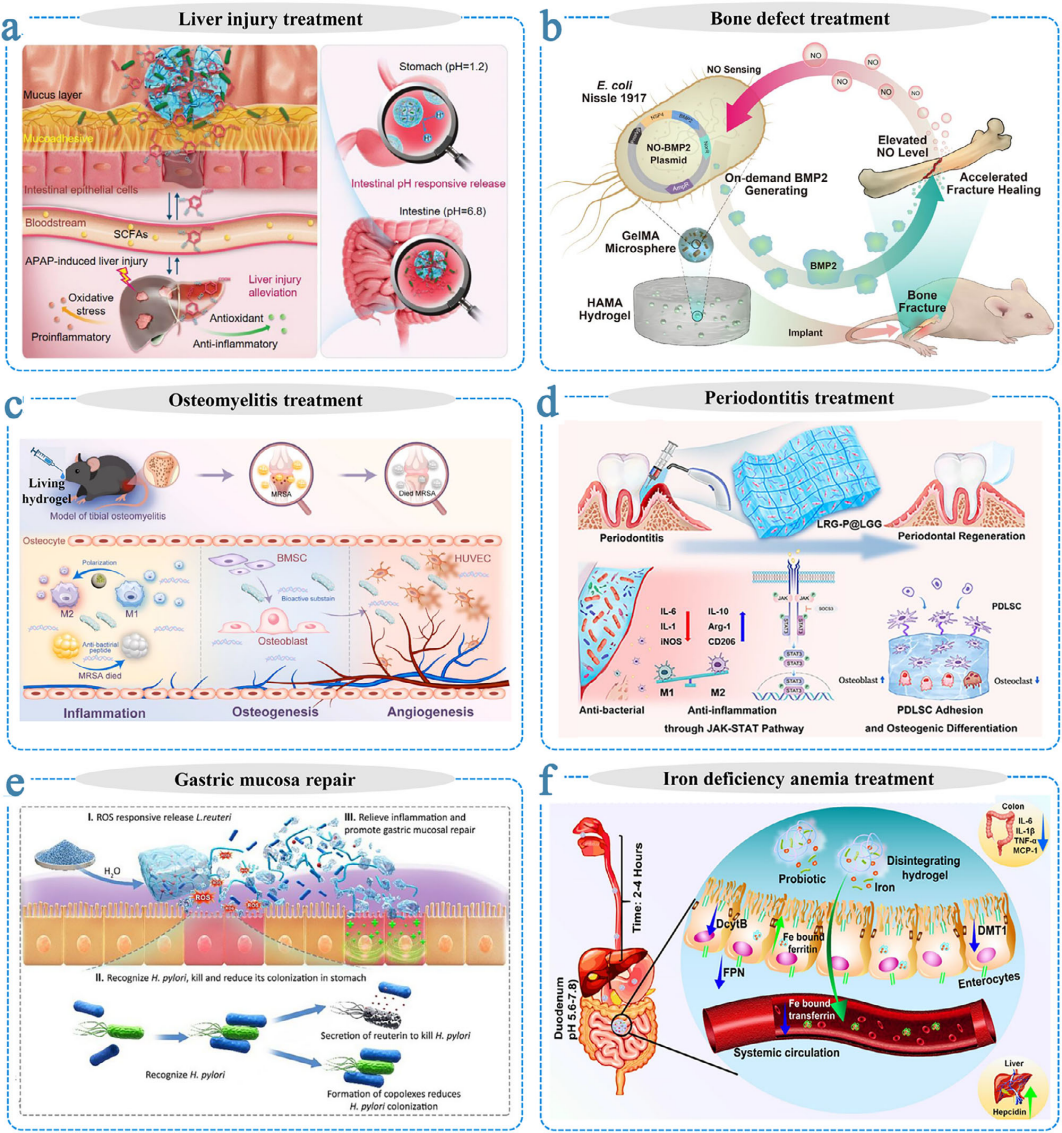
Living materials‐based carriers for other diseases therapy. (a) Phenolic‐metal framework strengthened *Mesona Chinensis* polysaccharides to alleviate the liver injury. Reproduced with permission [201]. Copyright 2024, John Wiley and Sons. (b) Living hydrogels promote bone defect healing. Reproduced with permission [23]. Copyright 2025, Elsevier. (c) Schematic illustration of osteomyelitis treatment of the engineered probiotic hydrogel. Reproduced with permission [231]. Copyright 2025, Elsevier. (d) Schematic illustration of the periodontitis therapy of the living hydrogel. Reproduced with permission [232]. Copyright 2025, Trends in Biotechnology. (e) *L. reuteri*@HTP hydrogel alleviate inflammation and support gastric mucosa repair. Reproduced with permission [30]. Copyright 2025, John Wiley and Sons. (f) Schematic diagram of the treatment of iron deficiency anemia with living hydrogel. Reproduced with permission [233]. Copyright 2021, ACS Applied Bio Materials.

For instance, to improve hepatic injury, a microgel system composed of herbal polysaccharides and polyphenol‐Fe^3^^+^ networks conferred resistance to gastric acid and enabled pH responsive release of *Akkermansia muciniphila*. The system maintained long‐term colonic colonization (>21 days) and significantly improved acetaminophen induced liver toxicity by restoring SCFAs levels and regulating gut‐liver axis inflammation (Figure 9a) [201]. For skeletal repair, NO‐responsive double‐layered hydrogels embedding engineered *Escherichia coli* Nissle 1917 facilitated inducible local expression of bone morphogenetic protein‐2 (BMP 2), thereby promoting osteogenesis and supporting neovascularization in both calvarial and femoral defect models (Figure 9b) [23]. In the treatment of osteomyelitis, an alginate hydrogel encapsulating engineered *Bacillus subtilis* exhibited potent antibacterial activity against *Staphylococcus aureus* and helped prevent the emergence of bacterial resistance. In addition, the hydrogel effectively induced M2 macrophage polarization and promoted angiogenesis, thereby enhancing the osteogenic differentiation of bone marrow mesenchymal stem cells and ultimately facilitating bone regeneration (Figure 9c) [231]. In periodontitis therapy, a photo‐crosslinked 4‐arm PEG dimethacrylate hydrogel loaded with *Lactobacillus rhamnosus* GG exhibited strong antibiofilm efficacy against *Porphyromonas gingivalis*. Beyond its antimicrobial function, the system modulated macrophage polarization through the SOCS3/JAK STAT signaling pathway, thereby normalizing the dysregulated periodontal microenvironment (Figure 9d) [232]. For mucosal infections such as gastric and vaginal diseases, responsive hydrogel systems have demonstrated both microbiota regulatory and immunomodulatory properties. A hydrogel composed of *Lactobacillus reuteri*, hyaluronic acid, tannic acid and polyvinyl alcohol could enhance the survival rate of *Lactobacillus reuteri* in adverse gastric environment and ensure selective release at *Helicobacter pylori*‐infected inflammatory sites. *Lactobacillus reuteri* targeted and reduced *Helicobacter pylori* colonization while secreting reuterin to eliminate the bacteria (Figure 9e) [30]. Furthermore, Sagar et al. developed an alginate/starch hydrogel that co‐encapsulated probiotics together with iron supplements and anti‐inflammatory agents for oral administration. This multifunctional formulation provided simultaneous iron supplementation, modulation of gut microbiota, and attenuation of local inflammation, thereby enhancing the therapeutic efficacy of oral treatment for iron deficiency anemia (Figure 9f) [233].

Collectively, these studies underscore the therapeutic breadth and versatility of living hydrogels across diverse pathological contexts. By integrating the structural tunability of advanced biomaterials with the adaptive metabolism of living microorganisms, these systems represent a new class of dynamic, self‐regulating, and clinically translatable therapeutics with broad potential for next‐generation regenerative and anti‐infective medicine.

## 7 Challenges and Future of Living Hydrogels

Living hydrogels represent a transformative class of therapeutic biomaterials that integrate the dynamic functionality of living microbes with the tunable physicochemical properties of polymeric networks. These systems offer unique advantages for the localized and sustained delivery of probiotics in diverse disease contexts, ranging from inflammation to tissue regeneration and metabolic modulation. Despite rapid advances in material chemistry, fabrication techniques, and microbial engineering, several fundamental and translational challenges continue to hinder the full clinical realization of these hybrid platforms. These include insufficient mechanistic understanding of in vivo activity, limited maintenance of microbial functions, challenges in multi‐species coordination, concerns over biocompatibility and immunogenicity, and regulatory uncertainty in therapeutic development. Addressing these issues requires a multidisciplinary strategy that bridges materials science, synthetic biology, immunology, and regulatory science to unlock the full therapeutic potential of living hydrogels.

### 7.1 Exploration of the Mechanism Underlying Disease Treatment

Living microorganism‐encapsulating hydrogels have emerged as promising therapeutic platforms, leveraging both the unique physicochemical properties of the hydrogel matrix and the intrinsic biofunctionality of the living microorganism. Through this synergy, they have demonstrated promising efficacy across multiple disease contexts. However, despite substantial advances in material design—spanning biocompatibility optimization, mechanical tuning, and stimuli‐responsive release—the fundamental mechanisms governing their therapeutic actions remain poorly understood, limiting rational design and clinical translation.

Current mechanistic understanding predominantly relies on canonical microbial functions such as antimicrobial metabolite secretion, competitive pathogen elimination, and immunomodulation. However, these mechanisms are largely characterized in simplified in vitro systems and do not adequately reflect the complex physiological conditions encountered in vivo, particularly in disease contexts such as chronic wounds, inflammatory disorders, or metabolic syndromes. For example, although organic acids and bacteriocins exhibit potent antimicrobial activity in vitro, their stability and functionality in fluctuating pH, oxygen gradients, and inflammatory microenvironments remain uncertain. In addition, the probiotic strains encapsulated in the hydrogel matrix may face numerous challenges during their transit through the body, such as encountering varying pH levels, immune responses, or the extracellular matrix components. These factors may affect their viability, colonization, and functional efficacy at the target site, yet systematic studies addressing these dynamics are scarce. Additionally, the interaction between living hydrogels and the host immune system represents another key but underexplored dimension. Although living microorganisms are known to modulate immune responses through cytokine regulation and immune cell activation, the specific pathways through which they influence macrophages, dendritic cells, or other immune subsets in vivo remain poorly defined. Moreover, it also remains unclear how different microbial strains induce distinct immune responses or how disease‐induced inflammation and microbial dysbiosis shape these interactions. Importantly, the in vivo temporal and spatial dynamics of immune responses to microorganism‐encapsulated hydrogels are rarely studied, leaving critical mechanistic gaps unanswered.

In summary, although living hydrogels hold potential for treating a wide spectrum of diseases, their mechanisms of action are complex and insufficiently understood. The interaction between the hydrogel matrix, living microorganism, and the host microenvironment is complex and multifactorial, requiring the integration of various analytical techniques, including multi‐omics, real‐time imaging, and animal models, to fully elucidate their therapeutic potential. Future research should focus on elucidating the dynamic crosstalk among microbial metabolism, immune regulation, and tissue repair processes, thereby providing the mechanistic foundation for the rational design and precision engineering of next‐generation living hydrogels.

### 7.2 Effective Maintenance of Microbial Functions Within Living Hydrogels

Despite the growing success of living hydrogels as delivery platforms for therapeutic microorganisms, multiple technical challenges continue to impede their functional optimization. A primary limitation lies in the preservation of microbial viability and activity throughout the entire life cycle of the material—from fabrication and storage to in vivo deployment. During encapsulation, microorganisms are often exposed to harsh physicochemical stresses, including acidic pH, bile salts, oxygen exposure, and mechanical shear, which can markedly reduce survival rates and metabolic functionality [234]. Similarly, during gastrointestinal transit or implantation, encapsulated microorganisms face additional challenges such as enzymatic degradation and immune clearance, leading to incomplete colonization and diminished therapeutic efficacy.

Another critical challenge involves the lack of precise control over microbial spatial distribution and release kinetics within the hydrogel matrix. Nonuniform dispersion and premature diffusion frequently result in uneven colonization or early depletion of active microbial populations at target sites [235]. Moreover, conventional hydrogel matrices may lack sufficient physical shielding and buffering capacity, rendering them unable to maintain a stable microenvironment conducive to long‐term microbial function [236]. Overcoming these barriers requires a cross‐disciplinary integration of materials science, microbiology, and biomedical engineering. Materials scientists contribute to the rational design of polymer networks with optimized mechanical strength, permeability, and hydration to balance protection with metabolic exchange. Microbiologists identify robust microbial strains with enhanced tolerance to encapsulation stress and physiological fluctuations. Bioengineers introduce advanced fabrication techniques, such as microfluidics and extrusion‐based 3D printing, to precisely control microscale architecture and spatial localization of microbial cells within hydrogel scaffolds. Moreover, the integration of decellularized extracellular matrix components into hydrogel formulations provides biologically relevant cues that enhance microbial adhesion, proliferation, and bioactivity. These biomimetic modifications also improve hydrogel biocompatibility and may facilitate crosstalk between encapsulated microorganism and host tissues. Looking ahead, sustained progress in this field will depend on tight collaboration across disciplines to create next‐generation living hydrogels that combine structural integrity, biological resilience, and precise site‐specific functionality. Achieving this integration will be pivotal to unlocking the full therapeutic potential of living microbial systems in complex physiological environments.

### 7.3 Design Complexity of Multi‐Species Collaborative Systems

The integration of multi‐strain living microbial hydrogel systems represents a major frontier in living material design but also introduces significant complexity in both material architecture and biological coordination. Unlike single‐strain formulations, which rely on homogeneous metabolic behavior, multi‐species systems must support spatial segregation, functional differentiation, and interspecies cooperation within a shared and physiologically relevant matrix. Achieving this requires hydrogel architectures that provide distinct yet interactive microenvironments, enabling each microbial population to maintain viability, niche compatibility, and cooperative metabolic function over extended periods. To this end, spatially organized encapsulation strategies—including microfluidic‐assisted fabrication, layer‐by‐layer assembly, and coaxial or droplet‐based compartmentalization—have been explored to achieve controlled localization while preserving diffusional communication across microbial domains [237]. However, these approaches introduce further complexity in scaling and reproducibility.

Beyond fabrication, coordinating multiple microbial species within a single hydrogel scaffold introduces inherent biological challenges. Maintaining strain stability, metabolic compatibility, and cooperative mutualism becomes increasingly difficult in fluctuating environments characterized by nutrient gradients, pH shifts, and host immune pressures. In particular, preserving phenotypic stability and cooperative behavior during prolonged therapeutic deployment remains a critical bottleneck [238]. Moreover, the functional design of these systems must incorporate controlled release of microbial metabolites, quorum sensing molecules, or therapeutic agents, which demands hydrogel matrices with finely tunable diffusivity and responsiveness to both microbial and host‐derived cues [239].

Future progress will depend on integrating adaptive hydrogel architectures with real‐time regulatory feedback, employing synthetic biology to program microbial communication networks and resilience, and leveraging advanced fabrication techniques such as 3D bioprinting and modular microgel assembly to achieve scalable, reproducible, and hierarchically organized consortia. Such efforts will pave the way for programmable, multi‐species living materials capable of dynamic self‐organization, cooperative metabolism, and intelligent therapeutic responses.

### 7.4 Biocompatibility and Immunogenicity of Living Hydrogels

In living hydrogel systems for microbial delivery, biocompatibility extends beyond the chemical inertness of the material—it arises from the dynamic interactions among the hydrogel matrix, the encapsulated microorganisms, and the host tissues. Unlike traditional inert biomaterials, these systems host metabolically active microorganisms capable of secreting enzymes, metabolites, and surface‐associated molecules that directly influence host cells. Therefore, assessing biocompatibility requires a dual approach: evaluating the physicochemical safety of the hydrogel matrix itself, and elucidating how microbial activity modulates the local immune microenvironment.

At the material level, the chemical composition and structural properties of the hydrogel are key determinants of host immune responses. For instance, alginate has been widely recognized for its low immunogenicity, but its purity and crosslinking agents can modulate inflammatory responses [240]. Similarly, chitosan, though biodegradable and mucoadhesive, may trigger innate immune activation via Toll‐like receptor (TLR) pathways depending on its molecular weight and deacetylation degree [234]. These findings highlight the importance of standardized material characterization—including composition, crosslinking density, and degradation profile—when evaluating hydrogel immunogenicity.

At the microbial level, encapsulated living microorganism can interact with host epithelial or immune cells directly upon release or indirectly through soluble microbial‐associated molecule such as lipoteichoic acid or peptidoglycan. Although these interactions can be beneficial in modulating mucosal immunity and gut homeostasis, they also pose a risk of unwanted immune activation, especially when the release is uncontrolled or occurs at non‐target sites. Encapsulation may delay or attenuate this response, but does not eliminate it, especially when the hydrogel degrades or when low‐level diffusion of microbial metabolites persists [167, 241]. Importantly, both the microbial strain and its associated molecular signatures shape the immunological outcome, which is further influenced by the host immune profile. Individuals with compromised epithelial barriers or pre‐existing inflammatory conditions may respond more aggressively to both the hydrogel matrix and microbial components, leading to heightened immunogenicity. Thus, rigorous immunological evaluation should integrate material properties, strain‐specific characteristics, and host phenotypes, with particular attention to distinctions between inflamed and homeostatic tissue environments. In future, addressing the immunological complexities of living hydrogel systems demands the integration of advanced immunoassays, including flow cytometry, high‐throughput transcriptomics, and 3D co‐culture models to more accurately simulate mucosal immunity. Only through a detailed mechanistic understanding of these interactions can researchers rationally design hydrogel systems that are both immunologically silent and functionally effective.

In summary, the path toward clinically and environmentally safe living hydrogels demands a holistic evaluation of biocompatibility, and immunogenicity across biological, material, and ecological dimensions. Only through such integrative design and monitoring frameworks can these hybrid systems transition safely from laboratory innovation to real‐world biomedical applications.

### 7.5 Biosafety of Living Hydrogels

Biosafety remains a critical consideration in the clinical translation of living hydrogels. While controlled hydrogel degradation is often essential for therapeutic function—such as enabling microbial release or facilitating tissue remodeling—unregulated degradation poses significant risks. A key concern is the unintended dissemination of living microorganisms into surrounding tissues or systemic circulation upon matrix breakdown, particularly in open wounds or highly vascularized environments. Such leakage can trigger local infections, bacteremia, or even sepsis, especially in immunocompromised patients or those with compromised epithelial barriers.

In oncological settings, biosafety challenges are compounded by the use of cytotoxic therapies, including chemotherapy, radiotherapy, and immunotherapy. These treatments not only suppress host immune defenses but may also compromise the viability and therapeutic efficacy of encapsulated microbes. Moreover, cytotoxic stress may accelerate hydrogel degradation, leading to premature microbial release. Given that many microbial‐based strategies rely on active metabolism or in situ proliferation, preserving microbial function in these hostile environments is particularly demanding. To address these concerns, next‐generation living hydrogels must incorporate multi‐layered biosafety mechanisms. These include: (1) stimuli‐responsive degradation kinetics that enable spatiotemporal control over microbial release; (2) physical confinement architectures that limit microbial dissemination post‐degradation; and (3) genetically encoded kill‐switches that trigger microbial self‐elimination in response to loss of hydrogel integrity or specific environmental cues. Additionally, integrating real‐time biosensors to monitor hydrogel stability and microbial activity may provide early warning of system failure, allowing timely clinical intervention. Together, these design principles aim to balance therapeutic efficacy with robust safety control, which is essential for the successful clinical implementation of living hydrogels.

Ecological containment constitutes another essential but frequently overlooked dimension of biosafety. Following in vivo use, hydrogel residues and microbial remnants may be excreted into external environments, where they may encounter microbial ecosystems vastly different from their original host context. The resulting horizontal gene transfer events, biofilm formation, or niche colonization by genetically modified strains could pose unforeseen ecological risks, particularly under anthropogenic selection pressures such as antibiotic residues or heavy metals [84]. To mitigate this risk, future efforts should adopt an integrated biosafety framework that includes post‐release tracking systems, genetically encoded biosensors, optogenetic tags or inert imaging contrast agents to monitor the fate and spread of both materials and living microorganisms in real time. Furthermore, incorporating orthogonal safeguards, such as multi‐input kill‐switches or redundant suicide circuits, can minimize the likelihood of failure due to mutational inactivation.

### 7.6 Challenges of Living Hydrogel Clinical Transformation

Although hydrogels are increasingly valued for their biocompatibility, tunability, and protective encapsulation capacity, the clinical translation of living hydrogels still faces significant challenges. A major challenge arises from the lack of standardized materials and fabrication protocols which introduce batch‐to‐batch variability that compromises reproducibility and clinical consistency. Differences in polymer sources, crosslinking methods, and encapsulation conditions can result in hydrogels with divergent degradation profiles, release kinetics, and immunogenicity. Without consensus on standardized formulations and manufacturing processes, it is challenging to ensure comparability across clinical trials [242]. A further and often underappreciated bottleneck lies in the limited translational fidelity of preclinical models. Most current evaluations rely on in vitro assays or rodent models, which fail to capture the structural, immunological, and microbial complexity of the human gastrointestinal ecosystem. Differences in microbial diversity, immune surveillance mechanisms, gut architecture, and transit time create a translational mismatch, often resulting in overestimation of living microbial retention, colonization, or therapeutic impact. Furthermore, hydrogels that perform effectively in animal models may encounter unexpected degradation, altered living microbial behavior, or immune rejection when introduced into human hosts. Future efforts should adopt more sophisticated human‐relevant models, such as ex vivo intestinal tissues, microfluidic gut‐on‐chip platforms, and controlled human microbiota‐associated models.

In addition, regulatory evaluation is another complex and evolving challenge faced by the clinical transformation of living hydrogels. Unlike conventional probiotics that are often regulated as dietary supplements, hydrogels designed as vehicles for living microorganisms intended to prevent or treat disease are subject to stringent pharmaceutical standards for biological products. In the United States, such systems fall under the regulatory oversight of the FDA's Center for Biologics Evaluation and Research, which requires submission of an Investigational New Drug (IND) application and compliance with current good manufacturing practices (cGMP). Critical requirements include thorough characterization of both the hydrogel matrix and the encapsulated microbial strains, encompassing identity, viability, purity, and genetic stability, particularly when using recombinant strains [243]. Additionally, regulatory scrutiny increasingly emphasizes the need for robust data on the interaction between delivery matrices and microbial viability, release kinetics, and safety under physiological and pathological conditions. Regulatory agencies also require developers to evaluate the risk of horizontal gene transfer, persistence in non‐target sites, and potential impacts on host microbiota. These concerns are particularly pronounced when using engineered strains or encapsulation strategies that extend microbial viability or delay clearance [244]. Another layer of complexity arises from the diversity of administration routes (e.g., oral, topical, or injectable), which influence requirements for toxicity studies, biodistribution analysis, and biocontainment design. In Europe, the European Medicines Agency (EMA) and the European Pharmacopoeia have developed specific standards for live biotherapeutic products, mandating quality control measures such as quantification of viable units, verification of contaminant‐free preparations, and genetic stability of the strains over time [245]. To address these challenges, regulatory‐aware design should begin early. This includes designing genetic tracking tools, safety modules for microbial clearance, and standardized, scalable formulation protocols aligned with cGMP. Advancing such platforms will require close collaboration among microbiologists, materials scientists, and regulatory experts to ensure safety, efficacy, and translational viability.

## 8 Conclusion

The convergence of engineering, biology, and materials science has opened transformative opportunities for integrating living microorganisms into hydrogel systems, giving rise to living hydrogels as a new class of dynamic, multifunctional therapeutics. Central to their functionality is the reciprocal interaction between microbial metabolism and hydrogel architecture, through which each component dynamically modulates the other's structural and biological performance. In this review, we summarized recent advances in material design strategies, fabrication approaches, and synergistic mechanisms underlying living hydrogel systems. From a materials perspective, diverse hydrogel networks have been engineered with tunable mechanical properties, biocompatibility, and porosity to support microbial viability and activity over extended periods. Encapsulation strategies—such as seeding‐from, seeding‐to, and electrostatic self‐assembly—have further improved the structural stability and responsiveness of hydrogels under physiologically relevant conditions. Equally important, understanding the dynamic interactions between hydrogel matrices and microorganisms has led to the rational design of functional living materials that go beyond passive encapsulation. Hydrogels composed of prebiotic components can serve as selective nutrient substrates, promoting microbial proliferation, colonization, and metabolic activity, while certain hydrogel matrices exhibit intrinsic biological functions, including anti‐inflammatory, antioxidant, or immunomodulatory effects. The synergistic integration of these material and microbial functions has enabled living hydrogels to achieve superior outcomes in wound healing, intestinal disorders, cancer therapy, and other disease contexts.

Looking ahead, realizing the full therapeutic potential of living hydrogels will require continued interdisciplinary collaboration to unravel their mechanistic complexity, enhance their biosafety and functional stability, and establish scalable fabrication and regulatory standards. Through such concerted efforts, living hydrogels may evolve from experimental platforms into clinically viable, intelligent biohybrid therapeutics capable of dynamic adaptation, precision modulation, and long‐term integration within living systems.

## Author Contributions

**S.M**.: writing – original draft, data curation. **B.B**.: writing – original draft, data curation. **X.Y**.: writing – review & editing, supervision, funding acquisition. **Y.L**.: writing – review & editing, supervision, funding acquisition, conceptualization.

## Conflicts of Interest

The authors declare no conflicts of interest.

## Data Availability

No data was used for the research described in the article.

## References

[1] J. Wilde , E. Slack , and K. R. Foster , “Host Control of the Microbiome: Mechanisms, Evolution, and Disease,” Science 385 (2024): 6706, 10.1126/science.adi3338. 39024451

[2] J. L. Lan , Y. X. Zhang , C. Y. Jin , et al., “Gut Dysbiosis Drives Inflammatory Bowel Disease Through the CCL4L2‐VSIR Axis in Glycogen Storage Disease,” Advanced Science 11 (2024): 2309471. 38889269 10.1002/advs.202309471 PMC11321658

[3] Y. Shen , N. R. Fan , S. X. Ma , et al., “Gut Microbiota Dysbiosis: Pathogenesis, Diseases, Prevention,” Medcomm 6 (2025): 70168. 10.1002/mco2.70168 PMC12006732 40255918

[4] Y. H. Wu , Y. H. Jiang , Y. R. Gong , et al., “A "Janus" Structured Nanoclay Microgel System for Targeted Probiotic Therapy in Diarrhea‐predominant Irritable Bowel Syndrome,” J Control Release 383 (2025): 113769. 40280239 10.1016/j.jconrel.2025.113769

[5] Y. Tu and W. J. Rappel , “Adaptation in Living Systems,” Annual Review of Condensed Matter Physics 9 (2018): 183. 10.1146/annurev-conmatphys-033117-054046 PMC6060625 30057689

[6] C. L. Hayes , J. M. Natividad , J. Jury , R. Martin , P. Langella , and E. F. Verdu , “Efficacy of Bifidobacterium Breve NCC2950 against DSS‐induced Colitis Is Dependent on Bacterial Preparation and Timing of Administration,” Beneficial Microbes 5 (2014): 79–88, 10.3920/BM2013.0039. 24533977

[7] A. Sivan , L. Corrales , N. Hubert , et al., “Commensal Bifidobacterium Promotes Antitumor Immunity and Facilitates Anti–PD‐L1 Efficacy,” Science 350 (2015): 1084–1089, 10.1126/science.aac4255. 26541606 PMC4873287

[8] Q. Li , R. de Oliveira Formiga , V. Puchois , et al., “Microbial Metabolite Indole‐3‐propionic Acid Drives Mitochondrial Respiration in CD4^+^ T Cells to Confer Protection against Intestinal Inflammation,” Nature Metabolism 7 (2025): 2510–2530, 10.1038/s42255-025-01396-6. PMC12727523 41120706

[9] T. S. Ghosh , F. Shanahan , and P. W. O'Toole , “The Gut Microbiome as a Modulator of Healthy Ageing,” Nature Reviews Gastroenterology & Hepatology 19 (2022): 565–584, 10.1038/s41575-022-00605-x. 35468952 PMC9035980

[10] X. Chen , Y. Chen , C. Stanton , et al., “Protective Effects of Bifidobacterium Breve on Imiquimod‐induced Psoriasis in Mice through Secondary Bile Acid Production and FXR‐TLR4/NF‐κB Pathway,” Food Science and Human Wellness 13 (2024): 3447.

[11] J. Deng , Y. Hu , P. Zhu , et al., “Probiotic Delivery for Editing of the Gut Microbiota to Mitigate Colitis and Maintain Hepatic Homeostasis via Gut–Liver Axis,” ACS Nano 19 (2025): 10500. 40047584 10.1021/acsnano.5c00325

[12] X. Qian , Q. Li , H. Zhu , et al., “Bifidobacteria with Indole‐3‐lactic Acid‐producing Capacity Exhibit Psychobiotic Potential via Reducing Neuroinflammation,” Cell Reports Medicine 5 (2024): 101798, 10.1016/j.xcrm.2024.101798. 39471819 PMC11604549

[13] H. Liu , Z. Chen , Q. Lin , et al., “A Multicellular Self‐organized Probiotic Platform for Oral Delivery Enhances Intestinal Colonization,” Nature Communications 16 (2025): 7060, 10.1038/s41467-025-62349-x. PMC12317005 40750778

[14] B. D. Knapp , L. Willis , C. Gonzalez , et al., “Metabolic Rearrangement Enables Adaptation of Microbial Growth Rate to Temperature Shifts,” Nature microbiology 10 (2025): 185–201. 10.1038/s41564-024-01841-4 39672961

[15] H. Huang , X. Y. Liu , Y. T. Lang , J. R. Cui , D. N. Zhong , and M. Zhou , “Bio‐Inspired Nanovehicles for Cancer Vaccination,” Journal of Nanbiotechnology 22 (2024): 203.

[16] M. K. Heavey , A. Hazelton , Y. Y. Wang , et al., “Targeted Delivery of the Probiotic Saccharomyces Boulardii to the Extracellular Matrix Enhances Gut Residence Time and Recovery in Murine Colitis,” Nature Communications 15 (2024): 3784. 10.1038/s41467-024-48128-0 PMC11074276 38710716

[17] X. Liu , J. Liu , S. Lin , and X. Zhao , “Hydrogel Machines,” Materials Today 36 (2020): 102–124, 10.1016/j.mattod.2019.12.026.

[18] H. Zhao , M. Liu , Y. Zhang , J. Yin , and R. Pei , “Nanocomposite Hydrogels for Tissue Engineering Applications,” Nanoscale 12 (2020): 14976–14995, 10.1039/D0NR03785K. 32644089

[19] B. Li , D. Zhou , and Y. Han , “Assembly and Phase Transitions of Colloidal Crystals,” Nature Reviews Materials 1 (2016): 15011.

[20] Z. Qiao , J. Parks , P. Choi , and H. F. Ji , “Applications of Highly Stretchable and Tough Hydrogels,” Polymers 11 (2019): 1773. 31661812 10.3390/polym11111773 PMC6918353

[21] Q. R. Feng , Y. Luo , M. Liang , et al., “Rhizobacteria Protective Hydrogel to Promote Plant Growth and Adaption to Acidic Soil,” Nature Communications 16 (2025): 1684. 10.1038/s41467-025-56988-3 PMC11830790 39956869

[22] A. C. Daly , L. Riley , T. Segura , and J. A. Burdick , “Hydrogel Microparticles for Biomedical Applications,” Nature Reviews Materials 5 (2020): 20–43, 10.1038/s41578-019-0148-6. PMC8191408 34123409

[23] H. Fang , Y. Wang , L. Li , et al., “Microenvironment‐responsive Living Hydrogel Containing Engineered Probiotic for Treatment of Massive Bone Defects,” Bioactive Materials 50 (2025): 556. 40385972 10.1016/j.bioactmat.2025.04.020 PMC12083996

[24] H. Xu , Y. Li , J. Song , et al., “Highly Active Probiotic Hydrogels Matrixed on Bacterial EPS Accelerate Wound Healing via Maintaining Stable Skin Microbiota and Reducing Inflammation,” Bioactive Materials 35 (2024): 31. 38304916 10.1016/j.bioactmat.2024.01.011 PMC10831122

[25] Y. Zhu , Q. Wang , Y. Chen , et al., “Living Probiotics‐Loaded Hydrogel Microspheres with Gastric Acid Resistance and ROS Triggered Release for Potential Therapy of Inflammatory Bowel Disease,” ACS Applied Polymer Materials 5 (2023): 957.

[26] W. Li , J. X. Fan , J. Y. Qiao , Q. W. Chen , Y. X. Sun , and X. Z. Zhang , “Colon‐targeted Bacterial Hydrogel for Tumor Vascular Normalization and Improved Chemotherapy,” Journal of Controlled Release 356 (2023): 59–71, 10.1016/j.jconrel.2023.02.028. 36842488

[27] K. Zhang , Y. Liang , Q. Chen , et al., “Orchestrating Gut Disorders by Oral Delivery of a Living–Synthetic Hybrid Hydrogel,” ACS Nano 19 (2025): 27825–27844, 10.1021/acsnano.5c08982. 40712055

[28] M. Karampoor , A. Fouladpour , S. Yavari , et al., “Probiotics as a Promising Treatment Approach to Burn Wound Healing,” Burns 48 (2022): 2003. 35995641 10.1016/j.burns.2022.07.003

[29] Z. Ming , L. Han , M. Bao , et al., “Living Bacterial Hydrogels for Accelerated Infected Wound Healing,” Advanced Science 8 (2021): 2102545, 10.1002/advs.202102545. 34719880 PMC8693052

[30] Y. Lai , H. Shen , S. Wang , et al., “Hydrogel‐Transformable Probiotic Powder for Targeted Eradication of Helicobacter pylori with Enhanced Gastric Mucosal Repair and Microbiota Preservation,” Advancement of Science 12 (2025): 2500478. 10.1002/advs.202500478 PMC12199445 40091425

[31] L. Huang , J. Wang , L. Kong , et al., “ROS‐responsive Hyaluronic Acid Hydrogel for Targeted Delivery of Probiotics to Relieve Colitis,” International Journal of Biological Macromolecules 222 (2022): 1476–1486, 10.1016/j.ijbiomac.2022.09.247. 36195227

[32] Y. Wang , Y. Qu , X. Liu , et al., “Dual‐Functional Probiotic Hydrogel with Puerarin Integration for Microbiota‐Neuroimmune Regulation in Antibiotic‐Free Periodontitis Therapy,” Bioact Mater 53 (2025): 72. 40688024 10.1016/j.bioactmat.2025.07.004 PMC12274845

[33] Y. Chen , M. Shui , H. Li , et al., “Inflammation‐Targeted Delivery of Probiotics for Alleviation of Colitis and Associated Cognitive Disorders through Improved Vitality and Colonization,” Biomaterials 318 (2025): 123163. 39923539 10.1016/j.biomaterials.2025.123163

[34] A. X. Chen , J. M. Zhu , R. Liu , et al., “Injectable thermo‐sensitive hydrogel enhances anti‐tumor potency of engineered Lactococcus lactis by activating dendritic cells and effective memory T cells,” Bioactive Materials 37 (2024): 331. 38694762 10.1016/j.bioactmat.2024.03.023 PMC11061616

[35] X. Liu , M. E. Inda , Y. Lai , T. K. Lu , and X. Zhao , “Engineered Living Hydrogels,” Advanced Materials 34 (2022): 2201326. 10.1002/adma.202201326 PMC9250645 35243704

[36] R. Zhang , X. Liu , Y. Gou , et al., “Living‐loaded Hydrogel: Strategies for Loading Living, Interactions between Loaded Living and Hydrogel, and Applications,” European Polymer Journal 213 (2024): 113130, 10.1016/j.eurpolymj.2024.113130.

[37] X. Dong , W. Wu , P. Pan , and X. Z. Zhang , “Engineered Living Materials for Advanced Diseases Therapy,” Adv, Mater 37 (2025): 2304963. 10.1002/adma.202304963 37436776

[38] Y. F. Cui , M. W. Tibbitt , T. K. Lu , and T. C. Tang , “Design principles for adaptive and evolving engineered living materials,” Curr Op Biotechnol 97 (2026): 103397. 10.1016/j.copbio.2025.103397 41344284

[39] H. Wilssens , L. De Wannemaeker , and M. De Mey , “Strength in Diversity: Unlocking the Full Potential of Engineered Living Materials with Multistrain Collaboration,” Fems Microbiology Review 49 (2025): fuaf055. 10.1093/femsre/fuaf055 PMC12671054 41206547

[40] Q. W. Wang , Z. H. Hu , Z. X. Li , T. A. Liu , and G. K. Bian , “Exploring the Application and Prospects of Synthetic Biology in Engineered Living Materials,” Advanced Materials 37 (2025): 2305828. 10.1002/adma.202305828 37677048

[41] Y. Zhang , Y. Zhang , M. Jian , et al., “Sustained‐release, Antibacterial, Adhesive Gelatin Composite Hydrogel with AgNPs Double‐capped with Curdlan Derivatives,” International Journal of Biological Macromolecules 277 (2024): 134222. 39074697 10.1016/j.ijbiomac.2024.134222

[42] S. F. Mao , Y. J. Zeng , Y. M. Ren , X. Q. Ye , and J. H. Tian , “EGCG induced the formation of protein nanofibrils hydrogels with enhanced anti‐bacterial activity,” Food Hydrocolloids 157 (2024): 110408.

[43] Y. J. Zeng , S. F. Mao , B. Y. Huang , X. Q. Ye , and J. H. Tian , “Formation of tannic acid‐binding ovalbumin amyloid fibril hydrogels: Enhanced antibacterial and antioxidant properties,” Food Hydrocolloids 156 (2024): 110333.

[44] R. G. Klemperer , M. R. Shannon , J. L. R. Anderson , and A. W. Perriman , “Bienzymatic Generation of Interpenetrating Polymer Networked Engineered Living Materials with Shape Changing Properties,” Advanced Materials Technologies 8 (2023): 2300626, 10.1002/admt.202300626.

[45] Y. Shao , H. Jia , T. Cao , and D. Liu , “Supramolecular Hydrogels Based on DNA Self‐Assembly,” Accounts of Chemical Research 50 (2017): 659–668, 10.1021/acs.accounts.6b00524. 28299927

[46] G. W. Liu , M. J. Pickett , J. L. P. Kuosmanen , et al., “Drinkable in Situ‐forming Tough Hydrogels for Gastrointestinal Therapeutics,” Nature Materials 23 (2024): 1292–1299, 10.1038/s41563-024-01811-5. 38413810 PMC11364503

[47] J. Cao , X. Zhang , J. Guo , et al., “An Engineering‐reinforced Extracellular Vesicle–integrated Hydrogel with an ROS‐responsive Release Pattern Mitigates Spinal Cord Injury,” Science Advances 11 (2025): 3398. 10.1126/sciadv.ads3398 PMC11963969 40173229

[48] Y. Lv , Z. Wang , Y. Wei , et al., “Thermoresponsive Dual‐Network Chitosan‐based Hydrogels with Demineralized Bone Matrix for Controlled Release of rhBMP9 in the Treatment of Femoral Head Osteonecrosis,” Carbohydrate Polymers 352 (2025): 123197. 39843099 10.1016/j.carbpol.2024.123197

[49] L. Li , M. E. Griebel , M. Uroz , et al., “A Protein‐Adsorbent Hydrogel with Tunable Stiffness for Tissue Culture Demonstrates Matrix‐Dependent Stiffness Responses,” Advanced Functional Materials 34 (2024): 2309567, 10.1002/adfm.202309567. 38693998 PMC11060701

[50] Y. Wang , M. Yang , and Z. Zhao , “Facile Fabrication of Self‐healing, Injectable and Antimicrobial Cationic Guar Gum Hydrogel Dressings Driven by Hydrogen Bonds,” Carbohydrate Polymers 310 (2023): 120723. 36925248 10.1016/j.carbpol.2023.120723

[51] B. Wu , Y. Jian , X. Le , et al., “Supramolecular Fabrication of Complex 3D Hollow Polymeric Hydrogels with Shape and Function Diversity,” ACS Applied Materials & Interfaces 11 (2019): 48564–48573, 10.1021/acsami.9b17440. 31742383

[52] X. Zhang , D. Li , X. Yang , et al., “Hydro‐Locking in Hydrogel for Extreme Temperature Tolerance,” Science 387 (2025): 967–973, 10.1126/science.adq2711. 40014727

[53] L. Wu , K. Schroen , and M. Corstens , “Structural Stability and Release Properties of Emulsion‐alginate Beads under Gastrointestinal Conditions,” Food Hydrocolloids 150 (2024): 109702.

[54] X. Chen , Y. Feng , D. Zhang , et al., “Orally Administered Hydrogel Containing Polyphenol@Halloysite Clay for Probiotic Delivery and Treatment of Inflammatory Bowel Disease,” Nano Today 62 (2025): 102669, 10.1016/j.nantod.2025.102669.

[55] H. Xin , Z. Cai , J. Hao , et al., “Macro/Microgel‐Encapsulated, Biofilm‐Armored Living Probiotic Platform for Regenerating Bacteria‐Infected Diabetic Wounds,” Advanced Healthcare Materials 14 (2025): 2403476. 10.1002/adhm.202403476 39831829

[56] D. Sharma , A. J. Middya , N. Singh , S. Singhal , and A. Dan , “Microscale Liquid Crystal Droplet‐Embedded Hydrogel Film for Ultrasensitive Optical Detection of Bacterial Endotoxins,” Small 21 (2025): 2503766. 10.1002/smll.202503766 40470613

[57] L. Zhao , L. Niu , H. Liang , H. Tan , C. Liu , and F. Zhu , “pH and Glucose Dual‐Responsive Injectable Hydrogels with Insulin and Fibroblasts as Bioactive Dressings for Diabetic Wound Healing,” ACS Applied Materials & Interfaces 9 (2017): 37563–37574, 10.1021/acsami.7b09395. 28994281

[58] Y. Luo , Z. Ma , C. De Souza , et al., “Microfluidic Fabrication of Encapsulated Probiotic Microspheres Using Cysteine‐Modified Chitosan with Dual Functions of Bacterial Adhesion and Intestinal Mucosal Adhesion,” Food Hydrocolloids 149 (2024): 109602, 10.1016/j.foodhyd.2023.109602.

[59] C. Zhou , Y. Zou , R. Xu , et al., “Metal‐Phenolic Self‐Assembly Shielded Probiotics in Hydrogel Reinforced Wound Healing with Antibiotic Treatment,” Materials Horizons 10 (2023): 3114–3123, 10.1039/D3MH00033H. 37218586

[60] R. Yang , J. Huang , W. Zhang , et al., “Mechanoadaptive Injectable Hydrogel Based on Poly (γ‐glutamic acid) and Hyaluronic Acid Regulates Fibroblast Migration for Wound Healing,” Carbohydrate Polymers 273 (2021): 118607. 34561006 10.1016/j.carbpol.2021.118607

[61] S. Li , Q. Dong , X. Peng , et al., “Self‐Healing Hyaluronic Acid Nanocomposite Hydrogels with Platelet‐Rich Plasma Impregnated for Skin Regeneration,” ACS Nano 16 (2022): 11346–11359, 10.1021/acsnano.2c05069. 35848721

[62] S. A. Sydlik , A. L. Watson , K. E. Eckhart , and M. E. Wolf , “Hyaluronic Acid‐based Antibacterial Hydrogels for Use as Wound Dressings,” ACS Applied Bio Materials 5 (2022): 5608. 10.1021/acsabm.2c00647 36383154

[63] N. Yang , X. Jike , M. Zhang , T. Jiang , and H. Lei , “Synthesis, Characterization of Thiolated Hyaluronic Acid and Evaluation of Its Encapsulation Effects on Limosilactobacillus Reuteri HR7,” International Journal of Biological Macromolecules 310 (2025): 143486. 40280531 10.1016/j.ijbiomac.2025.143486

[64] Y. Li , Z. Qin , P. He , et al., “Fully Degradable Protein Gels with Superior Mechanical Properties and Durability: Regulation of Hydrogen Bond Donors,” Advanced Materials 37 (2025): 2506577, 10.1002/adma.202506577. 40557495

[65] H. A. Tran , A. Maraldo , T. T.‐P. Ho , et al., “Probing the Interplay of Protein Self‐Assembly and Covalent Bond Formation in Photo‐Crosslinked Silk Fibroin Hydrogels,” Small 21 (2025): 2407923. 39548941 10.1002/smll.202407923 PMC12019910

[66] Z. Zhang , Y. Xia , X. Li , et al., “Arginine‐Solubilized Lipoic Acid‐Induced β‐Sheets of Silk Fibroin‐Strengthened Hydrogel for Postoperative Rehabilitation of Breast Cancer,” Bioactive Materials 40 (2024): 667–682. 39257958 10.1016/j.bioactmat.2024.08.014 PMC11386050

[67] Q. Wu , M. Chauhan , B. Khamaisi , E. Nassar‐Marjiya , and S. Farah , “Biomimetic 3D‐Printed Adaptive Hydrogel Bioadhesives Featuring Superior Infection Resistance for Challenging Tissue Adhesion, Hemostasis, and Healthcare,” Advanced Materials 37 (2025): 2502850, 10.1002/adma.202502850. 40693294 PMC12592912

[68] F. Chen , P. Le , G. M. Fernandes‐Cunha , S. C. Heilshorn , and D. Myung , “Bio‐orthogonally Crosslinked Hyaluronate‐Collagen Hydrogel for Suture‐Free Corneal Defect Repair,” Biomaterials 255 (2020): 120176. 32559566 10.1016/j.biomaterials.2020.120176 PMC7396293

[69] E. A. Aisenbrey and S. J. Bryant , “Mechanical Loading Inhibits Hypertrophy in Chondrogenically Differentiating hMSCs within a Biomimetic Hydrogel,” Journal of Materials Chemistry B 4 (2016): 3562–3574, 10.1039/C6TB00006A. 27499854 PMC4972607

[70] A. Gonzalez , L. Sabio , C. Hurtado , et al., “Entrapping Living Probiotics into Collagen Scaffolds: a New Class of Biomaterials for Antibiotic‐Free Therapy of Bacterial Vaginosis,” Advanced Materials Technologies 5 (2020): 2000137.

[71] Y. J. Jo , K. Y. Kwon , Z. U. Khan , X. Crispin , and T. I. Kim , “Gelatin Hydrogel‐Based Organic Electrochemical Transistors and Their Integrated Logic Circuits,” ACS Applied Materials & Interfaces 10 (2018): 39083–39090, 10.1021/acsami.8b11362. 30360103

[72] N. C. Cheng , W. J. Lin , T. Y. Ling , and T. H. Young , “Sustained Release of Adipose‐derived Stem Cells by Thermosensitive Chitosan/Gelatin Hydrogel for Therapeutic Angiogenesis,” Acta Biomaterialia 51 (2017): 258–267, 10.1016/j.actbio.2017.01.060. 28131942

[73] S. Khodami , K. Kaniewska , J. Romanski , M. Karbarz , and Z. Stojek , “Amino Acid‐Based Hydrogel with Interpenetrating Gelatin and Cross‐Linked by Metal Ions, Providing High Stretchability and Motion Sensitivity,” ACS Omega 10 (2025): 12062–12075, 10.1021/acsomega.4c10083. 40191295 PMC11966301

[74] S. Tao , S. Zhang , K. Wei , K. Maniura‐Weber , Z. Li , and Q. Ren , “An Injectable Living Hydrogel with Embedded Probiotics as a Novel Strategy for Combating Multifaceted Pathogen Wound Infections,” Advanced Healthcare Materials 13 (2024): 2400921. 38923269 10.1002/adhm.202400921 PMC12344615

[75] A. F. Corona‐Escalera , E. Tinajero‐Diaz , R. A. Garcia‐Reyes , et al., “Enzymatic Crosslinked Hydrogels of Gelatin and Poly (vinyl alcohol) Loaded with Probiotic Bacteria as Oral Delivery System,” Pharmaceutics 14 (2022): 2759. 36559253 10.3390/pharmaceutics14122759 PMC9784308

[76] J. L. Patarroyo , E. Fonseca , J. Cifuentes , F. Salcedo , J. C. Cruz , and L. H. Reyes , “Gelatin‐Graphene Oxide Nanocomposite Hydrogels for Kluyveromyces Lactis Encapsulation: Potential Applications in Probiotics and Bioreactor Packings,” Biomolecules 11 (2021): 922. 34206397 10.3390/biom11070922 PMC8302002

[77] S. Cui , Y. Li , Z. Xu , and X. Yu , “Bioinspired Conductivity‐Enhanced, Self‐Healing, and Renewable Silk Fibroin Hydrogel for Wearable Sensors with High Sensitivity,” ACS Applied Materials & Interfaces 17 (2025): 8657–8669, 10.1021/acsami.4c21099. 39873141

[78] J. Wang , N. Zhang , Y. Tan , et al., “Sweat‐Resistant Silk Fibroin‐Based Double Network Hydrogel Adhesives,” ACS Applied Materials & Interfaces 14 (2022): 21945–21953, 10.1021/acsami.2c02534. 35507426

[79] Q. Chen , Y. Sima , Z. Liu , et al., “Preparation and Properties of Semi‐Interpenetrating Silk Fibroin Protein Hydrogels with Integrated Strength and Toughness,” ACS Applied Polymer Materials 4 (2022): 735–745, 10.1021/acsapm.1c01670.

[80] J. O. Buitrago , K. D. Patel , A. El‐Fiqi , et al., “Silk Fibroin/Collagen Protein Hybrid Cell‐encapsulating Hydrogels with Tunable Gelation and Improved Physical and Biological Properties,” Acta Biomaterialia 69 (2018): 218–233, 10.1016/j.actbio.2017.12.026. 29410166

[81] D. Wang , X. Y. Li , and A. Li , “Natural bioink of interpenetrating network hydrogels mimicking extracellular polymeric substances for microbial immobilization in water pollution control,” Environmental Research 262 (2024): 119856. 39197485 10.1016/j.envres.2024.119856

[82] E. Axpe , A. Duraj‐Thatte , Y. Chang , et al., “Fabrication of Amyloid Curli Fibers–Alginate Nanocomposite Hydrogels with Enhanced Stiffness,” ACS Biomaterials Science & Engineering 4 (2018): 2100–2105, 10.1021/acsbiomaterials.8b00364. 33435033

[83] Z. Abdali , M. Aminzare , X. Zhu , et al., “Curli‐Mediated Self‐Assembly of a Fibrous Protein Scaffold for Hydroxyapatite Mineralization,” ACS Synthetic Biology 9 (2020): 3334–3343, 10.1021/acssynbio.0c00415. 33237760

[84] A. M. Duraj‐Thatte , N. M. D. Courchesne , P. Praveschotinunt , et al., “Genetically Programmable Self‐Regenerating Bacterial Hydrogels,” Advanced Materials 31 (2019): 1901826. 10.1002/adma.201901826 PMC6773506 31402514

[85] F. Li , L. N. Ye , L. Y. Zhang , et al., “Design of a genetically programmed barnacle‐curli inspired living‐cell bioadhesive,” Mater Today Bio 14 (2022): 100256. 10.1016/j.mtbio.2022.100256 PMC9034392 35469253

[86] X. Lu , T. H. Perera , A. B. Aria , and L. A. S. Callahan , “Polyethylene Glycol in Spinal Cord Injury Repair: A Critical Review,” Journal of Experimental Pharmacology 10 (2018): 37–49, 10.2147/JEP.S148944. 30100766 PMC6067622

[87] J. M. Harris and R. B. Chess , “Effect of Pegylation on Pharmaceuticals,” Nature Reviews Drug Discovery 2 (2003): 214–221, 10.1038/nrd1033. 12612647

[88] N. A. Peppas , K. B. Keys , M. Torres‐Lugo , and A. M. Lowman , “Poly(ethylene glycol)‐containing Hydrogels in Drug Delivery,” Journal of Controlled Release 62 (1999): 81–87, 10.1016/S0168-3659(99)00027-9. 10518639

[89] M. S. Hahn , M. K. McHale , E. Wang , R. H. Schmedlen , and J. L. West , “Physiologic Pulsatile Flow Bioreactor Conditioning of Poly(ethylene glycol)‐based Tissue Engineered Vascular Grafts,” Annals of Biomedical Engineering 35 (2007): 190–200, 10.1007/s10439-006-9099-3. 17180465

[90] A. Degirmenci , R. Sanyal , and A. Sanyal , “Metal‐Free Click‐Chemistry: a Powerful Tool for Fabricating Hydrogels for Biomedical Applications,” Bioconjugate Chemistry 35 (2024): 433–452, 10.1021/acs.bioconjchem.4c00003. 38516745 PMC11036366

[91] Y. Liang , J. Shan , C. Tan , et al., “A Highly Adhesive and Melatonin‐loaded PEG Hydrogel Prevents Tumor Recurrence and Promotes Wound Healing for Tumor‐resection Wound Management of Liposarcoma,” Materials Today Bio 32 (2025): 101842. 10.1016/j.mtbio.2025.101842 PMC12136914 40469699

[92] O. Baghdasaryan , J. Lee‐Kin , and C. Tan , “Architectural Engineering of Cyborg Bacteria with Intracellular Hydrogel,” Materials Today Bio 28 (2024): 101226. 10.1016/j.mtbio.2024.101226 PMC11426140 39328785

[93] Y. Xiong , Q. Zhang , J. Li , et al., “Light‐sensitive PEG hydrogel with antibacterial performance for pacemaker pocket infection prevention,” Materials Today Bio 25 (2024): 100987. 10.1016/j.mtbio.2024.100987 PMC10938169 38486799

[94] M. K. Livingston , M. M. Morgan , W. T. Daly , et al., “Evaluation of PEG‐Based Hydrogel Influence on Estrogen‐Receptor‐Driven Responses in MCF7 Breast Cancer Cells,” ACS Biomaterials Science & Engineering 5 (2019): 6089–6098, 10.1021/acsbiomaterials.9b00480. 31942444 PMC6961958

[95] I. Kwiecien , D. Niewolik , A. I. Ekere , A. Gupta , and I. Radecka , “Synthesis of Hydrogels Made of Poly‐γ‐glutamic Acid (γ‐PGA) for Potential Applications as Probiotic‐delivery Vehicles,” Applied Sciences 10 (2020): 2787.

[96] T. Wang , W. Zhao , Y. Wu , et al., “Nanogel‐Reinforced Polyacrylamide Hydrogel for Potential Vascular Adhesion,” ACS Applied Polymer Materials 5 (2023): 1169–1179, 10.1021/acsapm.2c01649.

[97] C. Wang , X. Guan , Y. Yuan , Y. Wu , and S. Tan , “Polyacrylamide Crosslinked by Bis‐vinylimidazolium Bromide for High Elastic and Stable Hydrogels,” RSC Advances 9 (2019): 27640. 35529219 10.1039/c9ra05201a PMC9070750

[98] X. Liu , T. C. Tang , E. Tham , et al., “Stretchable Living Materials and Devices with Hydrogel‐Elastomer Hybrids Hosting Programmed Cells,” PANS 14 (2017): 2200. 10.1073/pnas.1618307114 PMC5338509 28202725

[99] Y. Zhou and L. Jin , “Hydrolysis‐induced Large Swelling of Polyacrylamide Hydrogels,” Soft Matter 16 (2020): 5740–5749, 10.1039/D0SM00663G. 32525191

[100] F. Milos and A. del Campo , “Polyacrylamide Hydrogels as Versatile Biomimetic Platforms to Study Cell‐Materials Interactions,” Advanced Materials Interfaces 11 (2024): 2400404.

[101] S. Y. Lee , S. I. Jeon , S. B. Sim , Y. Byun , and C. H. Ahn , “A Supramolecular Host‐Guest Interaction‐Mediated Injectable Hydrogel System with Enhanced Stability and Sustained Protein Release,” Acta Biomaterialia 131 (2021): 286–301, 10.1016/j.actbio.2021.07.004. 34246803

[102] Y. Almoshari , R. Ren , H. Zhang , et al., “GSK3 inhibitor‐loaded Osteotropic Pluronic Hydrogel Effectively Mitigates Periodontal Tissue Damage Associated with Experimental Periodontitis,” Biomaterials 261 (2020): 120293. 32877763 10.1016/j.biomaterials.2020.120293 PMC7541605

[103] S. Bhusari , J. Kim , K. Polizzi , S. Sankaran , and A. del Campo , “Encapsulation of Bacteria in Bilayer Pluronic Thin Film Hydrogels: a Safe Format for Engineered Living Materials,” Biomaterial Advances 145 (2023): 213240. 10.1016/j.bioadv.2022.213240 36577192

[104] K. Lin , S. Wang , L. Fan , D. Pan , C. J. Xian , and J. Shen , “Adipose‐derived stem cells seeded in Pluronic F‐127 hydrogel promotes diabetic wound healing,” J Sur Res 217 (2017): 63. 10.1016/j.jss.2017.04.032 28595815

[105] J. Yang , Z. Chen , D. Pan , H. Li , and J. Shen , “Umbilical Cord‐Derived Mesenchymal Stem Cell‐Derived Exosomes Combined Pluronic F127 Hydrogel Promote Chronic Diabetic Wound Healing and Complete Skin Regeneration,” International Journal of Nanomedicine 15 (2020): 5911–5926, 10.2147/IJN.S249129. 32848396 PMC7429232

[106] C. Huang , J. Teng , W. Liu , J. Wang , and A. Liu , “Modulation of Macrophages by a Phillyrin‐Loaded Thermosensitive Hydrogel Promotes Skin Wound Healing in Mice,” Cytokine 177 (2024): 156556. 38417214 10.1016/j.cyto.2024.156556

[107] F. Mo , L. Hang , M. Xu , et al., “Rational Design of Dynamically Super‐Tough and Super‐Stretchable Hydrogels for Deformable Energy Storage Devices,” Small 20 (2024): 2305557. 10.1002/smll.202305557 38193273

[108] K. G. Noh and S. Y. Park , “Biosensor Array of Interpenetrating Polymer Network with Photonic Film Templated from Reactive Cholesteric Liquid Crystal and Enzyme‐Immobilized Hydrogel Polymer,” Advanced Functional Materials 28 (2018): 1707562.

[109] W. Yan , X. Jia , Q. Zhang , H. Chen , Q. Zhu , and L. Yin , “Interpenetrating Polymer Network Hydrogels of Soy Protein Isolate and Sugar Beet Pectin as a Potential Carrier for Probiotics,” Food Hydrocolloids 113 (2021): 106453.

[110] Y. Wang , J. Ma , W. Cai , et al., “Fast Encapsulation of Microbes into Dissolvable Hydrogel Beads Enables High‐Throughput Microbial Single‐Cell RNA Sequencing of Clinical Microbiome Samples,” Advanced Materials 37 (2025): 2500481, 10.1002/adma.202500481. 40200683

[111] Y. Jeong and J. Irudayaraj , “Hierarchical Encapsulation of Bacteria in Functional Hydrogel Beads for Inter‐ and Intra‐ species Communication,” Acta Biomaterialia 158 (2023): 203–215, 10.1016/j.actbio.2023.01.003. 36632875 PMC10209895

[112] R. Xie , Y. C. Chen , Y. Zhao , et al., “Injectable Hydrogel Capable of In Situ Covalent Crosslinking for Permanent Embolization,” ACS Applied Materials & Interfaces 13 (2021): 56988–56999, 10.1021/acsami.1c18250. 34806359

[113] F. Ni , X. Luo , Z. Zhao , et al., “Enhancing Viability of Lactobacillus Plantarum Encapsulated by Alginate‐gelatin Hydrogel Beads during Gastrointestinal Digestion, Storage and in the Mimic Beverage Systems,” International Journal of Biological Macromolecules 224 (2023): 94–104, 10.1016/j.ijbiomac.2022.10.106. 36244533

[114] T. Wang , X. Ren , Y. Bai , L. Liu , and G. Wu , “Adhesive and Tough Hydrogels Promoted by Quaternary Chitosan for Strain Sensor,” Carbohydrate Polymers 254 (2021): 117298. 33357866 10.1016/j.carbpol.2020.117298

[115] T. Yao , J. Wang , Y. Yao , L. Fu , and L. Li , “Enhanced Survival of Probiotics by Encapsulating in Hydrogel Beads Based on Sodium Alginate/Pectin/Tannic Acid,” ACS Food Science & Technology 5 (2025): 1979–1989, 10.1021/acsfoodscitech.5c00223.

[116] Q. Wu , Z. Lu , L. Wang , et al., “Konjac glucomannan/Xanthan Gum Hydrogels Loaded with Metal‐phenolic Networks Encapsulated Probiotic to Promote Infected Wound Healing,” Carbohydrate Polymers 353 (2025): 123243. 39914948 10.1016/j.carbpol.2025.123243

[117] J. H. M. Wong , J. J. Chang , C. Owh , et al., “Dynamic Covalent Hydrogels for Wound Healing,” Annual Review of Chemical and Biomolecular Engineering 16 (2025): 93–117, 10.1146/annurev-chembioeng-082323-093537. 40067962

[118] M. Podgorski , B. D. Fairbanks , B. E. Kirkpatrick , et al., “Toward Stimuli‐Responsive Dynamic Thermosets through Continuous Development and Improvements in Covalent Adaptable Networks (CANs),” Advanced Materials 32 (2020): 1906876. 10.1002/adma.201906876 32057157

[119] L. Yang , Z. Han , C. Chen , et al., “Novel probiotic‐bound oxidized *Bletilla striata* polysaccharide‐chitosan composite hydrogel,” Mat Sci Eng C‐Mater 117 (2020): 111265. 10.1016/j.msec.2020.111265 32919631

[120] Y. Chen , D. Diaz‐Dussan , D. Wu , et al., “Bioinspired Self‐Healing Hydrogel Based on Benzoxaborole‐Catechol Dynamic Covalent Chemistry for 3D Cell Encapsulation,” ACS Macro Letters 7 (2018): 904. 35650963 10.1021/acsmacrolett.8b00434

[121] A. Prindle , P. Samayoa , I. Razinkov , T. Danino , L. S. Tsimring , and J. Hasty , “A Sensing Array of Radically Coupled Genetic ‘Biopixels’,” Nature 481 (2012): 39–44, 10.1038/nature10722. PMC3259005 22178928

[122] Z. Zeng , T. Wang , Y. Yang , et al., “Novel Silk Fibroin/Chitosan Microgel for Enhanced Probiotic Delivery: Improved Stability, Viability, and Targeted Release in Gastrointestinal Conditions,” Carbohydrate Polymers 368 (2025): 124191, 10.1016/j.carbpol.2025.124191. 40947266

[123] C. Zhang , X. Gao , X. Ren , et al., “Bacteria‐Induced Colloidal Encapsulation for Probiotic Oral Delivery,” ACS Nano 17 (2023): 6886–6898, 10.1021/acsnano.3c00600. 36947056

[124] X. Zhang , Y. Liang , S. Huang , and B. Guo , “Chitosan‐based Self‐healing Hydrogel Dressing for Wound Healing,” Advances in Colloid and Interface Science 332 (2024): 103267. 39121832 10.1016/j.cis.2024.103267

[125] P. Li , Y. F. Poon , W. Li , et al., “A Polycationic Antimicrobial and Biocompatible Hydrogel with Microbe Membrane Suctioning Ability,” Nature Materials 10 (2011): 149–156, 10.1038/nmat2915. 21151166

[126] K. V. R. Reddy , R. D. Yedery , and C. Aranha , “Antimicrobial Peptides: Premises and Promises,” International Journal of Antimicrobial Agents 24 (2004): 536–547, 10.1016/j.ijantimicag.2004.09.005. 15555874

[127] B. Yang , M. Li , Y. Wu , C. Deng , J. Cao , and H. Zhang , “Synthesis and Antimicrobial Activities of a Quaternary Ammonium Salt of Chitosan,” Asian Jouranl of Chemistry 24 (2012): 4022.

[128] Y. Ou , Z. Han , S. Cai , et al., “Biomaterials with Droplet Microfluidics,” Nature Reviews Bioengineering (2026).

[129] P. Lei , L. Luo , P. Guo , et al., “Microfluidic Design and Preparation of Hydrogel Microcapsules of Mesona chinensis Polysaccharide: Characterization, pH‐responsive Behavior and Gastrointestinal Protection for Lactobacillus Plantarum,” International Journal of Biological Macromolecules 301 (2025): 140446, 10.1016/j.ijbiomac.2025.140446. 39884599

[130] Q. Zhao , H. Cui , Y. Wang , and X. Du , “Microfluidic Platforms Toward Rational Material Fabrication for Biomedical Applications,” Small 16 (2020): 1903798. 10.1002/smll.201903798 31650698

[131] I. G. Loscertales , A. Barrero , I. Guerrero , R. Cortijo , M. Marquez , and A. M. Gañán‐Calvo , “Micro/Nano Encapsulation via Electrified Coaxial Liquid Jets,” Science 295 (2002): 1695. 11872835 10.1126/science.1067595

[132] J. Atencia and D. J. Beebe , “Controlled Microfluidic Interfaces,” Nature 437 (2005): 648–655, 10.1038/nature04163. 16193039

[133] P. Zhang , N. Shao , and L. Qin , “Recent Advances in Microfluidic Platforms for Programming Cell‐Based Living Materials,” Nature Reviews Bioengineering 33 (2021): 2005944. 10.1002/adma.202005944 34270839

[134] X. Hou , Y. S. Zhang , G. Trujillo‐de Santiago , et al., “Interplay between Materials and Microfluidics,” Nature Materials 2 (2017): 17016. 38993477 10.1038/natrevmats.2017.16 PMC11237287

[135] Y. Wei , X. Hou , J. Liu , Z. Han , and X. Mao , “Bioremediation of Heavy Metal Ion (Cu^2+^) by Live Probiotic Janus Microparticles Using Droplet‐based Microfluidic Technique,” Chemical Engineering Journal 502 (2024): 157855.

[136] D. W. Zheng , P. Pan , K. W. Chen , et al., “An Orally Delivered Microbial Cocktail for the Removal of Nitrogenous Metabolic Waste in Animal Models of Kidney Failure,” Nature Biomedical Engineering 4 (2020): 853–862, 10.1038/s41551-020-0582-1. 32632226

[137] S. Wu , J. Liu , J. Hou , et al., “Spatially Separated Dual‐Probiotic Hydrogel Enables Synergistic Infection Control and Tissue Healing,” Advanced Functional Materials 12 (2025): 24157.

[138] X. Yang , W. Nie , C. Wang , Z. Fang , and L. Shang , “Microfluidic‐based Multifunctional Microspheres for Enhanced Oral co‐delivery of Probiotics and Postbiotics,” Biomaterials 308 (2024): 122564. 38581763 10.1016/j.biomaterials.2024.122564

[139] K. Yang , X. Wang , R. Huang , H. Wang , P. Lan , and Y. Zhao , “Prebiotics and Postbiotics Synergistic Delivery Microcapsules from Microfluidics for Treating Colitis,” Advanced Science 9 (2022): 2104089, 10.1002/advs.202104089. 35403829 PMC9165482

[140] Y. Yin , Z. Li , H. Gao , et al., “Microfluidics‐Derived Microparticles with Prebiotics and Probiotics for Enhanced in Situ Colonization and Immunoregulation of Colitis,” Nano Letters 24 (2024): 1081–1089, 10.1021/acs.nanolett.3c03580. 38227962

[141] A. P. Dhand , M. D. Davidson , and J. A. Burdick , “Lithography‐based 3D Printing of Hydrogels,” Nature Reviews Bioengineering 3 (2025): 108–125, 10.1038/s44222-024-00251-9. PMC12269901 40678688

[142] L. M. Sidor and A. S. Meyer , “Additive Manufacturing of Engineered Living Materials,” Nature Reviews Materials 149 (2022): 149–185.

[143] Y. Ze , R. Wang , H. Deng , et al., “Three‐dimensional Bioprinting: a Cutting‐edge Tool for Designing and Fabricating Engineered Living Materials,” Biomaterials Advances 140 (2022): 213053, 10.1016/j.bioadv.2022.213053. 35964390

[144] K. S. Iyer , L. Bao , J. Zhai , et al., “Microgel‐based Bioink for Extrusion‐based 3D Bioprinting and Its Applications in Tissue Engineering,” Bioactive Materials 140 (2025): 273–293. 10.1016/j.bioactmat.2025.02.003 PMC11889356 40060146

[145] M. Schaffner , P. A. Rühs , F. Coulter , S. Kilcher , and A. R. Studart , “3D printing of Bacteria into Functional Complex Materials,” Science Advances 3 (2017): 6804, 10.1126/sciadv.aao6804. PMC5711516 29214219

[146] M. Jiang , J. Zheng , Y. Tang , et al., “Retrievable Hydrogel Networks with Confined Microalgae for Efficient Antibiotic Degradation and Enhanced Stress Tolerance,” Nature Communications 16 (2025): 3160, 10.1038/s41467-025-58415-z. PMC11965497 40175365

[147] H. Liu , H. Mei , H. Jiang , et al., “Bioprinted Symbiotic Dressings: a Lichen‐Inspired Approach to Diabetic Wound Healing with Enhanced Bioactivity and Structural Integrity,” Small 21 (2025): 2407105. 10.1002/smll.202407105 39663708

[148] L. M. González , N. Mukhitov , and C. A. Voigt , “Resilient Living Materials Built by Printing Bacterial Spores,” Nature Chemical Biology 16 (2020): 126–133, 10.1038/s41589-019-0412-5. 31792444

[149] A. Singh , J. Dorogin , K. Baker , et al., “Corked Microcapsules Enabling Controlled Ultrasound‐Mediated Protein Delivery,” ACS Applied Materials & Interfaces 16 (2024): 56199–56210. 39364661 10.1021/acsami.4c14615

[150] F. B. Haffner , T. van de Wiele , and A. Pasc , “Original Behavior of *L. rhamnosus* GG Encapsulated in Freeze‐Dried Alginate–Silica Microparticles Revealed under Simulated Gastrointestinal Conditions,” Journal of Materials Chemistry B 5 (2017): 7839–7847, 10.1039/C7TB02190A. 32264385

[151] J. Tailleur and M. E. Cates , “Statistical Mechanics of Interacting Run‐and‐Tumble Bacteria,” Physical Review Letter 100 (2008): 218103. 10.1103/PhysRevLett.100.218103 18518641

[152] T. Bhattacharjee and S. S. Datta , “Bacterial Hopping and Trapping in Porous media,” Nature Communications 10 (2019): 2075. 10.1038/s41467-019-10115-1 PMC6502825 31061418

[153] J. Maennik , R. Driessen , P. Galajda , J. E. Keymer , and C. Dekker , “Bacterial Growth and Motility in Sub‐micron Constrictions,” Proceedings of the National Academy of Sciences 106 (2009): 14861–14866, 10.1073/pnas.0907542106. PMC2729279 19706420

[154] F. J. H. Hol and C. Dekker , “Zooming in to See the Bigger Picture: Microfluidic and Nanofabrication Tools to Study Bacteria,” Science 346 (2014): 438, 10.1126/science.1251821. 25342809

[155] S. Takeuchi , W. R. DiLuzio , D. B. Weibel , and G. M. Whitesides , “Controlling the Shape of Filamentous Cells of Escherichia coli,” Nano Letters 5 (2005): 1819–1823, 10.1021/nl0507360. 16159230 PMC2519610

[156] H. Priks , T. Butelmann , A. Illarionov , et al., “Physical Confinement Impacts Cellular Phenotypes within Living Materials,” ACS Applied Bio Materials 3 (2020): 4273–4281. 10.1021/acsabm.0c00335 PMC7375193 32715284

[157] R. Z. Moger‐Reischer and J. T. Lennon , “Microbial Ageing and Longevity,” Nature Reviews Microbiology 17 (2019): 679–690, 10.1038/s41579-019-0253-y. 31534207

[158] A. J. Paula , G. Hwang , and H. Koo , “Dynamics of Bacterial Population Growth in Biofilms Resemble Spatial and Structural Aspects of Urbanization,” Nature Communications 11 (2020): 1354. 10.1038/s41467-020-15165-4 PMC7070081 32170131

[159] L. J. Holt , O. Hallatschek , and M. Delarue , “Chapter 12 ‐ Mechano‐chemostats to Study the Effects of Compressive Stress on Yeast,” Methods in Cell Biology 147 (2018): 215–231. 30165959 10.1016/bs.mcb.2018.06.010

[160] B. Kaehr and J. B. Shear , “Multiphoton Fabrication of Chemically Responsive Protein Hydrogels for Microactuation,” Proceedings of the National Academy of Sciences 105 (2008): 8850–8854, 10.1073/pnas.0709571105. PMC2449329 18579775

[161] J. L. Connell , E. T. Ritschdorff , M. Whiteley , and J. B. Shear , “3D printing of Microscopic Bacterial Communities,” Proceedings of the National Academy of Sciences 110 (2013): 18380–18385, 10.1073/pnas.1309729110. PMC3832025 24101503

[162] J. Yang , M. Peng , S. Tan , et al., “Calcium Tungstate Microgel Enhances the Delivery and Colonization of Probiotics during Colitis via Intestinal Ecological Niche Occupancy,” ACS Central Science 9 (2023): 1327–1341, 10.1021/acscentsci.3c00227. 37521784 PMC10375893

[163] Y. Fu , T. Wang , X. Ge , et al., “Orally‐deliverable Liposome‐microgel Complexes Dynamically Remodel Intestinal Environment to Enhance Probiotic Ulcerative Colitis Therapy via TLR4 Inhibition and Tryptophan Metabolic Crosstalk,” Biomaterials 321 (2025): 123339. 40233710 10.1016/j.biomaterials.2025.123339

[164] G. Fan , Y. Lu , Y. Li , et al., “Lactobacillus‐Loaded Easily Injectable Hydrogel Promotes Endometrial Repair via Long‐Term Retention and Microenvironment Modulation,” ACS Nano 19 (2025): 4440–4451. 39823410 10.1021/acsnano.4c13593

[165] Y. Sun , M. Liu , X. Tang , Y. Zhou , J. Zhang , and B. Yang , “Culture‐Delivery Live Probiotics Dressing for Accelerated Infected Wound Healing,” ACS Applied Materials & Interfaces 15 (2023): 53283–53296, 10.1021/acsami.3c12845. 37948751

[166] O. Hasturk and D. L. Kaplan , “Cell Armor for Protection against Environmental Stress: Advances, Challenges and Applications in Micro‐ and Nanoencapsulation of Mammalian Cells,” Acta Biomaterialia 95 (2019): 3–31, 10.1016/j.actbio.2018.11.040. 30481608 PMC6534491

[167] Y. Xiao , C. Lu , Y. Liu , et al., “Encapsulation of *Lactobacillus Rhamnosus* in Hyaluronic Acid‐Based Hydrogel for Pathogen‐Targeted Delivery to Ameliorate Enteritis,” ACS Applied Materials & Interfaces 12 (2020): 36967–36977, 10.1021/acsami.0c11959. 32702229

[168] W. Li , X. Luo , R. Song , Y. Zhu , B. Li , and S. Liu , “Porous Cellulose Microgel Particle: a Fascinating Host for the Encapsulation, Protection, and Delivery of Lactobacillus Plantarum,” Journal of Agricultural and Food Chemistry 64 (2016): 3430. 27068772 10.1021/acs.jafc.6b00481

[169] W. Li , L. Liu , H. Tian , X. Luo , and S. Liu , “Encapsulation of Lactobacillus Plantarum in Cellulose Based Microgel with Controlled Release Behavior and Increased Long‐term Storage Stability,” Carbohydrate Polymers 223 (2019): 115065. 31426953 10.1016/j.carbpol.2019.115065

[170] J. Su , X. Wang , W. Li , et al., “Enhancing the Viability of Lactobacillus Plantarum as Probiotics through Encapsulation with High Internal Phase Emulsions Stabilized with Whey Protein Isolate Microgels,” Journal of Agricultural and Food Chemistry 66 (2018): 12335–12343, 10.1021/acs.jafc.8b03807. 30380846

[171] J. Qiu , S. Xiang , M. Sun , and M. Tan , “Preparation of Polysaccharide–Protein Hydrogels with an Ultrafast Self‐Healing Property as a Superior Oral Delivery System of Probiotics,” Journal of Agricultural and Food Chemistry 71 (2023): 18842–18856, 10.1021/acs.jafc.3c05898. 37978937

[172] C. Hua , F. Yang , X. Jia , et al., “Multi‐comparted Microgels Delivering human Derived Probiotics and Deferoxamine for Multidrug‐resistant Infection and Healing,” Chemical Engineering Journal 483 (2024): 148432, 10.1016/j.cej.2023.148432.

[173] L. Gao , L. Feng , D. F. Sauer , et al., “Engineered Living Hydrogels for Robust Biocatalysis in Pure Organic Solvents,” Cell Reports Physical Science 3 (2022): 101054, 10.1016/j.xcrp.2022.101054.

[174] S. Manan , M. W. Ullah , M. Ul‐Islam , Z. Shi , M. Gauthier , and G. Yang , “Bacterial Cellulose: Molecular Regulation of Biosynthesis, Supramolecular Assembly, and Tailored Structural and Functional Properties,” Progress in Materials Science 129 (2022): 100972, 10.1016/j.pmatsci.2022.100972.

[175] Y. Zhang , D. W. Sun , and L. Xu , “Bacterial Cellulose‐driven Sustainable Food Packaging Innovations: Biosynthesis, Functionalization, and Applications,” Trends in Food Science & Technology 163 (2025): 105167, 10.1016/j.tifs.2025.105167.

[176] P. Praveschotinunt , A. M. Duraj‐Thatte , I. Gelfat , F. Bahl , D. B. Chou , and N. S. Joshi , “Engineered E. coli Nissle 1917 for the Delivery of Matrix‐tethered Therapeutic Domains to the Gut,” Nature Communications 10 (2019): 5580. 10.1038/s41467-019-13336-6 PMC6898321 31811125

[177] Y. Xu , K. Wei , L. Bian , G. Li , and C. Zhang , “High‐yield Bacterial Cellulose Production from Rice Bran Using a Genetically Characterized Komagataeibacter Europaeus Strain,” International Journal of Biological Macromolecules 310 (2025): 143201, 10.1016/j.ijbiomac.2025.143201. 40253037

[178] D. Mikkelsen , B. M. Flanagan , G. A. Dykes , and M. J. Gidley , “Influence of Different Carbon Sources on Bacterial Cellulose Production by Gluconacetobacter Xylinus Strain ATCC 53524,” Journal of Applied Microbiology 107 (2009): 576–583, 10.1111/j.1365-2672.2009.04226.x. 19302295

[179] S. Wang , T. Li , C. Chen , et al., “Transparent, Anisotropic Biofilm with Aligned Bacterial Cellulose Nanofibers,” Advanced Functional Materials 28 (2018): 1707491, 10.1002/adfm.201707491.

[180] S. Obuobi and N. Škalko‐Basnet , “Understanding Vaginal Biofilms: the First Step in Harnessing Antimicrobial Nanomedicine,” Journal of Controlled Release 376 (2024): 1190–1208, 10.1016/j.jconrel.2024.10.064. 39510257

[181] C. W. Hall and T. F. Mah , “Molecular Mechanisms of Biofilm‐based Antibiotic Resistance and Tolerance in Pathogenic Bacteria,” FEMS Microbiology Reviews 41 (2017): 276–301, 10.1093/femsre/fux010. 28369412

[182] Y. Gui , Q. Sun , K. Li , et al., “Bioinspired Gelated Cell Sheet–supported lactobacillus Biofilm for Aerobic Vaginitis Diagnosis and Treatment,” Science Advances 10 (2024): 2732, 10.1126/sciadv.adq2732. PMC11529721 39485840

[183] N. Sajankila , Z. Dumbauld , Y. Wang , et al., “Biofilm state Limosilactobacillus Reuteri Modulates Aryl Hydrocarbon Receptor Activity and Suppresses Experimental Necrotizing Enterocolitis,” Pediatric Research 99 (2025): 1137–1144. 40877707 10.1038/s41390-025-04351-z PMC13021507

[184] S. Y. Kang , A. Pokhrel , S. Bratsch , et al., “Engineering *Bacillus subtilis* for the formation of a durable living biocomposite material,” Nature Communications 12 (2021): 7133, 10.1038/s41586-021-03839-y. PMC8654922 34880257

[185] J. Caro‐Astorga , K. T. Walker , N. Herrera , K. Y. Lee , and T. Ellis , “Bacterial Cellulose Spheroids as Building Blocks for 3D and Patterned Living Materials and for Regeneration,” Nature Communications 12 (2021): 5027. 10.1038/s41467-021-25350-8 PMC8377073 34413311

[186] C. M. Heveran , S. L. Williams , J. Qiu , et al., “Biomineralization and Successive Regeneration of Engineered Living Building Materials,” Matter 2 (2020): 481–494.

[187] J. Qiu , J. Artier , S. Cook , W. V. Srubar III , J. C. Cameron , and M. H. Hubler , “Engineering Living Building Materials for Enhanced Bacterial Viability and Mechanical Properties,” iScience 24 (2021): 102083. 33598643 10.1016/j.isci.2021.102083 PMC7868992

[188] G. Jiang , M. Chen , W. Sheng , et al., “Microbial Fermented Nanostructured Hydrogels for Tough, Self‐healing Wearable Sensors,” Chemical Engineering Journal 515 (2025): 163931, 10.1016/j.cej.2025.163931.

[189] Z. Dai , X. Yang , F. Wu , et al., “Living Fabrication of Functional Semi‐interpenetrating Polymeric Materials,” Nature Communications 12 (2021): 3422. 10.1038/s41467-021-23812-7 PMC8187375 34103521

[190] H. L. Ehrlich , “How Microbes Influence Mineral Growth and Dissolution,” Chemical Geology 132 (1996): 5–9, 10.1016/S0009-2541(96)00035-6.

[191] X. Wang , Z. Cao , M. Zhang , L. Meng , Z. Ming , and J. Liu , “Bioinspired Oral Delivery of Gut Microbiota by Self‐Coating with Biofilms,” Science Advances 6 (2020): abb1952. 10.1126/sciadv.abb1952 PMC7314526 32637620

[192] K. Yu , Z. Feng , H. Du , et al., “Photosynthesis‐Assisted Remodeling of Three‐Dimensional Printed Structures,” PANS 118 (2021): 2016524118. 10.1073/pnas.2016524118 PMC7826334 33431680

[193] S. R. McCuskey , G. Quek , R. J. Vázquez , et al., “Evolving Synergy between Synthetic and Biotic Elements in Conjugated Polyelectrolyte/Bacteria Composite Improves Charge Transport and Mechanical Properties,” Advanced Science 11 (2024): 2405242, 10.1002/advs.202405242. 39262122 PMC11558123

[194] B. H. A. Rehm , “Bacterial Polymers: Biosynthesis, Modifications and Applications,” Nature Reviews Microbiology 8 (2020): 578–592, 10.1038/nrmicro2354. 20581859

[195] T. J. Hossain , S. I. Chowdhury , H. A. Mozumder , et al., “Hydrolytic Exoenzymes Produced by Bacteria Isolated and Identified from the Gastrointestinal Tract of Bombay Duck,” Frontiers in Microbiology 11 (2020): 2097. 32983064 10.3389/fmicb.2020.02097 PMC7479992

[196] S. Sadhu and T. K. J. B. Maiti , “Cellulase Production by Bacteria: A Review,” British Microbiology Research Journal 3 (2013): 235–258.

[197] C. Dussud , C. Hudec , M. George , et al., “Colonization of Non‐Biodegradable and Biodegradable Plastics by Marine Microorganisms,” Frontiers in Microbiology 9 (2018): 1571. 30072962 10.3389/fmicb.2018.01571 PMC6058052

[198] L. D. L. Jenkins , K. A. Cook , and R. B. Cain , “Microbial Degradation of Polyethylene Glycols,” Journal of Applied Bacteriology 47 (1979): 75–85, 10.1111/j.1365-2672.1979.tb01171.x. 500514

[199] G. F. White , N. J. Russell , and E. C. Tidswell , “Bacterial Scission of Ether Bonds,” Microbiological Reviews 60 (1996): 216–232, 10.1128/mr.60.1.216-232.1996. 8852901 PMC239425

[200] S. Yoshida , K. Hiraga , T. Takehana , et al., “A Bacterium That Degrades and Assimilates Poly(ethylene terephthalate),” Science 351 (2016): 1196–1199, 10.1126/science.aad6359. 26965627

[201] H. Zhang , J. Zhang , B. Liu , et al., “Natural Phenolic‐Metal Framework Strengthened Mesona Chinensis Polysaccharides Microgels for Improved Viability of Probiotics to Alleviate the Liver Injury and Gut Microbiota Dysbiosis,” Advanced Functional Materials 34 (2024): 2401064, 10.1002/adfm.202401064.

[202] A. Roy , M. Patra , S. Sarkhel , et al., “Fucose‐containing Abroma Augusta Mucilage Hydrogel as a Potential Probiotic Carrier with Prebiotic Function,” Food Chemistry 387 (2022): 132941, 10.1016/j.foodchem.2022.132941. 35430541

[203] S. Du , R. Sun , M. Wang , et al., “Synergistic Effect of Inulin Hydrogels on Multi‐strain Probiotics for Prevention of Ionizing Radiation‐induced Injury,” International Journal of Biological Macromolecules 287 (2025): 138497, 10.1016/j.ijbiomac.2024.138497. 39647719

[204] M. Duan , L. Che , X. Wu , et al., “Incorporation of Probiotics with Pressure‐Sensitive Pectin‐Fructooligosaccharide Hydrogel for Potential Intestinal Delivery,” Carbohydrate Polymers 359 (2025): 123566, 10.1016/j.carbpol.2025.123566. 40306774

[205] A. Xie , H. Ji , Z. Liu , et al., “Modified Prebiotic‐Based “Shield” Armed Probiotics with Enhanced Resistance of Gastrointestinal Stresses and Prolonged Intestinal Retention for Synergistic Alleviation of Colitis,” ACS Nano 17 (2023): 14775–14791, 10.1021/acsnano.3c02914. 37477584

[206] H. Li , L. Ma , N. Zhu , et al., “Natural‐Origin Bioadhesive Hydrogel with Dual Antioxidative and Immunoregulatory Properties for Enhanced Angiogenesis and Wound Healing,” Bioactive Materials 53 (2025): 507–521. 40755848 10.1016/j.bioactmat.2025.07.023 PMC12313959

[207] H. Chen , Z. Zhao , R. Zhang , et al., “Adaptable Hydrogel with Strong Adhesion of Wet Tissue for Long‐Term Protection of Periodontitis Wound,” Advanced Materials 37 (2025): 2413373, 10.1002/adma.202413373. 39568256

[208] G. Chen , F. Wang , X. Zhang , Y. Shang , and Y. Zhao , “Living Microecological Hydrogels for Wound Healing,” Science Advances 9 (2023): adg3478. 10.1126/sciadv.adg3478 PMC10208562 37224242

[209] P. Zhang , Z. Fan , P. Cheng , F. Tian , Z. Wang , and J. Han , “Dynamic Hydrazone Crosslinked Salecan/Chondroitin Sulfate Hydrogel Platform as a Promising Wound Healing Strategy: a Comparative Study on Antibiotic and Probiotic Delivery,” International Journal of Pharmaceutics 665 (2024): 124667, 10.1016/j.ijpharm.2024.124667. 39241931

[210] Y. M. Algavi and E. Borenstein , “Relative Dispersion Ratios Following Fecal Microbiota Transplant Elucidate Principles Governing Microbial Migration Dynamics,” Nature Communications 15 (2024): 4447. 10.1038/s41467-024-48717-z PMC11126695 38789466

[211] S. F. Mao , Y. M. Ren , W. Tan , N. Donlao , X. Q. Ye , and J. H. Tian , “,” Small (2025). 10.1002/smll.202503476 40968559

[212] S. Liu , M. Yang , C. Smarr , G. Zhang , H. Barton , and W. Xu , “Engineered Living Structures with Shape‐Morphing Capability Enabled by 4D Printing with Functional Bacteria,” ACS Applied Bio Materials 7 (2024): 3247–3257, 10.1021/acsabm.4c00223. 38648508

[213] W. Shan , Y. Liang , J. Liu , et al., “Oral Administration of TNF‐α‐modulating Probiotics Integrated by Dual‐layered Microcapsule for Effective Treatment of Inflammatory Bowel Disease,” Chemical Engineering Journal 521 (2025): 166413, 10.1016/j.cej.2025.166413.

[214] Y. Qiu , Y. Zeng , C. Zhang , et al., “A ROS‐responsive Loaded Desferoxamine (DFO) Hydrogel System for Traumatic Brain Injury Therapy,” Biomedical Materials 19 (2024): 025016. 10.1088/1748-605X/ad1dfd 38215474

[215] X. Tang , A. Li , X. Cheng , et al., “Antioxidant‐Targeting Synergistic Probiotic Delivery System with Improved Probiotics Viability and Intestinal Retention for Ulcerative Colitis Therapy,” ACS Applied Polymer Materials 7 (2025): 9693–9713, 10.1021/acsapm.5c01178.

[216] U. Shedaliya , G. Adwani , T. R. Anju , A. Krishnakumar , and A. Kumar , “Role of the Microbiome in Diabetic Wound Healing: Implications for New Therapeutic Approaches,” Archives of Microbiology 207 (2025): 208, 10.1007/s00203-025-04399-9. 40742547

[217] X. Liu , W. Wang , Y. Wang , et al., “Biochemical Strategy‐based Hybrid Hydrogel Dressing‐mediated In Situ Synthesis of Selenoproteins for DFU Immunity‐microbiota Homeostasis Regulation,” Biomaterials 317 (2025): 123114. 39854881 10.1016/j.biomaterials.2025.123114

[218] X. Yang , T. Che , S. Tian , et al., “A Living Microecological Hydrogel with Microbiota Remodeling and Immune Reinstatement for Diabetic Wound Healing,” Advanced Healthcare Materials 13 (2024): 2400856, 10.1002/adhm.202400856. 38744431

[219] X. Dou , G. Li , S. Wang , et al., “Probiotic‐loaded Calcium Alginate/Fucoidan Hydrogels for Promoting Oral Ulcer Healing,” International Journal of Biological Macromolecules 244 (2023): 125273, 10.1016/j.ijbiomac.2023.125273. 37301354

[220] A. P. Liu , E. A. Appel , P. D. Ashby , et al., “The Living Interface between Synthetic Biology and Biomaterial Design,” Nature Materials 21 (2022): 390–397, 10.1038/s41563-022-01231-3. 35361951 PMC10265650

[221] T. C. Tang , B. An , Y. Huang , et al., “Materials Design by Synthetic Biology,” Nature Reviews Materials 6 (2021): 332–350, 10.1038/s41578-020-00265-w.

[222] Y. Lu , H. Li , J. Wang , et al., “Engineering Bacteria‐Activated Multifunctionalized Hydrogel for Promoting Diabetic Wound Healing,” Advanced Functional Materials 31 (2021): 2105749.

[223] L. Li , C. Yang , B. Ma , et al., “Hydrogel‐Encapsulated Engineered Microbial Consortium as a Photoautotrophic “Living Material” for Promoting Skin Wound Healing,” ACS Applied Materials & Interfaces 15 (2023): 6536–6547, 10.1021/acsami.2c20399. 36708324

[224] E. Zhao , H. Liu , Y. Jia , et al., “Engineering a Photosynthetic Bacteria‐incorporated Hydrogel for Infected Wound Healing,” Acta Biomaterialia 140 (2022): 302–313, 10.1016/j.actbio.2021.12.017. 34954107

[225] A. L. Mueller , A. Brockmueller , N. Fahimi , et al., “Bacteria‐Mediated Modulatory Strategies for Colorectal Cancer Treatment,” Biomedicines 10 (2022): 832. 35453581 10.3390/biomedicines10040832 PMC9026499

[226] Y. Wang , X. Su , Y. Liu , et al., “Improved Prebiotic‐based “Shield” Equipped Probiotics for Enhanced Colon Cancer Therapy by Polarizing M1 Macrophages and Regulating Intestinal Microbiota,” Acta Pharm Sin B 15 (2025): 4225–4247. 40893693 10.1016/j.apsb.2025.05.040 PMC12399202

[227] W. Y. Wang , W. J. Yu , G. R. Li , et al., “Engineering versatile nano‐bacteria hybrids for efficient tumor therapy,” Coordin Chem Rev 488 (2023): 215178.

[228] A. Redenti , J. Im , B. Redenti , et al., “Probiotic Neoantigen Delivery Vectors for Precision Cancer Immunotherapy,” Nature 635 (2024): 453–461, 10.1038/s41586-024-08033-4. 39415001 PMC11560847

[229] L. Niu , Y. Liu , N. Li , et al., “Oral Probiotics Microgel plus Galunisertib Reduced TGF‐β Blockade Resistance and Enhanced Anti‐tumor Immune Responses in Colorectal Cancer,” International Journal of Pharmaceutics 652 (2024): 123810, 10.1016/j.ijpharm.2024.123810. 38244648

[230] Z. Yang , Y. Zhu , Z. Dong , et al., “Engineering Bioluminescent Bacteria to Boost Photodynamic Therapy and Systemic Anti‐Tumor Immunity for Synergistic Cancer Treatment,” Biomaterials 281 (2022): 121332. 35066286 10.1016/j.biomaterials.2021.121332

[231] F. S. Fu , H. H. Chen , Y. Chen , et al., “Engineered bacillus Subtilis Enhances Bone Regeneration via Immunoregulation and Anti‐Infection,” Bioactive Materials 46 (2025): 503–515. 39868074 10.1016/j.bioactmat.2025.01.003 PMC11760808

[232] K. Li , B. Li , J. Li , et al., “Chairside Live Biotherapeutic Hydrogel for Comprehensive Periodontitis Therapy,” Trends in Biotechnology 43 (2025): 408–432, 10.1016/j.tibtech.2024.10.001. 39505614

[233] P. Sagar , V. Singh , R. Gupta , et al., “pH‐Triggered, Synbiotic Hydrogel Beads for in Vivo Therapy of Iron Deficiency Anemia and Reduced Inflammatory Response,” ACS Applied Bio Materials 4 (2021): 7467–7484, 10.1021/acsabm.1c00720. 35006707

[234] N. Nezamdoost‐Sani , M. A. Khaledabad , S. Amiri , Y. Phimolsiripol , and A. M. Khaneghah , “A Comprehensive Review on the Utilization of Biopolymer Hydrogels to Encapsulate and Protect Probiotics in Foods,” International Journal of Biological Macromolecules 254 (2024): 127907. 37935287 10.1016/j.ijbiomac.2023.127907

[235] R. Wang , K. Guo , W. Zhang , et al., “Poly‐γ‐Glutamic Acid Microgel‐Encapsulated Probiotics with Gastric Acid Resistance and Smart Inflammatory Factor Targeted Delivery Performance to Ameliorate Colitis,” Advanced Functional Materials 32 (2022): 2113034.

[236] J. L. Patarroyo , J. S. Florez‐Rojas , D. Pradilla , J. D. Valderrama‐Rincon , J. C. Cruz , and L. H. Reyes , “Formulation and Characterization of Gelatin‐Based Hydrogels for the Encapsulation of Kluyveromyces Lactis—Applications in Packed‐Bed Reactors and Probiotics Delivery in Humans,” Polymers 12 (2020): 1287. 32512791 10.3390/polym12061287 PMC7362005

[237] K. F. Fan , K. Li , L. W. L. Han , et al., “Multifunctional double‐network Ti3C2Tx MXene composite hydrogels for strain sensors with effective electromagnetic interference and UV shielding properties,” Polymers 273 (2023): 125865.

[238] D. M. Xie , Curr Op Biotechnol 78 (2022): 102793. 10.1016/j.copbio.2022.102793 36088736

[239] Y. Yu , F. J. Xie , X. P. Gao , and L. Q. Zheng , “Double‐network Hydrogels with Adjustable Surface Morphology and Multifunctional Integration for Flexible Strain Sensors,” Soft Matter 17 (2021): 4352–4362, 10.1039/D1SM00158B. 33908588

[240] X. X. Qi , S. Simsek , B. C. Chen , and J. J. Rao , “Alginate‐based Double‐network Hydrogel Improves the Viability of Encapsulated Probiotics during Simulated Sequential Gastrointestinal Digestion: Effect of Biopolymer Type and Concentrations,” International Journal of Biological Macromolecules 165 (2020): 1675–1685, 10.1016/j.ijbiomac.2020.10.028. 33058979

[241] T. W. Yeung , I. J. Arroyo‐Maya , D. J. McClements , and D. A. Sela , “Microencapsulation of Probiotics in Hydrogel Particles: Enhancing Lactococcus Lactis subsp. Cremoris LM0230 Viability Using Calcium Alginate Beads,” Food & Function 7 (2016): 1797–1804, 10.1039/C5FO00801H. 26611443

[242] K. Cerk and M. Aguilera‐Gómez , “Microbiota Analysis for Risk Assessment: Evaluation of Hazardous Dietary Substances and Its Potential Role on the Gut Microbiome Variability and Dysbiosis,” EFSA Journal 20 (2022): 200404. 10.2903/j.efsa.2022.e200404 PMC9131584 35634548

[243] M. Cordaillat‐Simmons , A. Rouanet , and B. Pot , “Live Biotherapeutic Products: the Importance of a Defined Regulatory Framework,” Experimental & Molecular Medicine 52 (2020): 1397–1406, 10.1038/s12276-020-0437-6. 32908212 PMC8080583

[244] Microbiome Therapeutics Innovation Group , D. Barberio , “Navigating Regulatory and Analytical Challenges in Live Biotherapeutic Product Development and Manufacturing,” Front Microbiomes 3 (2024): 1441290. 41853515 10.3389/frmbi.2024.1441290 PMC12993642

[245] G. Franciosa , S. Guida , M. J. G. Miguel , and C. Von Hunolstein , “Live Biotherapeutic Products and Their Regulatory Framework in Italy and Europe,” Annali dell'Istituto Superiore di Sanita 59 (2023): 56–67. 10.4415/ANN_23_01_09 36974706

